# Simulation of gravity- and pump-driven perfusion techniques for measuring outflow facility of ex vivo and in vivo eyes

**DOI:** 10.1371/journal.pone.0294607

**Published:** 2023-11-21

**Authors:** Youssef Mohamed, Christopher L. Passaglia

**Affiliations:** 1 Department of Medical Engineering, University of South Florida, Tampa, FL, United States of America; 2 Department of Ophthalmology, University of South Florida, Tampa, FL, United States of America; Duke University, UNITED STATES

## Abstract

Aqueous humor dynamics are commonly assessed by infusing fluid into the eye and measuring intraocular pressure (IOP). From the pressure-flow relationship, conventional outflow facility is estimated to study glaucomatous processes that lower facility or identify therapeutics that enhance facility in hopes of restoring healthy IOP levels. The relative merits and limitations of constant flow (CF), gravity-driven constant pressure (CPg), and pump-driven constant pressure (CPp) infusion techniques were explored via simulations of a lumped parameter viscoelastic model of the eye. Model parameter values were based on published perfusion system properties and outflow facility data from rodents. Step increases in pressure or flow were simulated without and with IOP noise recorded from enucleated eyes, anesthetized animals, and conscious animals. Steady-state response levels were determined using published window and ratio criteria. Model simulations show that all perfusion techniques estimate facility accurately and that ocular fluid dynamics set a hard limit on how fast measurements can be taken. This limit can be approached with CPg and CPp systems by increasing their gain but not with CF systems, which invariably take longest to settle. Facility experiment duration is further lengthened by inclusion of IOP noise, and data filtering is needed for steady-state detection with in vivo noise. The ratio criterion was particularly affected because noise in the flow data is amplified by the higher gain of CPg and CPp systems. A recursive regression method is introduced, which can ignore large transient IOP fluctuations that interfere with steady-state detection by fitting incoming data to the viscoelastic eye model. The fitting method greatly speeds up data collection without loss of accuracy, which could enable outflow facility measurements in conscious animals. The model may be generalized to study response dynamics to fluid infusion in other viscoelastic compartments of the body and model insights extended to optimize experiment design.

## Introduction

Glaucoma is the leading cause of irreversible blindness worldwide [1–3]. The disease is characterized by progressive degeneration of the retina and optic nerve, oftentimes in association with high intraocular pressure (IOP) [4]. Lowering IOP is therefore a central focus of clinical treatment [5–7]. Many factors combine to determine IOP at a given moment, such as aqueous humor production rate, episcleral venous pressure, and ocular compliance. Foremost to glaucoma management is aqueous humor outflow resistance, as impaired drainage can lead to elevated IOP and trabecular meshwork facility and uveoscleral drainage are reduced in ocular hypertensive patients [8]. In addition, several glaucoma medicines lower IOP by decreasing the fluidic resistance of outflow pathways [6, 9–11].

A method of monitoring outflow facility would be useful for investigating how glaucoma alters ocular fluid dynamics as the disease progresses. The clinical approach is to instill fluorescein eye drops, estimate aqueous outflow from the dilution of dye over time in the anterior chamber, and calculate outflow facility from changes in IOP and flow rate induced by instillation of pharmacological agents. The noninvasive nature of fluorophotometry is attractive, but a single facility measurement can take hours and vary markedly owing to differences in ocular properties across subjects [12, 13]. Tonography is also used clinically, though facility measurements can exhibit limited repeatability [13, 14]. The direct approach is to cannulate the eye with a needle, infuse fluid, and estimate outflow facility from the steady-state relationship between infusion rate and IOP. The approach is too invasive for clinical settings, but the greater accuracy and speed make it useful as a research tool in animal models. The infusion rate can either be fixed to hold aqueous flow constant [15] or continually adjusted to hold IOP constant [16]. The constant flow (CF) technique is simple to implement, but IOP can take a while to stabilize after flow rate changes [17]. The constant pressure (CP) technique can speed up data collection by delivering flow at variable rates via an adjustable-height fluid reservoir [18, 19] or a feedback-controlled pump [20, 21], but the setup involves more technology and oversight. CF and CP techniques have the disadvantage that the eye must be enucleated or the subject anesthetized for needle cannulation, which limits measurement duration and frequency. Anesthetics and repeated cannulations could have adverse or unintended effects on research outcomes as well [22, 23].

This study uses computer simulations to identify key design parameters and functional limitations of infusion-based techniques with the goal of creating an eye perfusion system that can monitor ocular fluid dynamics in conscious animals. A viscoelastic model of the eye and eye perfusion system is presented, and outflow facility experiments are simulated on the model eye for different perfusion systems (CF, gravity-driven CP, pump-driven CP) that have been published. Facility measurement speed, accuracy, and other performance attributes are examined for each system in the absence and presence of physiologically-realistic IOP noise. To overcome performance deterioration at heightened noise levels, a novel recursive regression algorithm is introduced that can estimate outflow facility rapidly and accurately in the face of IOP variability encountered in awake free-moving animals.

## Materials and methods

### Modeling eye perfusion techniques

**Fig 1:**
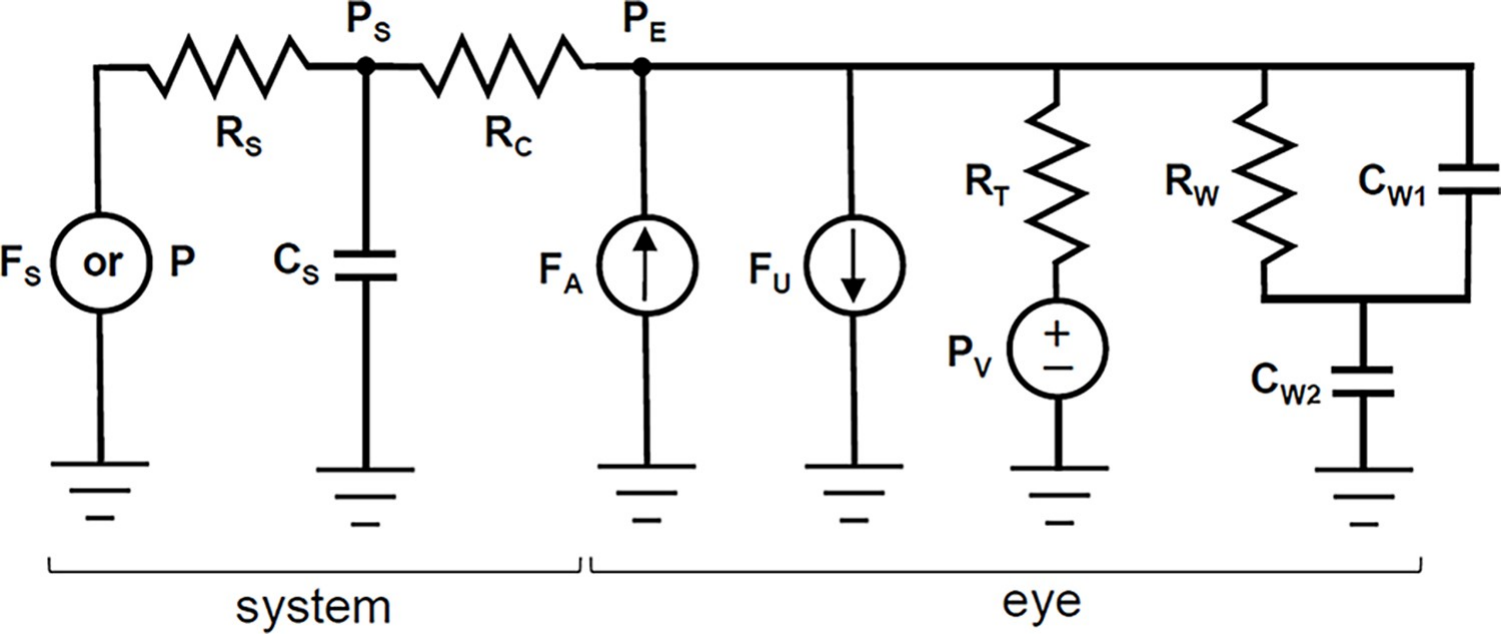
Lumped-parameter viscoelastic model of an eye perfusion system. F_S_, fluid infusion rate (pump-driven system); P, reservoir pressure (gravity-driven system); R_S_ and C_S_, system resistance and compliance; P_S_, system pressure; R_C_, cannula resistance; P_E_, intraocular pressure; F_A_, aqueous production rate; F_U_, uveoscleral outflow rate; R_T_, trabecular outflow resistance; P_V_, episcleral venous pressure; R_W_ and C_W_, resistance and compliance of globe wall, where 1/C_W_ = 1/C_W1_ + 1/C_W2_.

Common eye perfusion techniques were implemented as three computational models (CF, CPg, and CPp). The CF model mimics setting a pump to infuse fluid into the eye at constant rate and recording resultant changes in IOP [17]. The CPg model mimics holding IOP constant with a fluid reservoir set at adjustable height and recording flow driven into the eye by gravity [18, 24]. The CPp model mimics holding IOP constant with a pump that removes deviations of recorded IOP from a target level by dynamically adjusting flow rate [20, 21]. The models are described by the same lumped-parameter circuit representation of the eye and perfusion system shown in Fig 1, where perfusion is driven by a flow source F_S_ for CF and CPp models and a pressure source P for CPg models. Fluid flows into the model eye at rate F_E_ through a microcannula of resistance R_C_ and system resistance R_S_ and compliance C_S_. The flow causes pressure in the system P_S_ to differ slightly from pressure in the eye, which is termed P_E_ in model simulations to avoid confusion with IOP measured physiologically. In the CF and CPp models, R_S_ can be neglected because the high input impedance of a pump prevents fluid backflow. In CPg models, the system has a flow sensor that primarily dictates R_S_. P_E_ and F_E_ can be calculated at any point in time from knowledge of P_S_ and system parameters via:

$$P_{E}(t)=R_{C}C_{S}\frac{dP_{S}(t)}{dt}+P_{S}(t)-{R_{C}F}_{S}(P_{S},t)$$ [1]

$$F_{E}(t)=F_{S}(t)-C_{S}\frac{dP_{S}(t)}{dt}=({P_{S}(t)-P}_{E}(t))/R_{C}$$ [2]

The eye is considered a pressurized vessel with homogeneous viscoelastic walls of fluidic resistance R_W_ and compliance C_W_. The viscoelasticity reflects the time-dependent response of the globe to biomechanical loading and unloading, which can delay volume equilibration after pressure changes [25, 26]. It was described with a standard linear solid model, which has been shown to effectively and efficiently reproduce the stress-strain behavior of corneal tissue [27–29]. The model is a second-order approximation of globe viscoelastic properties, as wall compliance is distributed over two elements C_W1_ and C_W2_ whereas scleral specimens exhibit multiple relaxation times under tensile testing [27, 28]. A purely elastic globe can also be simulated by setting R_W_ to zero.

Eye pressurization is maintained by the continual production of aqueous humor at flow rate F_A_. Some aqueous fluid leaks out the eye through relatively pressure-independent uveoscleral pathways at flow rate F_U_. The rest exits through the conventional trabecular pathway with hydraulic resistance R_T_ into episcleral drainage veins of pressure P_V_. Since F_A_, F_U_, and P_V_ are roughly constant over short time scales [30–32], they can be combined via the Goldmann equation into ${\bar{P}}_{E}$ (i.e., resting IOP). The relationship between P_S_ and P_E_, as dictated by ocular fluid dynamics, is then:

$$\alpha \frac{d^{2}P_{E}(t)}{{dt}^{2}}+(\frac{\beta }{R_{C}})\frac{dP_{E}(t)}{dt}+(\frac{{R_{C}+R}_{T}}{R_{C}})P_{E}(t)=(\frac{R_{T}R_{W}(C_{W1}+C_{W2})}{R_{C}})\frac{{dP}_{S}(t)}{dt}+(\frac{R_{T}}{R_{C}})P_{S}(t)+{\bar{P}}_{E},$$ [3]

where *α* = *R*_*T*_*R*_*W*_*C*_*W*1_*C*_*W*2_ and $\beta ={R_{C}R}_{W}C_{W1}+{{R_{C}R}_{W}C}_{W2}+R_{C}R_{T}C_{W2}+R_{T}R_{W}C_{W1}+{R_{T}R_{W}C}_{W2}$.

Combining [1] and [3] gives the governing equation for the CF and CPp models:

$$\alpha R_{C}C_{S}\frac{d^{3}P_{S}(t)}{{dt}^{3}}+(\alpha +\beta C_{S})\frac{d^{2}P_{S}(t)}{{dt}^{2}}+\gamma \frac{{dP}_{S}(t)}{dt}+P_{S}(t)={\alpha R}_{C}\frac{d^{2}F_{S}(t)}{{dt}^{2}}+\beta \frac{{dF}_{S}(t)}{dt}+{({R_{C}+R}_{T})F}_{S}(t)+{\bar{P}}_{E},$$ [4]

where $\gamma =R_{C}C_{S}+R_{T}C_{S}+R_{W}C_{W1}+R_{W}C_{W2}+R_{T}C_{W2}$.

R_S_ cannot be ignored if F_S_ is driven by gravity because the fluid reservoir is not an infinite impedance source but rather a height-adjustable pressure source. Flow across R_S_ is thus dependent on load of the eye. Reformulating [4] in terms of the pressure head P applied by the fluid reservoir gives the governing equation for the CPg model:

$$\alpha R_{C}C_{S}\frac{d^{3}P_{S}(t)}{{dt}^{3}}+(\frac{{\alpha R}_{S}+{\alpha R}_{C}+\beta {R_{S}C}_{S}}{R_{S}})\frac{d^{2}P_{S}(t)}{{dt}^{2}}+(\frac{\gamma R_{S}+\beta }{R_{S}})\frac{{dP}_{S}(t)}{dt}+(\frac{{{R_{S}+R}_{C}+R}_{T}}{R_{S}})P_{S}(t)=(\frac{{\alpha R}_{C}}{R_{S}})\frac{d^{2}P(t)}{{dt}^{2}}+(\frac{\beta }{R_{S}})\frac{dP(t)}{dt}\cdots +(\frac{{R_{C}+R}_{T}}{R_{S}})P(t)+{\bar{P}}_{E}.$$ [5]

A derivation of Eqs [1–5] is provided in S1 Appendix.

The governing equation of each model was numerically solved using a fourth-order Runge-Kutta algorithm and models/feedback loops were simulated and updated with a time resolution of 0.1 s in MATLAB (The MathWorks, Natick, MA). Table 1 lists parameter values used in the simulations. ${\bar{P}}_{E}$, R_T_, and C_W_ are based on reported data [17] and R_W_ on unpublished data [33] from adult Brown-Norway rats. C_W1_ and C_W2_ were set equal to 2C_W_ based on [27, 28]. R_C_ is based on a 33g needle used for rat eye cannulations [17]. C_S_ is based on published CPp and CPg systems [17, 21, 34, 35]. F_S_ is fixed in the CF model and dynamically regulated in CP models. The regulation is analog, instantaneous, and gravity driven for the CPg model, where flow is generated by the reservoir pressure head with gain of 1/R_S_. Gravity-driven systems with high and low R_S_ have been published [18, 24], so both were simulated and the models respectively named CPg1 and CPg2. Flow regulation is digital and slightly delayed for the CPp model. It was governed by the expression: *F*_*S*_(*t*) = *K*(*P*_*T*_−*P*_*S*_(*t*)), where P_T_ is the target pressure and K is feedback gain. K is based on the gain of published CPp systems [20, 21], scaled for the volume of rat eyes [36, 37].

**Table 1:** Parameter values of model simulations of eye perfusion techniques.

${\bar{P}}_{E}$	resting IOP	15	mmHg
*R*_*T*_	trabecular outflow resistance	43	mmHg min μl^-1^
*R*_*W*_	corneoscleral globe resistance	2.3	mmHg min μl^-1^
*C*_*W*_	corneoscleral globe compliance	0.09	μl mmHg^-1^
*R*_*C*_	cannula resistance	0.36	mmHg min μl^-1^
*C*_*S*_	system compliance	0.05	μl mmHg^-1^
*R*_*S*_	system resistance (CPg model)	0.6 or 10	mmHg min μl^-1^
*K*	feedback gain (CPp model)	7	μl min^-1^ mmHg^-1^

Parameters are for a rat eye cannulated with a 33g needle connected to published eye perfusion setups. Note that ${\bar{P}}_{E}$ embodies *F*_*A*_, *F*_*U*_, and *P*_*V*_ in Fig 1.

### Intrinsic dynamics of eye perfusion models

Model simulations were performed without noise to characterize the intrinsic response dynamics of the different eye perfusion techniques. F_S_ was increased for the CF model in steps of 0.1 μl/min, and P or P_T_ was respectively increased for the CPg and CPp models in steps of 5 mmHg. The response setting time to a step change in input was defined as the time for both P_S_ and F_S_ to reach 99% of their respective plateau levels calculated by the model. Settling times were determined for a range of feedback gains by logarithmically varying R_S_ from 0.01 to 100 mmHg·min·μl^-1^ in the CPg model and K from 0.01 to 10 μl·min^-1^·mmHg^-1^ in the CPp model.

### Simulating outflow facility experiments

Physiologically-realistic noise was added to the models to simulate outflow facility experiments performed on ex vivo and in vivo eyes. Three noise datasets were employed and are provided in S3 Appendix. Each contained 12 records of 1 or 2 hr duration. The first was constructed by passing white noise (sample rate: 10 Hz, SD: 0.04 mmHg) through a first-order lowpass filter (cutoff: 0.4 Hz) to produce noise records having similar statistical structure as published IOP data from enucleated mouse eyes [18] in terms of power spectrum and probability distribution. The other two datasets were constructed by extracting nonoverlapping segments of IOP data recorded at 20 Hz in anesthetized rats [38] and 1 Hz in conscious rats [39] and subtracting the mean IOP from each segment. Noise datasets were applied to models by varying P_S_ accordingly.

Response variability necessitated a means of determining steady-state pressure and flow levels. Model simulations explored the effectiveness of two criteria (window and ratio) used in ex vivo experiments [18, 21]. The window criterion defined steady state as the point when P_S_ varied over a 5-min period by <10% of input step size. The ratio criterion calculated F_S_/P_S_ and defined steady state when the slope of this ratio over a 5-min period was <0.1 nl·min^-2^·mmHg^-1^ for 1 min. These definitions were not suitable for in vivo noise, however, so P_S_ and F_S_ were recursively averaged over a fixed interval T to reduce data variability and the impact of data smoothing on model performance was evaluated over a range of T for each criterion. The filter width for which responses settled fastest with least variability across noise records was used to simulate in vivo experiments with that model. The heightened nonstationary noise in conscious animals inspired the creation of a novel method of steady-state determination, referred to as the fitting criterion. The method uses the expected step response dynamics of the eye and perfusion system to formulate noise-free predictions of pressure $P_{S}^{*}$ and flow $F_{S}^{*}$. For rat eyes, exponential response dynamics may be expected from Fig 1 and R_T_ » R_W_ (Table 1) to a first-order approximation. Nonlinear regression was then performed at 1s increments on accumulated P_S_ and F_S_ data using the fitting functions: $P_{S}^{*}=A(1-e^{-t/\tau })+B$ and $F_{S}^{*}=Ce^{-t/\tau }+D$, where A, B, C, and D are scaling and offset parameters and τ is response time constant. The recursive regression algorithm commenced 2 min after step onset and continued until steady state was reached using the window and ratio criteria as well as a fitting criterion, which was defined as $P_{S}^{*}$ and $F_{S}^{*}$ respectively deviating <0.5 mmHg and 0.05 μl·min^-1^ over a 2-min period that is more than 6·τ after step onset.

Facility experiments were simulated for each noise record by increasing F_S_ in steps of 0.1 μl/min for the CF model and P or P_T_ in steps of 5 mmHg for the CPg and CPp models. Steady-state P_E_ and F_E_ were determined for each step, and outflow facility was estimated via linear regression of pressure-flow data. Facility estimates across all records in a noise dataset were statistically compared between models and steady-state criteria and against the known outflow facility of the model eye (1/R_T_).

### Statistical analysis

The robustness of outflow facility estimates was quantified by Pearson coefficients (R) via linear correlation of simulated pressure-flow data. Facility estimates and experiment durations were compared between models and steady-state criteria and differences were statistically assessed across noise records at an α level of 0.05 using SigmaPlot software (Systat Inc, San Jose, CA, USA). Most datasets passed a Shapiro-Wilk normality test and Levene test for equality of variance and were subjected to a one-way repeated measures (noise records) ANOVA test followed by a Holm-Sidak test for multiple paired (models or criteria) comparisons, with results given as mean ± standard deviation. Those that did not pass were subjected to a one-way repeated measures ANOVA on ranks test followed by a Tukey test for multiple paired comparisons, with results given as median with lower and upper quartiles in brackets.

## Results

### Step response of eye perfusion models

**Fig 2:**
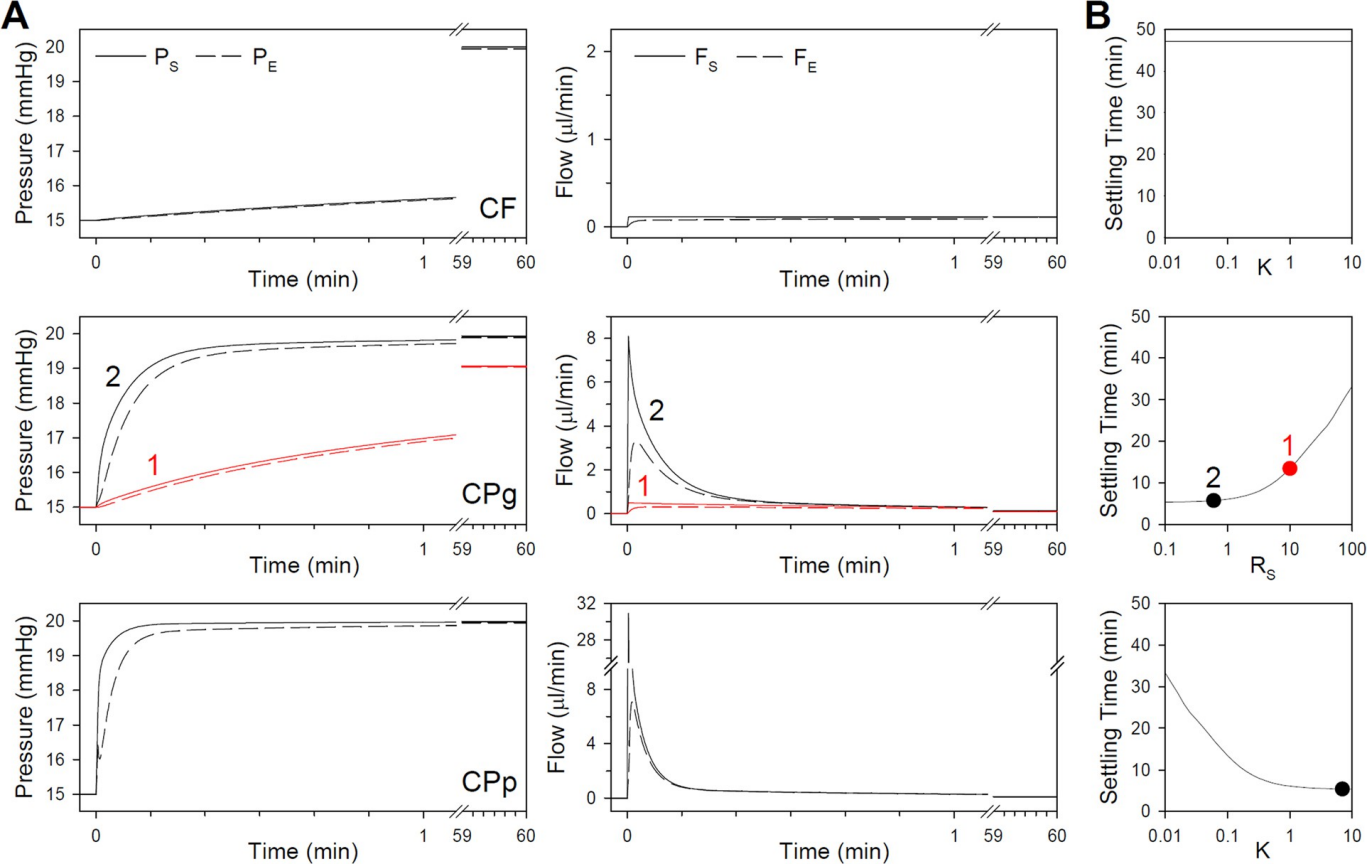
Intrinsic dynamics of simulated eye perfusion techniques. (A) Pressure (left) and flow (right) responses of the CF model (top) to a step change in F_S_, the CPg model (middle) to a step change in P (CPg1: red, CPg2: black), and the CPp model (bottom) to a step change in P_T_. Solid lines give P_S_ and F_S_, dashed lines give P_E_ and F_E_. (B) Time for pressure and flow to reach 99% of plateau level versus model feedback gain. Symbols indicate the gain and setting time of the CP models in A.

The intrinsic dynamics of common eye perfusion techniques were characterized with noise-free model simulations. Fig 2A shows responses of the CF model to a step increase in flow to 0.1 μl/min and CP models to a step increase in pressure to 20 mmHg. Several features of the pressure and flow traces may be noted. First, it can be seen that P_E_ tracks P_S_ closely but differs a small amount due to the pressure drop across R_C_. The difference is greatest for CP models at step onset when F_S_ is very large, and it becomes negligible in steady state for all models. Second, F_E_ and F_S_ differ for all models at step onset as flow is diverted to fill C_S_, but afterwards they also track closely out to steady state. Third, the CF model is comparatively slow. P_E_ took 47.2 min to reach 99% of the plateau level, yet only 5.4 and 5.8 min for the CPp and CPg2 models. The latter two settle quickly because their high gain initially drives F_S_ well above the plateau level, which accelerates filling of system and eye compliances. The CPg1 model has lower gain and therefore exhibits intermediate settling time (13.5 min). Fourth, P_S_ falls short of 20 mmHg for the CPg1 model due to the drop in applied pressure across the high R_S_. It also falls short for the CPg2 and CPp models by a much lesser extent that is barely visible owing to their higher gain. The shortfall would not have much experimental significance because outflow facility measurements depend on steady-state P_S_ or P_E_ and not on P. Fifth, response dynamics are more complex for the CPg2 and CPp models. P_S_ and P_E_ shoot toward near steady-state level as gravity or the pump inject fluid at high rate to reduce the large initial setpoint error. The jump in pressure coincides with a buildup in F_E_ and rapid fall in F_S_, after which they both decay exponentially to the plateau level with similar time constant. The complex onset dynamics are attributed to filling C_S_ at step onset, which rapidly builds a pressure gradient across R_C_ that drives fluid volume into the eye and gradually fills C_W1_ and C_W2._ The differential dynamics of the eye and perfusion system are less apparent for the CPg1 model since it has low gain and does not charge C_S_ nearly as fast. Sixth, steady-state flow is nearly equal for all models. Tiny discrepancies reflect differences in steady-state P_S_ that drive F_S_ in CP models. Fig 2B shows how settling time depends on feedback gain. The CF model exhibits no dependence since F_S_ is constant, but settling time grows progressively faster with logarithmic changes in gain for the CP models. Important to note is a minimum bound on data collection time, which is limited by the viscoelastic properties of the eye. The CPg2 and CPp models hit that minimum for rat eyes. Further reducing R_S_ or raising K would have little impact on their respective settling time. This also means that lowering K in the CPp model to 0.1 would yield step responses with identical dynamics as the CPg1 model.

### Enucleated eye simulations

**Fig 3:**
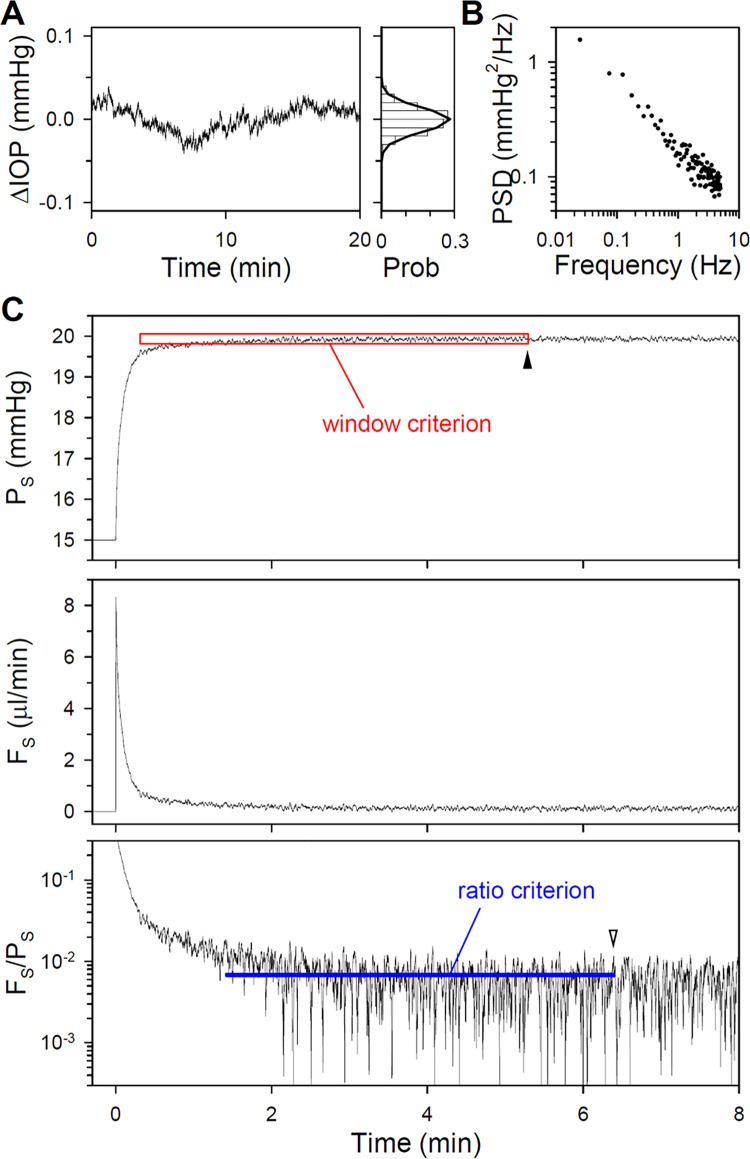
Analysis and simulation of enucleated eye noise. (A) Left, IOP data published by [18] for an ex vivo mouse eye [P15CL-01]. Right, histogram of IOP fluctuations. Thick line is a Gaussian fit of the probability distribution. (B) Average power spectrum of published IOP data from 12 ex vivo mouse eyes [18]. PSD: power spectral density. (C) Response of the CPg2 model to a noisy pressure step having statistics matched to ex vivo eyes. Red box indicates the window criterion for steady state, which is the 5-min running window wherein P_S_ must vary <0.5 mmHg. Blue line indicates the ratio criterion for steady state, which is the last 5-min running interval wherein the slope of F_S_/P_S_ must remain <0.1 nl·min^-2^·mmHg^-1^. Filled and unfilled arrowheads indicate the model settling time for the noise record in A for the window and ratio criterion, respectively.

Noise was added to the models to simulate physiological experiments. Noise statistics were based first on published data from enucleated mouse eyes [18]. Fig 3A plots an IOP record for one eye of that study sampled at 10Hz. Analysis of the dataset indicates that ex vivo fluctuations can be described by a normal distribution with SD of 0.04 mmHg. Fig 3B shows that noise power also decreased systematically with frequency. Noise records of any required length were created with these properties. Fig 3C shows the response of the CPg2 model to a noisy pressure step. Since the response fluctuates stochastically, steady-state pressure and flow levels were determined using different criteria that have been used experimentally. The time of steady-state detection for both the window criterion (5.3 min) and ratio criterion (6.4 min) was comparable for this noise record to the intrinsic noise-free settling time of the model (Fig 2B).

**Fig 4:**
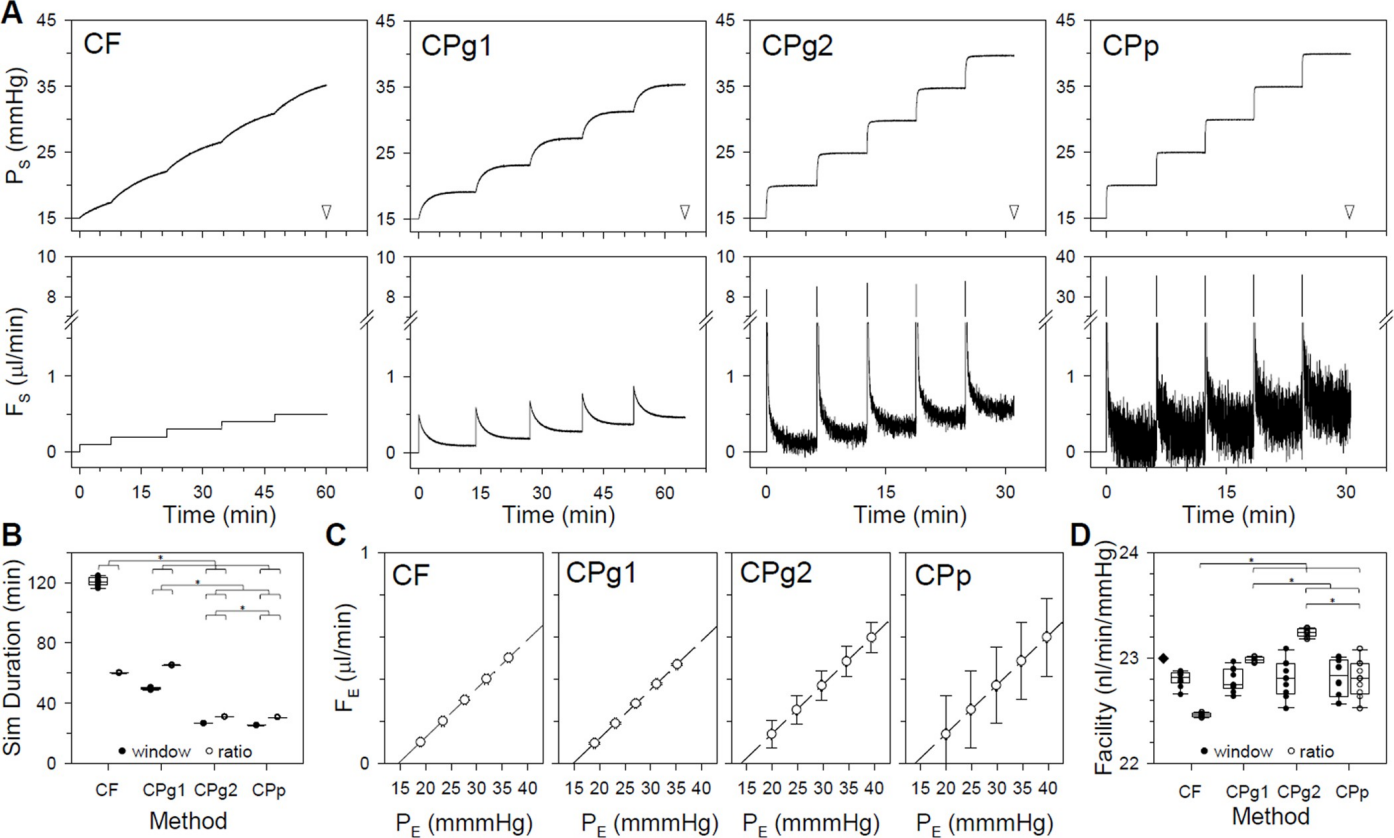
Simulation of facility experiments on enucleated eyes. (A) Pressure (top) and flow (bottom) responses of CF and CP models with ex vivo noise to a series of 0.1 μl/min steps in flow or 5-mmHg steps in pressure, respectively. Successive steps were initiated using the ratio criterion. Arrowheads indicate total duration of the simulated experiment. Note that the high gain of the CPp model necessitated a change in flow axis. (B) Summary of facility experiment durations across 12 ex vivo noise simulations using the window and ratio criteria (filled and unfilled symbols). (C) Steady-state P_E_ and F_E_ for the step response series in A. Dashed line is a linear regression fit (R>0.99 and slope = 23 nl·min^-1^·mmHg^-1^ for all models). (D) Summary of outflow facility estimates across noise simulations for the various models using the window and ratio criteria (filled and unfilled symbols). Diamond indicates the actual facility of the simulated rat eye. Error bars give standard deviation. Box and whiskers give 10, 25, 50, 75, and 90 percentiles. Asterisks indicate statistical differences between denoted groups.

Outflow facility measurements were simulated by adding ex vivo noise to the models and increasing pressure or flow in sequential steps. Fig 4A plots the CF and CP model responses to a step series triggered using the ratio criterion. At resting IOP levels and above, P_S_ and F_S_ approximate well the pressure and flow traces of enucleated mouse eyes that have been observed with the different perfusion systems [18, 24]. It may be noted that flow noise is much greater for the CPg2 and CPp models owing to their larger gain. Yet, Fig 4B shows that simulated facility experiments take the least time for these models. It also shows that simulation duration is significantly shorter using the window criterion for all but the CF model. Experiment duration statistics are summarized for ex vivo noise in Table 2 (p<0.05 between CPg2 and CPp, p<0.001 for all other paired comparisons). Fig 4C plots steady-state P_E_ and F_E_ of all models for the ratio criterion. The slope of the regression line estimated an outflow facility of 0.023 μl·min^-1^·mmHg^-1^ in every case, which equates to an outflow resistance of 43 mmHg·min·μl^-1^ that exactly matches R_T_ of the simulated rat eye (Table 1). Fig 4D shows that model facility estimates are indistinguishable, for the most part, regardless of steady-state criterion. Facility statistics are summarized for ex vivo noise in Table 2 (p<0.05 for all model comparisons using ratio criterion only). The simulations confirm that CF and CP perfusion techniques would both accurately measure the outflow facility of enucleated eyes under the conditions simulated.

**Table 2:** Statistics of outflow facility simulations with ex-vivo noise.

Model	Experiment Duration (min)		Facility Estimates (nl/min/mmHg)	
	Window Criterion	Ratio Criterion	Window Criterion	Ratio Criterion
**CF**	120.8 ± 2.8	60.2 ± 0.2	22.80 ± 0.07	22.46 ± 0.02
**CPg1**	49.7 ± 0.7	65.2 ± 0.2	22.80 ± 0.17	22.98 ± 0.02
**CPg2**	26.6 ± 0.1	31.0*	22.78 ± 0.11	23.24 ± 0.04
**CPp**	25.3 ± 0.1	30.6*	22.81 ± 0.18	22.80 ± 0.17

### Anesthetized animal simulations

**Fig 5:**
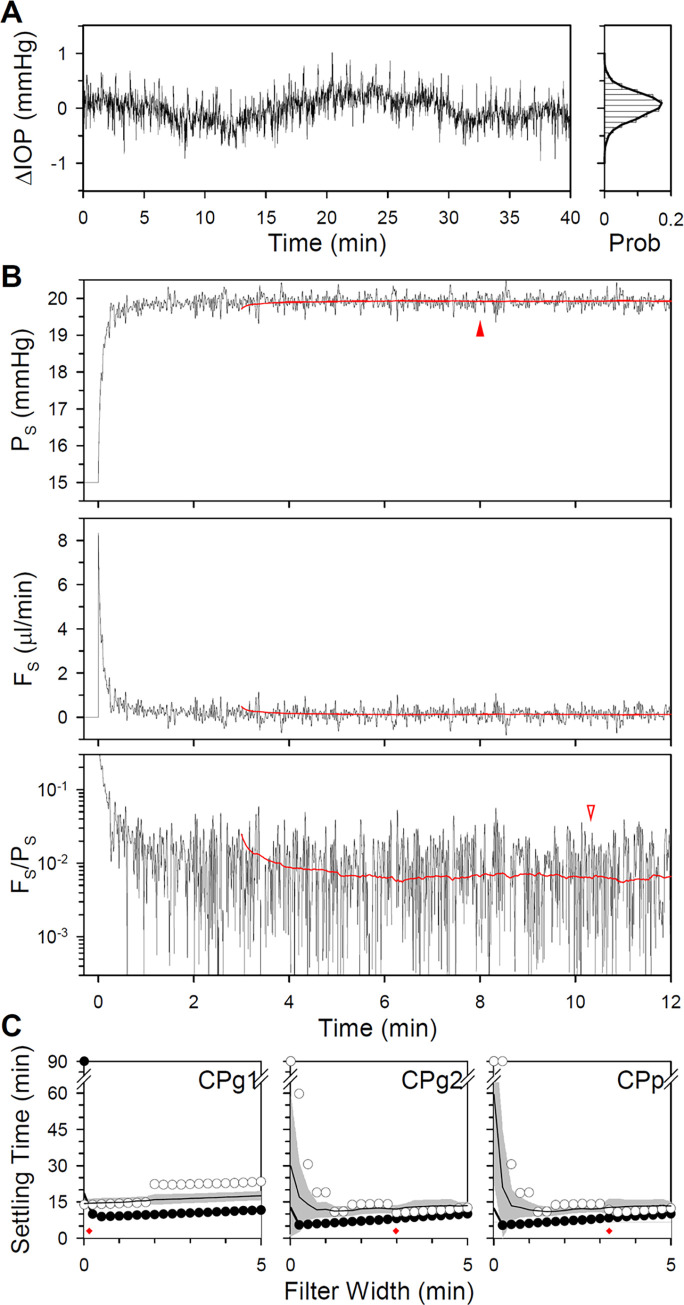
Analysis and simulation of anesthetized animal noise. (A) Left, IOP data of an anesthetized rat collected previously by the lab [17]. Right, histogram of IOP fluctuations. Thick line is a Gaussian fit of the probability distribution. (B) Raw (black) and filtered (red) step response of the CPg2 model for the noise record in A. Filtered responses are a 3-min moving average of model P_S_ and F_S_ data. Filled and unfilled arrowheads indicate the settling time using window and ratio criteria on filtered responses, respectively. (C) Effect of filter width on settling time of different CP models for the noise record in A using window and ratio criteria (filled and unfilled black circles). Mean and standard deviation of model settling times across 12 anesthetized noise records are indicated by lines (thick: window, thin: ratio) and shaded areas (dark: window, light: ratio), respectively. Diamonds mark the filter width with the fastest settling time and least variability (CPg1: 2 s, CPg2: 3 min, CPp: 3.25 min).

Experiments on anesthetized animals were simulated next. Only the CP models were explored since the CF model is rather slow. Fig 5A plots IOP data recorded previously by our lab from a ketamine-anesthetized rat [17]. Noise statistics can be approximated by a normal distribution, as for ex vivo eyes, but IOP is more variable (SD: 0.23 mmHg) and can exhibit sporadic transient fluctuations. Fig 5B shows pressure and flow responses of the CPg2 model to a step change in P_S_ for the illustrated noise record. Steady state could not be determined using ex vivo window or ratio criteria due to in vivo noise variability, so model output was smoothed with a moving average filter to reduce pressure and flow fluctuations to criterion levels. A 3-min filter width yielded a settling time of 8.0 and 10.4 min for this record using the window and ratio criterion, respectively, which is close to that for ex vivo noise. Fig 5C summarizes the dependence of response settling time on filter width across 12 records of anesthetized noise. Shorter widths generally translate to faster settling times as less data is required for processing. However, responses become noisier so settling time grows increasingly erratic from trial to trial, especially for the ratio criterion. A best filter width was therefore identified that produces the fastest settling time with the least variability to anesthetized noise for each model (CPg1: 2 s, CPg2: 3 min, CPp: 3.25 min).

**Fig 6:**
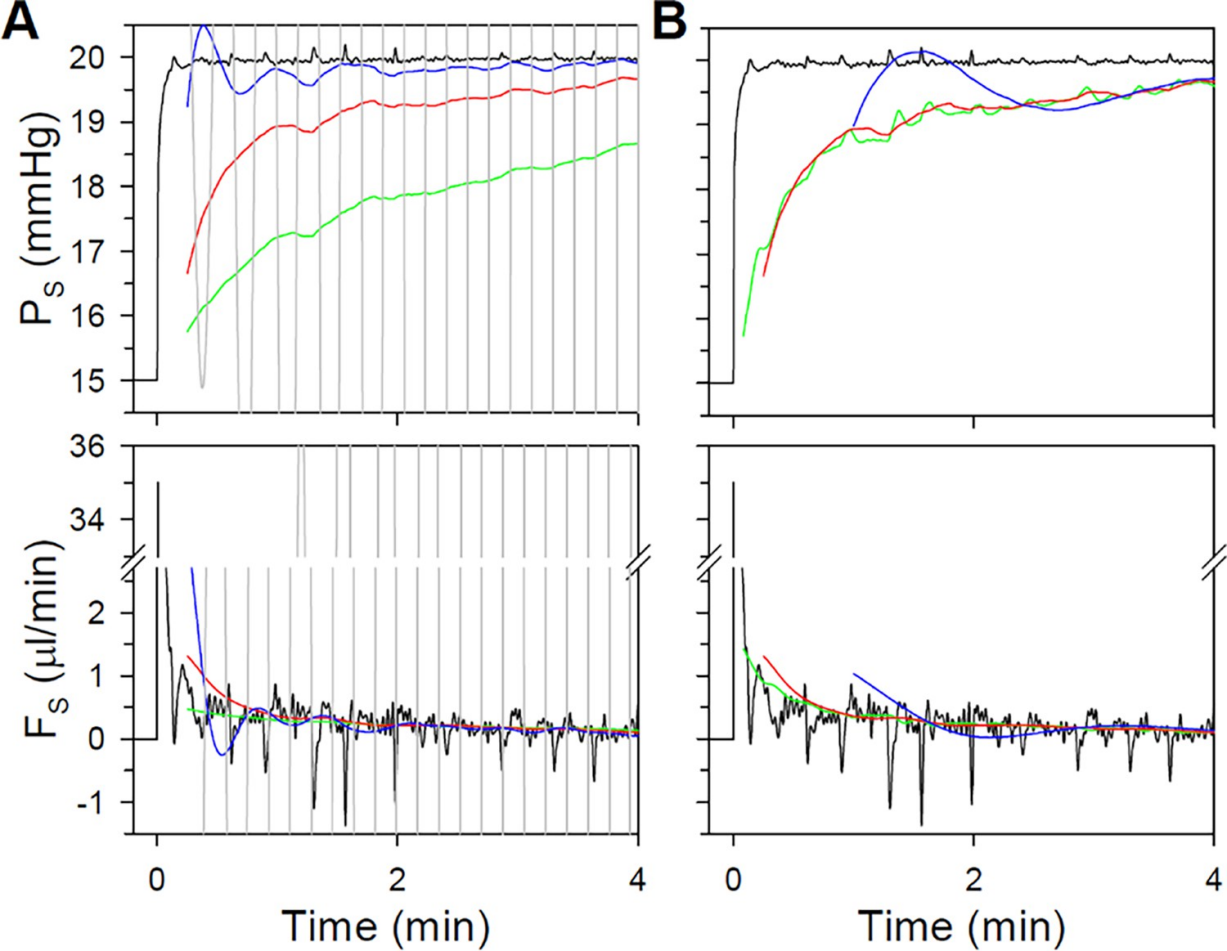
Tradeoff of feedback gain and noise filtering on flow regulation. (A) Effect of smoothing the feedback signal with a 15-s moving average filter on the pressure (top) and flow (bottom) responses of the CPp model to a step change in P_T_ for a feedback gain of 7 (gray), 1 (red), 0.3 (blue), and 0.1 (green). (B) Effect of smoothing the feedback signal with a 5-s (green), 15-s (red), and 60-s (blue) moving average filter on pressure and flow responses of the CPp model with a feedback gain of 0.3. Black traces give step responses of the CPp model without feedback filtering.

The CPp technique offers the unique option of regulating flow in real time with filtered IOP data since pump rate is computer controlled. Fig 6A shows that this option can greatly reduce flow noise but also cause response oscillations. The undesirable behavior reflects a temporal mismatch of system dynamics with the feedback delay introduced by filtering. Oscillatory instabilities can be attenuated by lowering feedback gain, which reduces noise in both P_S_ and F_S_ at the expense of response speed and essentially turns the CPp model into a CPg1-like model. Fig 6B shows the effect of filter width on a low-gain CPp model. Lengthening filter width decreases noise in P_S_ and F_S_ up to a specific point beyond which responses oscillate and become increasingly unstable. It was found that a feedback gain of 0.3 and filter width of 15 s provide a good tradeoff between speed and noise reduction, and this model is referred to as CPpx.

**Fig 7:**
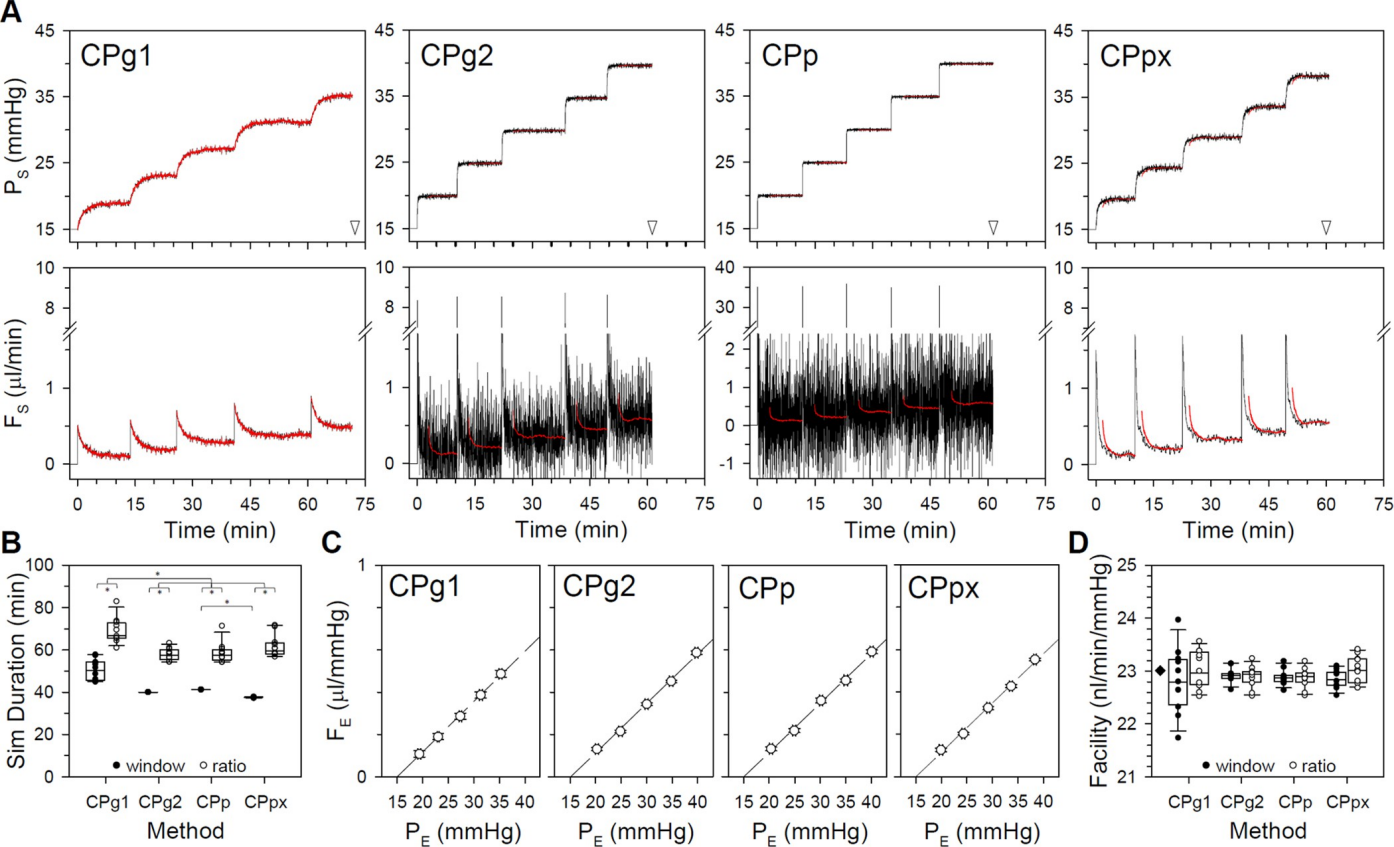
Simulation of facility experiments on anesthetized animals. (A) Raw (black) and filtered (red) pressure (top) and flow (bottom) responses of CP models to a series of 5-mmHg steps in P_T_ contaminated with IOP noise recorded from an anesthetized rat. Successive steps were initiated using the ratio criterion, and responses were filtered with a moving average window that provided best model performance for anesthetized noise according to Fig 5C. Arrowheads indicate the experiment duration for the noise record in Fig 5A. Note that the high gain of the CPp model necessitated a change in flow axis. (B) Summary of facility experiment durations across 12 anesthetized noise simulations using the window and ratio criteria (filled and unfilled symbols). (C) Steady-state P_E_ and F_E_ for the filtered step response series in A. Dashed line is a linear regression fit (R>0.99 and slope = 23 nl·min^-1^·mmHg^-1^ for all models). (D) Summary of outflow facility estimates across 12 anesthetized noise records for the various models using the window and ratio criteria (filled and unfilled symbols). Diamond indicates actual facility of the simulated rat eye. Error bars give standard deviation. Box and whiskers give 10, 25, 50, 75, and 90 percentiles. Asterisks indicate statistical differences between denoted groups.

Outflow facility experiments in anesthetized rats were simulated for each CP model using the best filter width to reduce data variability and minimize setting times. Fig 7A plots raw and filtered pressure and flow responses to a series of noisy pressure steps triggered by the ratio criterion. It can be seen that the CPg1 model took the most time to complete the experiment and that the CPg2, CPp, and CPpx models took about the same time, even though the CPpx model has low gain like the CPg1 model. It can also be seen that CPpx responses are much less noisy than CPg2 and CPp responses, illustrating the need for data filtering as in vivo noise would otherwise obscure F_S_ responses to the step series. Fig 7B summarizes experiment duration across 12 anesthetized noise records for each model and steady-state criterion. Simulation duration was consistently similar for the CPg2, CPp, and CPpx models and longer for the CPg1 model. It was also significantly shorter for all models by ~20 min for the window criterion. Experiment duration statistics for in vivo anesthetized noise are provided in Table 3 (p<0.01 for all criterion comparisons, for all model comparisons with CPg1, and between CPp and CPpx for window criterion). Fig 7C plots steady-state P_E_ and F_E_ of filtered step responses using the ratio criterion. All models correctly estimate an outflow facility of 23 nl·min^-1^·mmHg^-1^ for this noise record. Fig 7D shows that facility estimates across noise records are indistinguishable between models for both window and ratio criteria. Hence, if IOP noise is adequately filtered below criterion levels, all CP techniques would measure outflow facility in anesthetized animals accurately. Facility statistics are summarized for in vivo anesthetized noise in Table 3.

**Table 3:** Statistics of outflow facility simulations with in-vivo anesthetized noise.

Model	Experiment Duration (min)		Facility Estimates (nl/min/mmHg)	
	Window Criterion	Ratio Criterion	Window Criterion	Ratio Criterion
**CPg1**	50 ± 5	69 ± 6	22.8 ± 0.6	23.0 ± 0.3
**CPg2**	40*	58 ± 3	22.9 ± 0.1	22.9 ± 0.2
**CPp**	41*	58 ± 5	22.9 ± 0.1	22.9 ± 0.2
**CPpx**	38*	61 ± 5	22.8 ± 0.2	23.0 ± 0.3

*steady state was detected immediately after filter initiation for every noise record

### Conscious animal simulations

**Fig 8:**
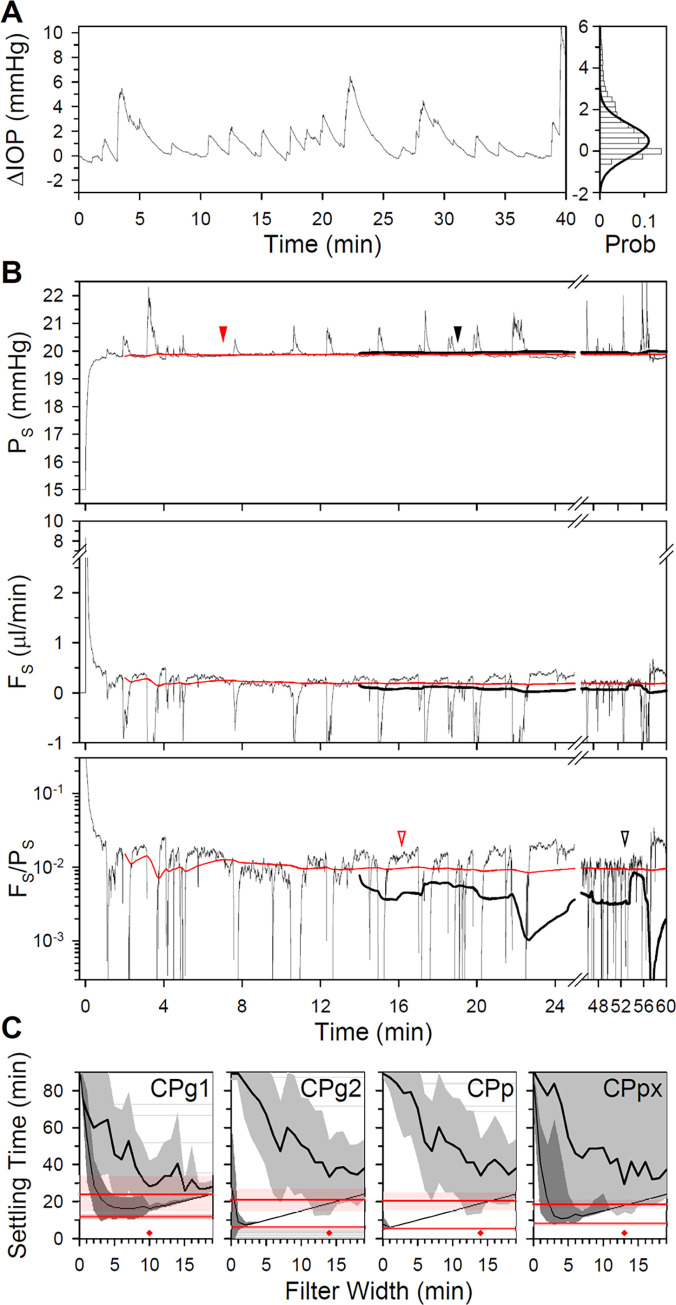
Analysis and simulation of conscious animal noise. (A) Left, IOP data of an awake free-moving rat collected previously by the lab [40]. Right, histogram of IOP fluctuations. Thick line is a Gaussian fit of the probability distribution. (B) Unfiltered and 14-min lowpass filtered (thin and thick black lines) step responses of the CPg2 model for the noise record in A. Unfiltered step responses were processed by a recursive regression method based on the eye model in Fig 1 to yield fitted pressure and flow responses (red lines). Filled and unfilled arrowheads indicate settling time of the filtered (black) and fitted (red) responses for the window (19.0 and 7.0 min) and ratio (52.8 and 16.2 min) criteria, respectively. (C) Effect of filter width on average settling time of the various CP models across 12 conscious noise records for the window (thin black line) and ratio (thick black line) criteria. Thin and thick red lines indicate the mean settling time of recursively-fitted model responses for the window and ratio criteria, respectively. Shaded areas give the standard deviation of filtered and fitted response settling times using window (dark gray and dark pink) and ratio (light gray and light pink) criteria. Diamonds mark the filter width with the fastest settling time and least variability (CPg1: 10 min, CPg2: 14 min, CPp: 14 min, CPpx: 13 min).

Lastly, experiments on awake free-moving animals were simulated. Fig 8A plots IOP data collected previously by our lab from a conscious rat [40]. A distinctive feature is the large erratic bumps in IOP, which produce a noise distribution that is positively skewed. The IOP bumps pose a major challenge for steady-state analysis. The best filter width for anesthetized noise was inadequate for conscious noise as simulations did not generally converge. Longer widths were needed to detect steady state within the 90 min of allotted time. Fig 8B shows pressure and flow responses of the CPg2 model to a step change in P_S_ for the illustrated noise record using a 14-min filter width. It can be seen that plateau levels are still tricky to identify because responses like the F_S_/P_S_ ratio can still undulate markedly after significant noise filtering. Hence, settling times were heavily influenced by the random occurrence of bumps, especially for the ratio criterion, and a novel method of steady-state identification was devised. The method generates predicted pressure and flow responses by recursively fitting raw data to an exponential function based on the eye model of Fig 1. Fig 8B shows that the recursive regression method greatly reduces fluctuations in P_S_ and F_S_, which greatly accelerates steady-state detection. Settling time of the fitted response to this noise record was 7.0 and 16.2 min for the window and ratio criteria compared to 19.0 and 52.8 min for the best filtered response. It is fairly insensitive to IOP bumps because the fitted response grows progressively robust as the regression accumulates more data. Fig 8C compares settling times of the various CP models across 12 conscious noise records. In every case, the recursive regression method outperforms filtering irrespective of filter width or steady-state criterion. This is especially true for the low-gain CPg1 and CPpx models, for which experiment duration was rather irregular for both criteria. A best filter width was therefore identified that produces the fastest settling time with the least variability to conscious noise for each model (CPg1: 10 min, CPg2: 14 min, CPp: 14 min, CPpx: 13 min).

**Fig 9:**
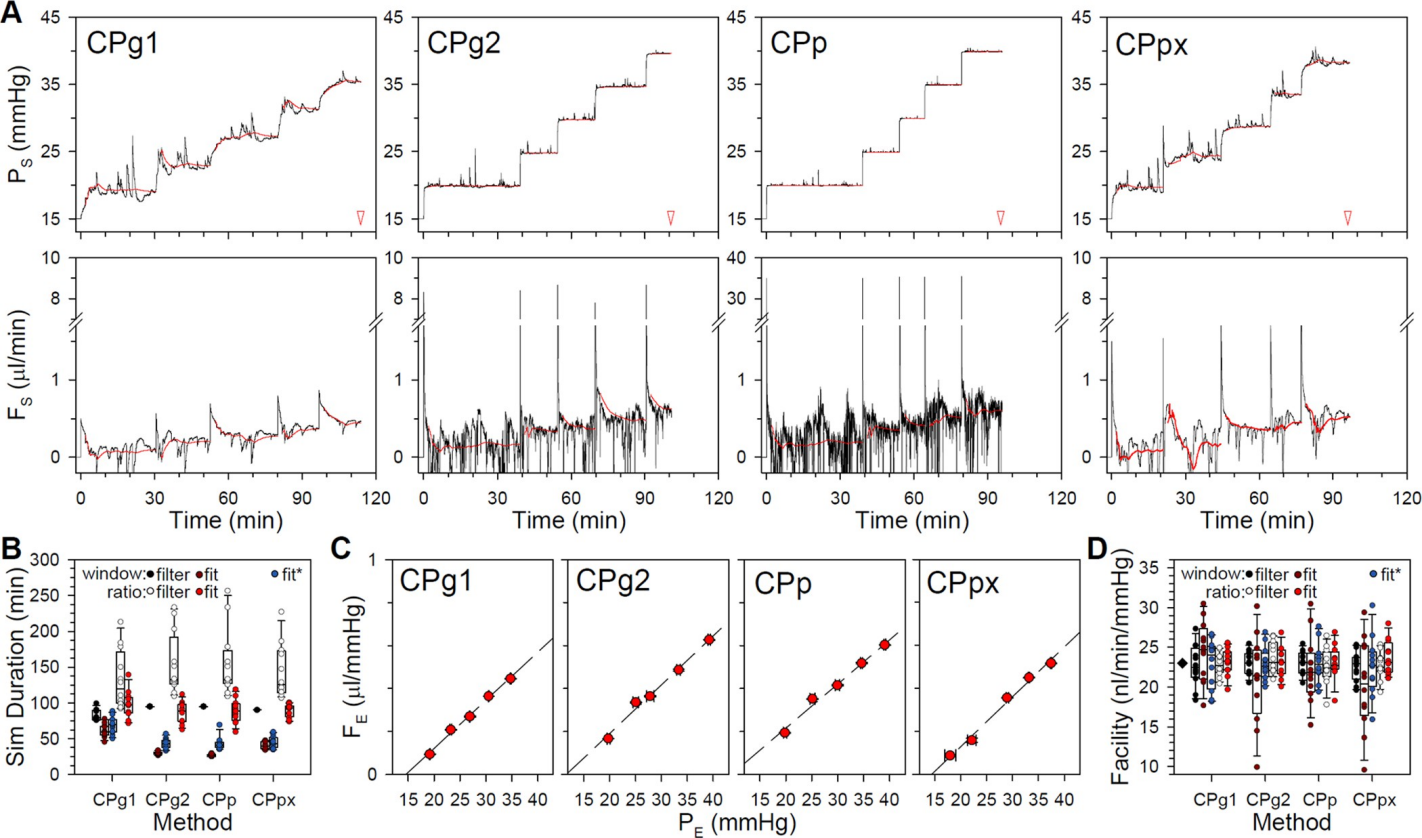
Simulation of facility experiments on conscious animals. (A) Raw (black) and regression-fitted (red) pressure (top) and flow (bottom) responses of CP models to a series of 5-mmHg steps in P_T_ contaminated with IOP noise recorded from a conscious rat. Successive steps were initiated using the ratio criterion, and responses were filtered with a moving average window that provided best model performance for conscious noise according to Fig 8C. Arrowheads indicate the experiment duration for the noise record in Fig 8A. Note that the high gain of the CPp model necessitated a change in flow axis. (B) Summary of facility experiment durations across 12 records of conscious noise using the window and ratio criteria on fitted (dark and light red symbols) and best-filtered (black and while symbols) responses and using the novel fitting criterion on fitted responses (blue symbols). (C) Steady-state P_E_ and F_E_ for the fitted step response series in A. Solid line is a linear regression fit (R>0.98 for all models, slope = 23 nl·min^-1^·mmHg^-1^ for CPg1, CPg2, and CPpx models and 21 nl·min^-1^·mmHg^-1^ for CPp model). (D) Summary of outflow facility estimates across 12 conscious noise records for the different models using the window and ratio criteria on fitted (dark and light red symbols) and best-filtered (black and while symbols) responses and the novel fitting criterion on fitted responses (blue symbols). Diamond indicates actual facility of the simulated rat eye. Error bars give standard deviation. Box and whiskers give 10, 25, 50, 75, and 90 percentiles.

Outflow facility experiments in conscious rats were then simulated with the various CP models. Fig 9A plots raw and regression-fitted pressure and flow responses to a series of noisy pressure steps triggered using the ratio criterion. The fitting algorithm is able to reliably identify steady state, but slow sporadic response fluctuations noticeably lengthen experiment duration compared to anesthetized noise. CPg2, CPp, and CPpx simulations are similar again in duration for this noise record, and CPg1 simulations are also ~20 min longer. Fig 9B summarizes simulation duration of fitted and best-filtered model responses across conscious noise records. In general, the duration is much longer and more variable than for enucleated and anesthetized noise (Figs 4B and 7B). All model simulations are also significantly shorter in duration for fitted than for best-filtered responses irrespective of steady-state criterion. Experiment duration statistics for in vivo conscious noise are provided in Table 4 (p<0.01 for all comparisons of fitted and best-filtered responses). Fig 9C plots steady-state P_E_ and F_E_ of the illustrated step responses. Despite the IOP bumps, outflow facility is correctly estimated by all models for this noise record except perhaps by the CPp model. Fig 9D shows that facility estimates vary noticeably across conscious noise records but still distribute around 23 nl·min^-1^·mmHg^-1^ for all models. Estimates are indistinguishable between fitted and best-filtered responses as well as between window and ratio criteria, but fitted responses appear more variable and off-center for the window criterion. A fitting criterion was thereby created that defined steady state when a specified number of regression-estimated time constants have elapsed since step onset. Fig 9B and 9D show that the fitting criterion estimates facility with similar precision and reliability as the window and ratio criteria while maintaining the short experiment duration. Facility statistics are summarized for in vivo conscious noise in Table 4. Hence, all CP techniques can measure outflow facility in conscious animals if noise is filtered but experiments will take longer and need repeating due to sporadic IOP bumps to estimate facility accurately. The fitting method in combination with the fitting criterion achieves the same estimation accuracy while greatly speeding data collection.

**Table 4:** Statistics of outflow facility simulations with in-vivo conscious noise.

	**Experiment Duration (min)**				
	Best Lowpass Filter		Fitting Filter		
	Window	Ratio	Window	Ratio	Fitting
**CPg1**	79 [77, 90]	131 ± 41	62 ± 10	96 ± 13	69 ± 12
**CPg2**	95*	132 [123, 208]	30 ± 2	91 ± 18	43 ± 7
**CPp**	95*	132 [123, 169]	26 [25, 28]	88 ± 15	41 [38, 45]
**CPpx**	90*	147 ± 38	40 ± 4	93 [25, 28]	45 ± 8
	**Facility Estimates (nl/min/mmHg)**				
	Best Lowpass Filter		Fitting Filter		
	Window	Ratio	Window	Ratio	Fitting
**CPg1**	22.7 ± 2.7	22.7 ± 1.4	24.0 ± 3.6	23.0 ± 1.7	22.9 ± 3.0
**CPg2**	22.9 ± 1.2	23.4 ± 1.5	22.5 ± 4.8	23.2 ± 1.9	22.9 ± 2.1
**CPp**	22.9 ± 1.2	22.5 ± 2.0	23.0 ± 6.4	22.9 ± 2.2	23.1 ± 2.4
**CPpx**	22.3 ± 1.7	23.3 ± 2.3	21.2 ± 5.3	23.4 ± 2.0	22.7 ± 4.2

*steady state was detected immediately after filter initiation for every noise record

**Fig 10:**
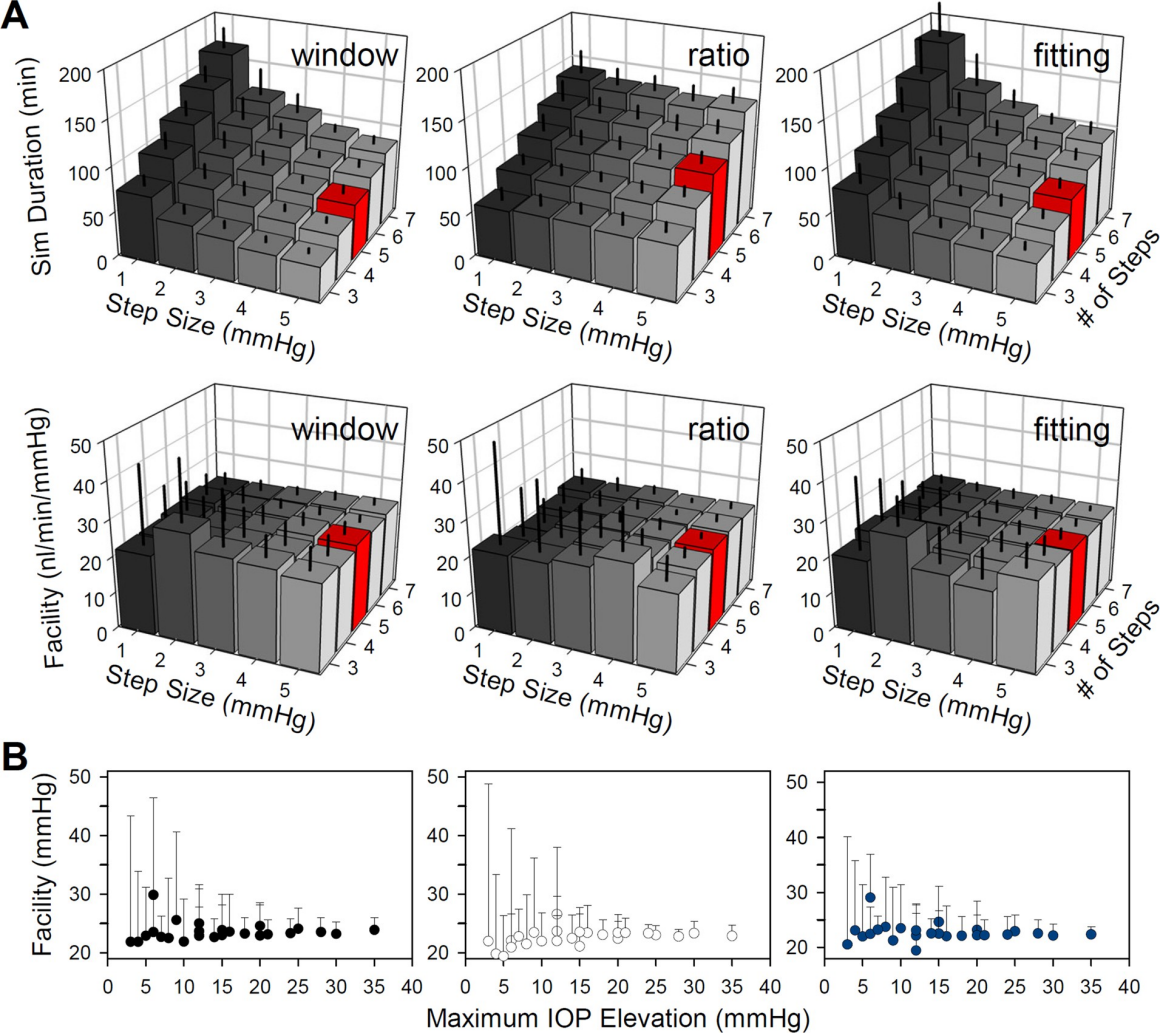
Optimizing facility experiment design. (A) Summary of simulated experiment durations (top) and facility estimates (bottom) of the CPg1 model across conscious noise records using the window (left), ratio (middle), and fitting (right) criteria for pressure step series ranging in step size and number. Red boxes indicate the baseline series of 5 steps of 5 mmHg. (B) Dependence of facility data in A on the maximum IOP elevation of the step series. Error bars give standard deviation.

The veracity of simulation results under conscious noise conditions was examined for an array of pressure inputs. Fig 10A summarizes the impact of the number and size of steps on simulation duration and facility estimates for regression-fitted responses of the CPg1 model, which is more attractive for use in awake free-moving animals because it performs similar to the high-gain models with less noise and potential for instability (Figs 6–9). The pattern of dependence on step numbers and size is fundamentally the same for the CPg2 and CPp models (not shown). In general, smaller step sizes translate to greater response variability, experiment duration, and facility estimation error. More steps leads to longer experiments, of course, but it does not significantly impact accuracy of mean facility estimates across the set of experiments (i.e. noise records) for any of the steady-state criteria beyond 4 steps. It does though greatly reduce variability in facility estimates across experiments. More steps also leads to higher terminal IOP elevation, which may be deleterious to the eye. Fig 10B replots facility data against the product of step size and number. Model simulations indicate that elevating IOP more than 15 mmHg offers little-to-no improvement in estimation accuracy or error for any of the criteria.

## Discussion

Infusion-based techniques of measuring ocular fluid dynamics were simulated with a lumped parameter viscoelastic model to understand their relative strengths and limitations and to compare their performance under different noise conditions that are encountered experimentally. To make the simulations realistic, model structure and parameters were based on published CF, gravity-driven CP, and pump-driven CP eye perfusion systems, published aqueous outflow and IOP noise data obtained with these systems from rodent eyes, and published methods of data analysis for estimating eye outflow facility. Model simulations appeared qualitatively similar to pressure and flow responses of ex vivo and in vivo rodent eyes to step inputs and confirmed that all infusion techniques accurately measure outflow facility in the absence of noise in the presented model. The simulations showed that all techniques can also estimate facility quite well in the presence of highly non-stationary IOP noise if incoming data are suitably processed. Longer experiment times are needed, though, as heightened noise levels delay the detection of steady-state pressure and flow levels using published ratio and window criteria. This is undesirable for practical reasons and for data interpretation because circadian rhythms, physiological washout, and other factors could cause outflow properties to drift during hours-long facility measurements [35, 41]. A novel steady-state detection method was introduced to speed data collection, which recursively regresses incoming data to the viscoelastic model of the eye and perfusion system. The method was shown to markedly shorten the duration of simulated facility experiments without compromising accuracy and that further shortening can be achieved by optimizing input step size and number to experimental noise levels. The potential impact of repeated IOP perturbations on the eye could thereby be minimized. These findings can accelerate measurements of ocular fluid dynamics in anesthetized animals and facilitate round-the-clock monitoring of outflow facility in conscious animals [34].

### Simulation insights

There are numerous insights to glean from model simulations, and five are highlighted here. Firstly, CF techniques settle much more slowly than CP techniques for an equivalent step change in IOP and are therefore less experimentally appealing. CP techniques are faster because they allow for higher instantaneous flow rates that quicken IOP elevation to the target level. A possible concern with high-gain pump-driven (CPp) systems is that the instantaneous rate, after system compliance has been charged (>5 s), is around 10-fold greater than the steady-state rate (Fig 2). While the flow transient is brief (<1 min), it would cause IOP to increase over 50 mmHg if maintained and could perhaps initiate or potentiate glaucomatous processes. Long-term effects on ocular tissue should be considered when using a high-gain system for repeated facility measurement. A low-gain pump-driven (CPpx) system offers a potential compromise between speed and safety for purposes of chronic use. Secondly, pressure and flow responses to a step input reflect a combination of perfusion system and ocular fluid dynamics, the latter of which is much slower and thereby limits response settling time. The lower limit in absence of noise is around 1 min for rat eyes based on reported values of ocular compliance and outflow resistance (Fig 2). It would be much longer for animals with larger eyes or with addition of physiological noise. Yet, settling times under 2 s were reported using a CPp system on enucleated porcine eyes [21]. Model simulations suggest the surprisingly fast dynamics may have reflected the high feedback gain of the pump-driven system rather than the eye. IOP responses can appear to plateau in the first few seconds with a high-gain system but that does not necessarily mean flow responses have stabilized (Fig 3). High-gain systems can also exhibit ringing that could further obfuscate slower decaying components (Fig 6), leading to premature determination of steady-state response levels. The simulations draw attention to the importance of ensuring ocular fluid dynamics have fully settled to measure outflow facility accurately, especially when steady state is obscured by noise. Thirdly, pressure and flow are coupled in CP systems because of their feedback nature, meaning that IOP noise gets transferred into flow records and vice versa. Increasing feedback gain to speed step responses therefore comes with the cost of simultaneously amplifying flow noise (Figs 4 and 7). The noise amplification does not necessarily degrade the performance of high-gain systems based on CPg2 and CPp model simulations. It would, though, have important implications for system design. For example, to avoid saturated measurements from affecting filtered flow estimates, the flow transducer of a gravity-driven system would need a larger dynamic range, which would in turn decrease flow resolution. While, the feedback control of a pump-driven system would need to operate at high speed to prevent large erratic IOP swings from introducing feedback delays that can destabilize pump output. Fourthly, a series of five 5-mmHg pressure steps produces a rather robust facility estimate. IOP noise increased data variability and lengthened experiment duration, nonetheless facility estimates remained narrowly distributed about the correct value. Even for simulations of conscious noise, the coefficient-of-variation was below 10% irrespective of model, steady-state criterion, or noise-processing method (Fig 9D). The robustness is related to the broad (15–40 mmHg) range of pressure inputs, which necessitates higher noise levels for individual pressure-flow measurements to skew the regression slope. This is apparent from conscious noise simulations as IOP fluctuations were sufficiently large and irregular to generate noticeable scatter in facility estimates across noise records (Fig 9). Similar estimation accuracy can be achieved with fewer or smaller steps, but performance begins to suffer pointedly with conscious noise for series of less than 4 steps or steps of less than 3 mmHg (Fig 10). Fifth, the window criterion consistently outperformed the ratio criterion in terms of settling time. This translated to shorter facility experiments irrespective of IOP noise level. More surprising is that both criteria yielded similar facility results, even though the ratio criterion takes both flow and pressure into account. The surprising performance of the window criterion may be attributed, in part, to the relative strictness of published thresholds. For example, relaxing the ratio criterion by decreasing the time that the slope of the ratio must remain below threshold or by raising the threshold level would reduce settling time and experiment duration, with little apparent cost in facility measurement accuracy given the robustness of simulation results.

### Gravity-driven versus pump-driven perfusion systems

Model simulations make clear that gravity-driven and pump-driven CP systems are theoretically equivalent. Both generate pressure and flow traces with identical dynamics if matched in gain K. Feedback regulation is obvious for CPp systems since the pump is explicitly programmed to modulate flow rate in proportion to the difference between measured and target IOP. It is perhaps less apparent for CPg systems because feedback happens hydrodynamically, but flow rate is similarly proportional to the difference in IOP from the target level set by fluid reservoir height. The proportionality constant in this case is specified by the inverse of system resistance R_S_. Decreasing R_S_ thus has the same impact on response dynamics, in theory, as increasing K of CPp systems (Fig 2).

From a research perspective, there are important distinctions between gravity-driven and pump-driven systems worth noting. The most significant advantage of CPg systems is that flow control is analog, instantaneous, and automatic, which makes system design simple and ensures functional stability as noise intrinsic to pressure or flow sensors is not fed back into the system. In contrast, feedback control is digital for CPp systems, which imposes limits on flow update rate and temporal resolution of flow output in high-gain systems. It also introduces a feedback delay that could cause pump output of high-gain systems to overshoot or ring (Fig 6), which is experimentally unwelcome and could injure ocular tissue. Care should be exercised and max flow rate limited when using high-gain CPp systems, especially in anesthetized or conscious animals. Instability concerns can be mitigated by low-gain CPp systems at the expense of response speed. In addition, flow is smooth in CPg systems but pulsatile in CPp systems owing to the pump stepper motor. With proper design, pump pulsations can be filtered by system dynamics and their potential effects on CPp measurements can be mitigated. The most significant advantage of CPp systems is that K can be set to any value and that value can be easily modified. In contrast, the gain of CPg systems is a fixed property set by the highest impedance element, which is generally the flow transducer [18, 24]. This also means that gain is limited to R_S_ values available in the flow transducer marketplace. Another advantage is that flow is specified by pump infusion rate. It does not have to be measured with a flow transducer or manipulated through reservoir height adjustments like CPg systems. Only a pump and pressure sensors are needed, which are comparatively inexpensive and compact. In addition, data processing algorithms are straightforward to embed in CPp systems. Model simulations showed that lowpass filtering the feedback signal can reduce flow noise and speed data collection when IOP noise is random (Fig 7, CPpx). In short, CPg systems like iPerfusion cannot be beat for facility measurements on ex vivo eyes, while CPp systems become more attractive for use in anesthetized and conscious animals owing to their smaller portable footprint and lower potential cost.

Other forms of feedback regulation besides proportional control were investigated with the CPp model. They are not included for sake of brevity due to performance drawbacks. Integral control invariably introduced ringing and overshoot behavior that would raise IOP to undesirable levels, especially at higher setpoints or larger steps. Addition of derivative control to counteract the behavior led to amplification of noise, which is also undesirable. The extra noise could be combated with a sufficiently long filter but that introduced time lags which further complicated matters. In addition, the main reason for integral control is to eliminate target errors. Since pressure and flow are both recorded and reach a steady-state level, failure of the system to realize the target level is unimportant for outflow facility measurements.

A potential hybrid design is suggested by model simulations that combines strengths of gravity- and pump-driven systems. Response dynamics of CPg systems are presently limited by flow transducer impedance, which sets the rate that gravity delivers flow and therefore the time required to charge system and eye compliances to steady-state levels. However, higher flow rates can still be achieved, even though transducer impedance is fixed, by increasing the pressure head. A motor could initially elevate the reservoir above target height, generating higher than normal flow, and proportionately lower the reservoir as measured IOP approaches the target level. Such a feedback-controlled adjustment of reservoir height would impart adjustable gain akin to K of CPp systems, with much less risk of instability since pressure equilibrates instantly through gravity. A motor-driven CPg system would, in principle, shorten facility experiment duration beyond that attainable with a purely gravity- or pump-driven system alone, especially in enucleated eyes given their low noise levels. It would fall subject to similar limitations on temporal resolution as CPp systems, especially given the larger inertial components and movements involved.

### Determining steady-state response levels

Model simulations indicate that steady-state identification is comparable in importance to CP system performance as gain. An ideal steady-state criterion is insensitive to noise so that little-to-no modification is needed between animals and experiments. The window criterion was most difficult to properly tune. A wide window would either trigger early as IOP responses started flattening out or erratically late when noise quiesced for a requisite period, while a narrow window would oftentimes not trigger with in vivo noise (not shown). The published ex vivo window was therefore adopted and applied to all simulations for consistency. The ratio criterion has most intrinsic appeal since it is a pseudo-measure of facility. However, the gain of CP systems was found to amplify flow noise, which delayed triggering and lengthened settling time. Increasing gain in order to accelerate response dynamics thereby produced more variable facility estimates (Figs 4 and 7). Noise filtering helped shorten experiment duration but not enough for the ratio criterion to outcompete the window criterion.

Neither the window nor ratio criterion were able to handle noise in conscious animals without significant lowpass filtering. Simulated facility experiments took almost 2 hours so the recursive regression method was devised to estimate steady-state pressure and flow levels from the expected eye response dynamics to step IOP changes. A key advantage of the fitting method is that spontaneous IOP bumps are progressively ignored as data is accrued (Fig 8). This produces records that grow smoother with time, allowing for quicker detection of steady state without loss of accuracy (Fig 9). Experiment duration could be shortened by 30 to 60 min depending on the simulated perfusion technique. It should be noted that the single exponential fitting function used here is best suited for low-gain CP systems and that a double exponential fitting function may be beneficial for high-gain CP systems where system and eye dynamics distinctly differ.

### Limitations and future work

The study has at least five limitations that could serve to motivate further research with model simulations. Supporting Information containing computer code (S2 Appendix), noise data (S3 Appendix), and simulation data (S4 Appendix) is provided for exploration. One limitation is that the eye is modeled as a linear biomechanical system. This treatment can be justified for in vivo eyes because model simulations resemble pressure and flow recordings from rodent eyes and reported pressure-flow data are rather linear over the IOP range simulated [15, 17, 20, 42]. It may also be justified for enucleated eyes that have been pressurized to normal IOP levels [35, 43] but not eyes that are depressurized. The pressure-flow relation exhibits a power-law nonlinearity as pressure goes to zero [18]. In addition, ocular compliance is known to vary nonlinearly with pressure [24, 44, 45]. The pressure dependence could be incorporated in R_T_ and C_W_ parameters to expand eye model functionality and explore system performance over different operating ranges. A second limitation is that the study explores only a subset of a vast parameter space that includes step size, system gain, filter width, steady state criteria, filter type, and each element of the system and eye. Literature values were used to decrease the parameter space to a region of experimentally relevance, and select parameters were independently varied in this subspace. As such, not all published models and criteria were implemented so the simulation results are not exhaustive. For example, a flow-based criteria has been proposed [24] but was not considered here. It should perform similar to the ratio criterion, though, based on this study since pressure traces were comparatively stable. It would be interesting to investigate whether variations in other model parameters and covariations in multiple parameters, as well as other possible criteria, might lead to more finely-tuned higher-performing systems. A third limitation is that the model neglects contributions of fluid inertia to ocular and system dynamics. Inertial effects would be greatest at CP step onset when flow rate swings precipitously and would increase the likelihood of response ringing, especially with high-gain systems. They should dissipate by the time of steady-state detection and would not be expected to markedly alter simulated response setting times or facility estimates. A fourth limitation is that model simulations assumed noise is additive. The assumption appears reasonable based on published responses to CF and CP step series [17, 20], but it might not be entirely valid because ocular pulse amplitude is reported to increase with IOP due to stretch-induced ocular stiffening [46, 47]. Observed pulse changes were relatively small, increasing 1–1.5 mmHg as IOP was raised 20–25 mmHg above resting level. The pulse amplitude effects presumably reflect the nonlinear dependence of ocular compliance on pressure [24], which flattens out over this pressure range in mice as well. Incorporating a pressure-dependent C_W_ could thus yield multiplicative IOP noise as a byproduct. A fifth limitation is that noise in the simulations was applied to pressure waveforms. If the noise comes from fluid volume changes, such as from a blink, then responses would likely differ as system dynamics would differentially dampen fluid perturbations. The former was simulated because only pressure data is available, but the latter would be interesting to explore.

## Supporting information

S1 Appendix Derivations of governing differential equations of model. (DOCX)

S2 Appendix MATLAB code of eye and eye perfusion model. (DOCX)

S3 Appendix Enucleated, anesthetized, and conscious noise datasets. (ZIP)

S4 Appendix Model simulation data. (ZIP)
