# Supplementary material for: Simulation of gravity- and pump-driven perfusion techniques for measuring outflow facility of ex vivo and in vivo eyes
Source: PLoS One. 2023 Nov 21;18(11):e0294607. doi: 10.1371/journal.pone.0294607 (PMC10662726; doi:10.1371/journal.pone.0294607)
Supplement: S1 Appendix — (DOCX) [file pone.0294607.s001.docx]

*Supporting Information for “Simulation of gravity- and pump-driven perfusion techniques for measuring outflow facility of ex vivo and in vivo eyes”*

**Derivation of governing equations of eye perfusion models**

The lumped parameter model consists of system and eye compartments that are coupled mathematically, as illustrated in Fig 1. The system compartment infuses fluid into the eye compartment via a variable pressure source P in the CPg model or a variable flow source F_S_ in the CF and CPp models. P and F_S_ are related by the equation:

${P\left( t \right)=R_{S}F}_{S}\left( t \right)+P_{S}\left( t \right)$. [A1]

For a high-impedance flow source like a pump, the effect of system fluid dynamics on P_E_ is described by the differential equation:

$F_{S}\left( t \right)=\frac{P_{S}\left( t \right)-P_{E}\left( t \right)}{R_{C}}+C_{S}\frac{dP_{S}\left( t \right)}{dt}$,

$P_{E}\left( t \right)=R_{C}C_{S}\frac{dP_{S}\left( t \right)}{dt}+P_{S}\left( t \right)-{R_{C}F}_{S}\left( t \right)$. [A2]

To specify the effect of ocular fluid dynamics on P_E_, let F represent the total flow acting on viscoelastic elements of the eye:

$F\left( t \right)=F_{A}+\frac{P_{S}\left( t \right)-P_{E}\left( t \right)}{R_{C}}-F_{U}$. [A3]

Let P_W_ represent pressure produced by the fluid volume stored in globe wall compliance. Assuming P_V_ is constant (29), this means:

$F\left( t \right)=\frac{P_{E}\left( t \right)-P_{V}}{R_{T}}+\frac{P_{E}\left( t \right)-P_{W}\left( t \right)}{R_{W}}+C_{W1}\left( \frac{dP_{E}\left( t \right)}{dt}-\frac{dP_{W}\left( t \right)}{dt} \right).$ [A4]

Solving for P_W_ yields two relationships:

$P_{W}\left( t \right)=\left( \frac{R_{W}}{R_{T}}+1 \right)P_{E}\left( t \right)+R_{W}C_{W1}\left( \frac{dP_{E}\left( t \right)}{dt}-\frac{dP_{W}\left( t \right)}{dt} \right)-R_{W}F\left( t \right)-\left( \frac{R_{W}}{R_{T}} \right)P_{V}$ [A5]

and

$\frac{dP_{W}\left( t \right)}{dt}=\frac{P_{E}\left( t \right)-P_{W}\left( t \right)}{R_{W}\left( C_{W1}+C_{W2} \right)}+\left( \frac{C_{W1}}{C_{W1}+C_{W2}} \right)\frac{dP_{E}\left( t \right)}{dt}$ . [A6]

Substituting [A6] into [A5] and rearranging gives:

$P_{W}\left( t \right)=\left( \frac{R_{W}}{R_{T}}+1 \right)P_{E}\left( t \right)-R_{W}F\left( t \right)-\left( \frac{R_{W}}{R_{T}} \right)P_{V}+R_{W}C_{W1}\frac{dP_{E}\left( t \right)}{dt}-R_{W}C_{W1}\left( \frac{P_{E}\left( t \right)-P_{W}\left( t \right)}{R_{W}\left( C_{W1}+C_{W2} \right)}+\left( \frac{C_{W1}}{C_{W1}+C_{W2}} \right)\frac{dP_{E}\left( t \right)}{dt} \right)$,

$\left( 1-\frac{C_{W1}}{C_{W1}+C_{W2}} \right)P_{W}\left( t \right)=\left( \frac{R_{W}}{R_{T}}+1-\frac{C_{W1}}{C_{W1}+C_{W2}} \right)P_{E}\left( t \right)-R_{W}F\left( t \right)-\left( \frac{R_{W}}{R_{T}} \right)P_{V}+R_{W}C_{W1}\left( 1-\frac{C_{W1}}{C_{W1}+C_{W2}} \right)\frac{dP_{E}\left( t \right)}{dt}$ ,

$P_{W}\left( t \right)=\left( \frac{R_{W}C_{W1}+R_{W}C_{W2}+R_{T}C_{W2}}{R_{T}C_{W2}} \right)P_{E}\left( t \right)+R_{W}C_{W1}\frac{dP_{E}\left( t \right)}{dt}-\left( \frac{R_{W}\left( C_{W1}+C_{W2} \right)}{C_{W2}} \right)F\left( t \right)-\left( \frac{R_{W}\left( C_{W1}+C_{W2} \right)}{R_{T}C_{W2}} \right)P_{V}$ . [A7]

Differentiating [A7], inserting into [A4], and rearranging gives:

$F\left( t \right)=\left( \frac{R_{T}+R_{W}}{R_{T}R_{W}} \right)P_{E}\left( t \right)-\left( \frac{1}{R_{T}} \right)P_{V}-\left( \frac{R_{W}C_{W1}+R_{W}C_{W2}+R_{T}C_{W2}}{R_{W}R_{T}C_{W2}} \right)P_{E}\left( t \right)+\frac{C_{W1}+C_{W2}}{C_{W2}}F\left( t \right)+\left( \frac{C_{W1}+C_{W2}}{R_{T}C_{W2}} \right)P_{V}\cdots$

$-C_{W1}\left( \frac{R_{W}C_{W1}+R_{W}C_{W2}+R_{T}C_{W2}}{R_{T}C_{W2}} \right)\frac{dP_{E}\left( t \right)}{dt}+\left( \frac{{C_{W1}R}_{W}\left( C_{W1}+C_{W2} \right)}{C_{W2}} \right)\frac{dF\left( t \right)}{dt}-R_{W}\left( C_{W1} \right)^{2}\frac{d^{2}P_{E}\left( t \right)}{{dt}^{2}} ,$

$\alpha\frac{d^{2}P_{E}\left( t \right)}{{dt}^{2}}+\left( R_{W}C_{W1}+R_{W}C_{W2}+R_{T}C_{W2} \right)\frac{dP_{E}\left( t \right)}{dt}+P_{E}\left( t \right)=R_{T}R_{W}\left( C_{W1}+C_{W2} \right)\frac{dF\left( t \right)}{dt}+R_{T}F\left( t \right)+P_{V}$ [A8]

$where \alpha={R_{T}R}_{W}C_{W1}C_{W2}$ .

Substituting [A3] into [A8] gives:

$\alpha\frac{d^{2}P_{E}\left( t \right)}{{dt}^{2}}+\left( R_{W}C_{W1}+R_{W}C_{W2}+R_{T}C_{W2} \right)\frac{dP_{E}\left( t \right)}{dt}+P_{E}\left( t \right)=\left( \frac{R_{T}R_{W}\left( C_{W1}+C_{W2} \right)}{R_{C}} \right)\left( \frac{{dP}_{S}\left( t \right)}{dt}-\frac{{dP}_{E}\left( t \right)}{dt} \right)\cdots$

${+\left( \frac{R_{T}}{R_{C}} \right)\left( P_{S}\left( t \right)-P_{E}\left( t \right) \right)+R}_{T}\left( F_{A}-F_{U} \right)+P_{V}$ ,

$\alpha\frac{d^{2}P_{E}\left( t \right)}{{dt}^{2}}+\left( \frac{\beta}{R_{C}} \right)\frac{dP_{E}\left( t \right)}{dt}+\left( \frac{{R_{C}+R}_{T}}{R_{C}} \right)P_{E}\left( t \right)=\left( \frac{R_{T}R_{W}\left( C_{W1}+C_{W2} \right)}{R_{C}} \right)\frac{{dP}_{S}\left( t \right)}{dt}+\left( \frac{R_{T}}{R_{C}} \right)P_{S}\left( t \right)+R_{T}\left( F_{A}-F_{U} \right)+P_{V}$ [A9]

$where \beta={R_{C}R}_{W}C_{W1}+{{R_{C}R}_{W}C}_{W2}+R_{C}R_{T}C_{W2}+R_{T}R_{W}C_{W1}+{R_{T}R_{W}C}_{W2}$.

At the start of an experiment (t = 0) the system and eye are at rest (d^2^/dt^2^ = d/dt = 0) and P_S_ and P_E_ are equal, which simplifies to the Goldmann equation.

$\frac{R_{C}+R_{T}}{R_{C}}P_{E}\left( 0 \right)=\frac{R_{T}}{R_{C}}P_{E}\left( 0 \right)+R_{T}\left( F_{A}-F_{U} \right)+P_{V} ,$

$P_{E}\left( 0 \right)=\bar{P}_{E}{=R}_{T}\left( F_{A}-F_{U} \right)+P_{V}$. [A10]

Rearranging and substituting [A10] into [A9] gives:

$\alpha\frac{d^{2}P_{E}\left( t \right)}{{dt}^{2}}+\left( \frac{\beta}{R_{C}} \right)\frac{dP_{E}\left( t \right)}{dt}+\left( \frac{{R_{C}+R}_{T}}{R_{C}} \right)P_{E}\left( t \right)=\left( \frac{R_{T}R_{W}\left( C_{W1}+C_{W2} \right)}{R_{C}} \right)\frac{{dP}_{S}\left( t \right)}{dt}+\left( \frac{R_{T}}{R_{C}} \right)P_{S}\left( t \right)+\bar{P}_{E}$ [A11]

Lastly, system and eye compartments can be combined by inserting [A2] into [A10]:

$\alpha R_{C}C_{S}\frac{d^{3}P_{S}\left( t \right)}{{dt}^{3}}+\alpha\frac{d^{2}P_{S}\left( t \right)}{{dt}^{2}}-{\alpha R}_{C}\frac{d^{2}F_{S}\left( t \right)}{{dt}^{2}}+\beta C_{S}\frac{d^{2}P_{S}\left( t \right)}{{dt}^{2}}+\left( \frac{\beta}{R_{C}} \right)\frac{{dP}_{S}\left( t \right)}{dt}-\beta\frac{{dF}_{S}\left( t \right)}{dt}+C_{S}\left( {R_{C}+R}_{T} \right)\frac{dP_{S}\left( t \right)}{dt}\cdots$

$+{\left( \frac{{R_{C}+R}_{T}}{R_{C}} \right)P}_{S}\left( t \right)-{\left( {R_{C}+R}_{T} \right)F}_{S}\left( t \right)=\left( \frac{R_{T}R_{W}\left( C_{W1}+C_{W2} \right)}{R_{C}} \right)\frac{{dP}_{S}\left( t \right)}{dt}+\left( \frac{R_{T}}{R_{C}} \right)P_{S}\left( t \right)+\bar{P}_{E} ,$

which can be simplified to yield the governing equation for the CF and CPp models:

$\alpha R_{C}C_{S}\frac{d^{3}P_{S}\left( t \right)}{{dt}^{3}}+\left( \alpha+\beta C_{S} \right)\frac{d^{2}P_{S}\left( t \right)}{{dt}^{2}}+\gamma\frac{{dP}_{S}\left( t \right)}{dt}+P_{S}\left( t \right)={\alpha R}_{C}\frac{d^{2}F_{S}\left( t \right)}{{dt}^{2}}+\beta\frac{{dF}_{S}\left( t \right)}{dt}+{\left( {R_{C}+R}_{T} \right)F}_{S}\left( t \right)+\bar{P}_{E}$ [A12]

$where \gamma=R_{C}C_{S}+R_{T}C_{S}+R_{W}C_{W1}+R_{W}C_{W2}+R_{T}C_{W2}$ .

The governing equation for the CPg model can be determined by solving [A1] for F_S_ and substituting into [A12]:

$\alpha R_{C}C_{S}\frac{d^{3}P_{S}\left( t \right)}{{dt}^{3}}+\left( \alpha+\beta C_{S} \right)\frac{d^{2}P_{S}\left( t \right)}{{dt}^{2}}+\gamma\frac{{dP}_{S}\left( t \right)}{dt}+P_{S}\left( t \right)=\left( \frac{{\alpha R}_{C}}{R_{S}} \right)\frac{d^{2}P\left( t \right)}{{dt}^{2}}-\left( \frac{{\alpha R}_{C}}{R_{S}} \right)\frac{d^{2}P_{S}\left( t \right)}{{dt}^{2}}+\left( \frac{\beta}{R_{S}} \right)\frac{dP\left( t \right)}{dt}-\left( \frac{\beta}{R_{S}} \right)\frac{{dP}_{S}\left( t \right)}{dt}\cdots$

${+\left( \frac{{R_{C}+R}_{T}}{R_{S}} \right)P\left( t \right)-\left( \frac{{R_{C}+R}_{T}}{R_{S}} \right)P}_{S}\left( t \right)+\bar{P}_{E}$ ,

which reduces to:

$\alpha R_{C}C_{S}\frac{d^{3}P_{S}\left( t \right)}{{dt}^{3}}+\left( \frac{{\alpha R}_{S}+{\alpha R}_{C}+\beta{R_{S}C}_{S}}{R_{S}} \right)\frac{d^{2}P_{S}\left( t \right)}{{dt}^{2}}+\left( \frac{\gamma R_{S}+\beta}{R_{S}} \right)\frac{{dP}_{S}\left( t \right)}{dt}+\left( \frac{{{R_{S}+R}_{C}+R}_{T}}{R_{S}} \right)P_{S}\left( t \right)=\left( \frac{{\alpha R}_{C}}{R_{S}} \right)\frac{d^{2}P\left( t \right)}{{dt}^{2}}+\left( \frac{\beta}{R_{S}} \right)\frac{dP\left( t \right)}{dt}\cdots$

$+\left( \frac{{R_{C}+R}_{T}}{R_{S}} \right)P\left( t \right)+\bar{P}_{E}$ . [A13]
