# Supplementary material for: Simulation of gravity- and pump-driven perfusion techniques for measuring outflow facility of ex vivo and in vivo eyes
Source: PLoS One. 2023 Nov 21;18(11):e0294607. doi: 10.1371/journal.pone.0294607 (PMC10662726; doi:10.1371/journal.pone.0294607)
Supplement: S2 Appendix — (DOCX) [file pone.0294607.s002.docx]

*Supporting Information for “Simulation of gravity- and pump-driven perfusion techniques for measuring outflow facility of ex vivo and in vivo eyes”*

**MATLAB code for eye perfusion model simulations**

classdef pumpTheory_pub

properties (Constant)

rs = 0.6; %System and Tubing Resistance

cs = 0.05; %System and Tubing Compliance

rc = 0.36; %Cannula Resistance

rt = 1/0.023; %Trabcular Meshwork Resistance

cw1 = 0.18; %Compliant Element of Eye Wall

cw2 = 0.18; %Compliant Element of Viscoelastic Eye Wall

rw = 2.3; %Resistive Element of Viscoelastic Eye Wall

CPg1_Rs = 10; %Gain of CPg1 setup (k = 1/CPg1_Rs)

CPg2_Rs = 0.6; %Gain of CPg2 setup (k = 1/CPg2_Rs)

CPp_k = 7; %CPp feedback gain (k)

CPpx_k = 0.3; %CPpx feedback gain (k)

minTimeBeforeFit = 2; %Min time before using fitting functions for filtering

filtWinCPpx = 15/60; %Filter width for feedback signal in CPpx.

ratioCriteria = 0.0001 %Slope criteria for ratio methods

CPpx_postFiltering = true; %If true, apply the filtering criteria to the CPp post

as with other modes, if false, only pressure if filtered and fed back into algo

trimFilteredData = true; %If true and trueMovingAverage is true, trim filtered

data when filter extends backward more than current setpoint to avoid artifacts

trueMovingAvg = true; %Boolean flag; set false to force moving average window to

exclude any data points from before a new set point. That is, moving average

window will start with width of 1 and grow to desired width

basePath = %Path to folder containting the NoiseSamples Folder

exVivoNoisePath = strcat(pumpTheory_pub.basePath,'NoiseSamples/exVivo_noise.xlsx');

anesNoisePath = strcat(pumpTheory_pub.basePath,'NoiseSamples/anes_noise.xlsx');

awakeNoisePath = strcat(pumpTheory_pub.basePath,'NoiseSamples/awake_noise.xlsx');

outputPath = strcat(pumpTheory_pub.basePath,'SimulationOutputs/');

exVivoNoiseMatPath = strcat(pumpTheory_pub.basePath,'NoiseSamples/exVivoRaw.mat');

end

methods(Static)

function data = paperFigures(figures, save, plott, verbose)

warning('off')

if nargin < 1, figures = [2,3,4,5,6,7,8,9]; end

if nargin < 2, save = false; end

if nargin < 3, plott = false; end

if nargin < 4, verbose = 1; end

%Universal constants used for all figures

restingIOP = 15; %mmHg

stepSize = 5; %mmHg

setpoints = restingIOP+stepSize.*(1:5);

setpoints_CF = 0.1.*(1:5);

fittingTimeConst = 6;

fittingTestWin = 3;

exVivoFilterWidth = struct('CF', 0, 'CPg1', 0, 'CPg2', 0, 'CPp', 0, 'CPpx', 0);

anesFilterWidth = struct('CF', nan, 'CPg1', 1/30, 'CPg2', 3, 'CPp', 3.25, 'CPpx',

1.75); % nan, 1/30, 3, 3.25, nan

awakeFilterWidth = struct('CF', nan, 'CPg1', 10, 'CPg2', 14, 'CPp', 14, 'CPpx',

13); % nan, 48, 59, 59, 56

testWin = 5;

freq = 10; %Hz

exVivoNoiseParams = struct('mu', 0, 'st', 0.04, 'len', 120, 'dt', 1/(freq*60));

% mean, std, length, dt

for i=figures

switch i

case 2

if verbose>=1, disp("Figure "+i); end

%Declare variables needed for excel output

labels = [];

strMatrix = [];

labelTemplate = ["Time", "Flow", "Ps", "Flow_eye", "Pe"];

%Set up options for each mode

simulationTime = 60; %min

setpoint = [stepSize/(pumpTheory_pub.rc+pumpTheory_pub.rt);restingIOP+stepSize;restingIOP+stepSize;restingIOP+stepSize]; %mmHg

modes = ["CF"; "CPg1"; "CPg2"; "CPp"];

kStart = 0.01; kEnd = 10;

RsStart = 0.1; RsEnd = 100;

nPerDecade = 10;

kValues = 10.^(log10(kStart):1/nPerDecade:log10(kEnd))';

RsValues = 10.^(log10(RsStart):1/nPerDecade:log10(RsEnd))';

%Run the simulation for each mode

for modeInd = 1:length(modes)

opt = pumpTheory_pub.createOutflowOptions(modes(modeInd), "time", simulationTime);

[~, ~, tempRaw]=pumpTheory_pub.outflow(modes(modeInd), setpoint(modeInd), freq, opt);

data.(['fig',num2str(i)]).(modes(modeInd)+"_raw") = tempRaw;

tempRaw(:,6) = (tempRaw(:,3)-tempRaw(:,7))./pumpTheory_pub.rc;

[labels, strMatrix] = matrixWrite(labels, strMatrix, labelTemplate, tempRaw(:,[1,2,3,6,7]), "Spacer");

if verbose>=2, disp(" ModeInd: "+modeInd); end

end

%Sweep k for CF case

opt = pumpTheory_pub.createOutflowOptions("CF", "true99");

[~, ~, tempRaw]=pumpTheory_pub.outflow("CF", setpoint(1), freq, opt);

data.(['fig',num2str(i)]).("CF_k_times")(:,1) = tempRaw(end,1)*ones(length(kValues),1);

%Sweep Rs for CPg case

opt = pumpTheory_pub.createOutflowOptions("CPg_Custom", "true99");

for RsInd = 1:length(RsValues)

opt.customK = 1/RsValues(RsInd);

[~, ~, tempRaw]=pumpTheory_pub.outflow("CPg_Custom", setpoint(2), freq, opt);

data.(['fig',num2str(i)]).("CPg_Rs_times")(RsInd,1) = tempRaw(end,1);

if verbose>=2, disp(" RsInd: "+RsInd); end

end

%Sweep k for CPp case

opt = pumpTheory_pub.createOutflowOptions("CPp_Custom", "true99");

for kInd = 1:length(kValues)

opt.customK = kValues(kInd);

[~, ~, tempRaw]=pumpTheory_pub.outflow("CPp_Custom", setpoint(4), freq, opt);

data.(['fig',num2str(i)]).("CPp_k_times")(kInd,1) = tempRaw(end,1);

if verbose>=2, disp(" kInd: "+kInd); end

end

%Save data to excel variables

[labels, strMatrix] = matrixWrite(labels, strMatrix, "CF_K Values", kValues, "Settling Times", data.(['fig',num2str(i)]).("CF_k_times"), "Spacer", "CPg_Rs Values", RsValues, "Settling Times", data.(['fig',num2str(i)]).("CPg_Rs_times"), "Spacer", "CPp_K Values", kValues, "Settling Times", data.(['fig',num2str(i)]).("CPp_k_times"));

%plot the data if specified

if plott

figure;

counter = 0;

CF = data.(['fig',num2str(i)]).("CF_raw");

CPg1 = data.(['fig',num2str(i)]).("CPg1_raw");

CPg2 = data.(['fig',num2str(i)]).("CPg2_raw");

CPp = data.(['fig',num2str(i)]).("CPp_raw");

ax = subplot(3,5,(1:2)+counter);

plot(ax, CF(:,1),CF(:,3),'k',CF(:,1),CF(:,7),'k-.');

xlim([0 1]); xlabel("Time (min)"); ylabel("Pressure (mmHg)");

ax = subplot(3,5,(3:4)+counter);

plot(ax,CF(:,1),CF(:,2),'k',CF(:,1),(CF(:,3)-CF(:,7))./pumpTheory_pub.rc,'k-.');

xlim([0 1]); ylim([0 2.5]); xlabel("Time (min)"); ylabel("Flow (ul/min)");

subplot(3,5,5+counter);

semilogx(kValues, data.(['fig',num2str(i)]).("CF_k_times"));

ylim([0 50]); xlabel("K"); ylabel("Settling Time (min)");

counter=counter+5;

ax = subplot(3,5,(1:2)+counter);

plot(ax, CPg1(:,1),CPg1(:,3),'r',CPg1(:,1),CPg1(:,7),'r-.'); hold on

plot(ax, CPg2(:,1),CPg2(:,3),'k',CPg2(:,1),CPg2(:,7),'k-.');

xlim([0 1]); xlabel("Time (min)"); ylabel("Pressure (mmHg)");

ax = subplot(3,5,(3:4)+counter);

plot(ax, CPg1(:,1),CPg1(:,2),'r',CPg1(:,1),(CPg1(:,3)-CPg1(:,7))./pumpTheory_pub.rc,'r-.'); hold on

plot(ax, CPg2(:,1),CPg2(:,2),'k',CPg2(:,1),(CPg2(:,3)-CPg2(:,7))./pumpTheory_pub.rc,'k-.');

xlim([0 1]); ylim([0 2.5]); xlabel("Time (min)"); ylabel("Flow (ul/min)");

subplot(3,5,5+counter);

semilogx(RsValues, data.(['fig',num2str(i)]).("CPg_Rs_times"));

ylim([0 50]); xlabel("Rs"); ylabel("Settling Time (min)"); hold on;

ind1 = find(RsValues>pumpTheory_pub.CPg1_Rs,2)-1;

val1 = data.(['fig',num2str(i)]).("CPg_Rs_times")(ind1);

ind2 = find(RsValues>pumpTheory_pub.CPg2_Rs,2)-1;

val2 = data.(['fig',num2str(i)]).("CPg_Rs_times")(ind2);

plot(pumpTheory_pub.CPg1_Rs, sum(val1.*(1-abs((RsValues(ind1)-pumpTheory_pub.CPg1_Rs)/diff(RsValues(ind1))))), 'r.');

plot(pumpTheory_pub.CPg2_Rs, sum(val2.*(1-abs((RsValues(ind2)-pumpTheory_pub.CPg2_Rs)/diff(RsValues(ind2))))), 'k.');

counter=counter+5;

ax = subplot(3,5,(1:2)+counter);

plot(ax, CPp(:,1),CPp(:,3),'k',CPp(:,1),CPp(:,7),'k-.');

xlim([0 1]); xlabel("Time (min)"); ylabel("Pressure (mmHg)");

ax = subplot(3,5,(3:4)+counter);

plot(ax, CPp(:,1),CPp(:,2),'k',CPp(:,1),(CPp(:,3)-CPp(:,7))./pumpTheory_pub.rc,'k-.');

xlim([0 1]); ylim([0 2.5]); xlabel("Time (min)"); ylabel("Flow (ul/min)");

subplot(3,5,5+counter);

semilogx(kValues, data.(['fig',num2str(i)]).("CPp_k_times"));

ylim([0 50]); xlabel("K"); ylabel("Settling Time (min)"); hold on

ind1 = find(kValues>pumpTheory_pub.CPp_k,2)-1;

val1 = data.(['fig',num2str(i)]).("CPp_k_times")(ind1);

plot(pumpTheory_pub.CPg1_Rs, sum(val1.*(1-abs((kValues(ind1)-pumpTheory_pub.CPp_k)/diff(kValues(ind1))))), 'r.');

drawnow

end

%Save data to an excel output

strMatrix = [labels;strMatrix];

if save

writematrix(strMatrix, pumpTheory_pub.outputPath+"Figure_"+i+".xlsx");

end

case 3

if verbose>=1, disp("Figure "+i); end

%Constants/controls for figure

noiseExampleNum = 1;

nBin = 10;

%Declare variables needed for excel output

labels = [];

strMatrix = [];

%retrieve the noise signals to usee in th simulation

noise = readmatrix(pumpTheory_pub.exVivoNoisePath);

noiseTime = noise(:,1);

noise = noise(1:length(noise),2:end);

noiseEx = rmmissing(noise(:,noiseExampleNum));

%create a histogram of the a sample noise signal

[bin_Ns, bin_mids] = hist(noiseEx, nBin);

bin_Ns=bin_Ns'; bin_mids = bin_mids';

%Fit gauss curve

guass=fit(bin_mids,bin_Ns/sum(bin_Ns),'gauss1');

gauss_x=(-2:0.01:2)';

gauss_y=feval(guass,gauss_x);

%Calculate the power spectral density function for the noise sample

N = length(noiseEx);

fs = 1/((noiseTime(2)-noiseTime(1))*60);

noisedft = fft(noiseEx);

noisedft = noisedft(1:N/2+1);

psdNoise = (1/(fs*N))*abs(noisedft).^2;

psdNoise(2:end-1) = 2*psdNoise(2:end-1);

psdFreq = (0:fs/length(noiseEx):fs/2)';

%Add to data Structure to be returned and to excel printable matrix as strings

data.(['fig',num2str(i)]).noiseTime = noiseTime;

data.(['fig',num2str(i)]).noiseData = noiseEx;

data.(['fig',num2str(i)]).noiseBins = bin_mids;

data.(['fig',num2str(i)]).noiseHist = bin_Ns;

data.(['fig',num2str(i)]).noiseGaussX = gauss_x;

data.(['fig',num2str(i)]).noiseGauss = gauss_y;

data.(['fig',num2str(i)]).noisePSDX = psdFreq;

data.(['fig',num2str(i)]).noisePSD = psdNoise;

[labels, strMatrix] = matrixWrite(labels, strMatrix, "Time", noiseTime, "Noise", noiseEx, "Spacer", "binCenters", bin_mids, "binCounts", bin_Ns, "Spacer", "Gauss_fit_x", gauss_x, "Gauss_fit_y", gauss_y, "Spacer", "PSD_freq", psdFreq, "PSD_amp", psdNoise);

%Get a sample step response for the CPg2 model for the given noise sample

mode = "CPg2";

noiseExAdj = pumpTheory_pub.genNoise(exVivoNoiseParams);

opts = pumpTheory_pub.createOutflowOptions(mode, "time", 20, 0, "mean", noiseExAdj(:,2));

[~, ~, tempRaw] = pumpTheory_pub.outflow(mode, restingIOP+stepSize, freq, opts);

data.(['fig',num2str(i)]).(mode+"_raw") = tempRaw;

[labels, strMatrix] = matrixWrite(labels, strMatrix, "Spacer", ["Time", "Flow", "Ps", "Pe", "Setpoint"], tempRaw(:,[1,2,3,7,8]));

%Find the trigger times for each steach state criteria (SSC)

SSC = ["window"; "ratio"];

SSC_times = [];

opts.testWindow = 5;

for optsInd = 1:length(SSC)

opts.steadyStateCriteria = SSC(optsInd);

[~, ~, rawData] = pumpTheory_pub.outflow(mode, restingIOP+stepSize, freq, opts);

SSC_times(optsInd,1) = rawData(end,1);

if verbose>=2, disp(" OptsInd: "+optsInd); end

end

data.(['fig',num2str(i)]).(mode+"_SSC")=SSC;

data.(['fig',num2str(i)]).(mode+"_SSC_times")=SSC_times;

[labels, strMatrix] = matrixWrite(labels, strMatrix, "Spacer", "Steady State Criterias", SSC, "Times", SSC_times);

%plot the data if specified

if plott

figure; xWin = 10;

subplot(4,2,1); plot(noiseTime(1:length(noiseEx)), noiseEx);

xlabel("Time (min)"); ylabel("Pressure (mmHg)");

ylim([-0.1 0.1]);

subplot(4,4,3); barh(bin_mids, bin_Ns/(sum(bin_Ns))); hold on

plot(gauss_y,gauss_x);

ylim([-0.1 0.1]);

xlabel("Probability"); ylabel("Pressure (mmHg)");

subplot(4,4,4); loglog(psdFreq, psdNoise, '.');

xlim([0.01 10]); xlabel("Frequency (Hz)"); ylabel("PSD (mmHg^2)");

subplot(4,1,2); plot(tempRaw(:,1), tempRaw(:,3)); hold on

plot([SSC_times(1)-5, SSC_times(1)], tempRaw(find(tempRaw(:,1)>=SSC_times(1),1),3)*[1,1], 'r','linewidth', 2)

xlim([0 xWin]); xlabel("Time (min)"); ylabel("Pressure (mmHg)");

subplot(4,1,3); plot(tempRaw(:,1), tempRaw(:,2));

xlim([0 xWin]); ylim([0 2.5]); xlabel("Time (min)"); ylabel("Flow (ul/min)");

subplot(4,1,4); semilogy(tempRaw(:,1), tempRaw(:,2)./tempRaw(:,3)); hold on

ind = find(tempRaw(:,1)>=SSC_times(2),1);

semilogy([SSC_times(2)-5, SSC_times(2)], (tempRaw(ind,2)/tempRaw(ind,3))*[1,1], 'b','linewidth', 2)

xlim([0 xWin]); xlabel("Time (min)"); ylabel("ratio (ul/min/mmHg)");

drawnow

end

%Save data to an excel output

strMatrix = [labels;strMatrix];

if save

writematrix(strMatrix, pumpTheory_pub.outputPath+"Figure_"+i+".xlsx");

end

case 4

if verbose>=1, disp("Figure "+i); end

%Constants/controls for figure

nBin = 10;

numNoise = 10;

noiseExampleNum = 1;

%Declare variables needed for excel output

labels = [];

strMatrix = [];

%retrieve the noise signals to usee in th simulation

noise = (0:exVivoNoiseParams.dt:exVivoNoiseParams.len-exVivoNoiseParams.dt)';

for noiseInd = 1:numNoise

temp = pumpTheory_pub.genNoise(exVivoNoiseParams);

noise(:,noiseInd+1) = temp(:,2);

end

noiseTime = noise(:,1);

noise = noise(:,2:end);

noiseEx = rmmissing(noise(:,noiseExampleNum));

%create a histogram of the a sample noise signal

[bin_Ns, bin_mids] = hist(noiseEx, nBin);

bin_Ns=bin_Ns'; bin_mids = bin_mids';

%Add to data Structure to be returned and to excel printable matrix as strings

data.(['fig',num2str(i)]).noiseTime = noiseTime;

data.(['fig',num2str(i)]).noiseData = noiseEx;

data.(['fig',num2str(i)]).noiseBins = bin_mids;

data.(['fig',num2str(i)]).noiseHist = bin_Ns;

[labels, strMatrix] = matrixWrite(labels, strMatrix, "Time", noiseTime, "Noise", noiseEx, "Spacer", "binCenters", bin_mids, "binCounts", bin_Ns);

if verbose>=2, disp(" Noise Hist Done"); end

%Create example traces for outflow facility measurement under each condition

%Set up the base settings for each mode

noiseEx = pumpTheory_pub.interpolateNoise(1/(freq*60), noiseTime, noise(:,noiseExampleNum));

CF_opts = pumpTheory_pub.createOutflowOptions("CF", "true99", 0, exVivoFilterWidth.CF, "mean", noiseEx);

CPg1_opts = pumpTheory_pub.createOutflowOptions("CPg1", "true99", 0, exVivoFilterWidth.CPg1, "mean", noiseEx);

CPg2_opts = pumpTheory_pub.createOutflowOptions("CPg2", "true99", 0, exVivoFilterWidth.CPg2, "mean", noiseEx);

CPp_opts = pumpTheory_pub.createOutflowOptions("CPp", "true99", 0, exVivoFilterWidth.CPp, "mean", noiseEx);

%Compile variables to allow for looping later

opts = [CF_opts; CPg1_opts; CPg2_opts; CPp_opts];

modes = ["CF"; "CPg1"; "CPg2"; "CPp"];

%Steady State Criteria Filter testWindow

options = ["ratio", "mean", testWin; "window", "mean", testWin];

%Cycle through different filter and Steady state criteria

%Create the example outflow facility measurement

for optsInd = 1:size(options,1)

for modeInd=1:length(modes)

%update the options for each iteration

opts(modeInd).steadyStateCriteria = options(optsInd,1);

opts(modeInd).filterType = options(optsInd,2);

opts(modeInd).testWindow = str2num(options(optsInd,3));

opts(modeInd).filterWindow = exVivoFilterWidth.(modes(modeInd));

opts(modeInd).downSample = (options(optsInd,2)=="fitting")*opts(modeInd).fittingFrequency;

optStr = "_SS"+options(optsInd,1)+"_FILT"+options(optsInd,2);

data.(['fig',num2str(i)]).(modes(modeInd)+optStr).opts=opts(modeInd);

%run the simulation for the current options/mode

if modes(modeInd)=="CF"

[~, data.(['fig',num2str(i)]).(modes(modeInd)+optStr).outflow_raw, data.(['fig',num2str(i)]).(modes(modeInd)+optStr).raw] = pumpTheory_pub.outflow(modes(modeInd), setpoints_CF, freq, opts(modeInd));

else

[~, data.(['fig',num2str(i)]).(modes(modeInd)+optStr).outflow_raw, data.(['fig',num2str(i)]).(modes(modeInd)+optStr).raw] = pumpTheory_pub.outflow(modes(modeInd), setpoints, freq, opts(modeInd));

end

%Update the strMatrix variable

rawDataLabelTemp = ["Time", "Flow", "FiltFlow", "Ps", "FiltPs", "Setpoint"];

rawOutflowDataLabelTemp = ["Outflow_Ps", "Outflow_Flow", "Outflow_Pe", "Outflow_Ps_std", "Outflow_Flow_std", "Outflow_Pe_std"];

[labels, strMatrix] = matrixWrite(labels, strMatrix, "Spacer", modes(modeInd)+"_"+rawDataLabelTemp+optStr, data.(['fig',num2str(i)]).(modes(modeInd)+optStr).raw(:,[1,2,4,3,5,8]), modes(modeInd)+"_"+rawOutflowDataLabelTemp+optStr, data.(['fig',num2str(i)]).(modes(modeInd)+optStr).outflow_raw(:,[1,2,3,4,5,6]));

if verbose>=2,

disp(" Noise Example | OptsInd: "+optsInd+", ModeInd: "+modeInd);

end

end

end

%Cycle through different filter and steady state conditions for each noise sample and compile results

%Reset the appropriate filter and SSC to cycle through

%Steady State Criteria Filter testWindow FilterWindow

options = ["window", "mean", testWin; "ratio", "mean", testWin;];

%Collect the data

tempOutflows = [];

tempTimes = [];

for modeInd=1:length(modes)

tempLabels=[];

for optsInd = 1:size(options,1)

%update the options for each iteration

opts(modeInd).steadyStateCriteria = options(optsInd,1);

opts(modeInd).filterType = options(optsInd,2);

opts(modeInd).testWindow = str2num(options(optsInd,3));

opts(modeInd).filterWindow = exVivoFilterWidth.(modes(modeInd));

opts(modeInd).downSample = (options(optsInd,2)=="fitting")*opts(modeInd).fittingFrequency;

data.(['fig',num2str(i)]).(modes(modeInd)).opts=opts(modeInd);

%run the simulation for the current options/mode

for noiseInd = 1:size(noise,2)

opts(modeInd).noiseSignal = pumpTheory_pub.interpolateNoise(1/(freq*60), noiseTime, noise(:,noiseInd));

if modes(modeInd)=="CF"

[data.(['fig',num2str(i)]).(modes(modeInd)).outflows(noiseInd,optsInd), ~, temp] = pumpTheory_pub.outflow(modes(modeInd), setpoints_CF, freq, opts(modeInd));

else

[data.(['fig',num2str(i)]).(modes(modeInd)).outflows(noiseInd,optsInd), ~, temp] = pumpTheory_pub.outflow(modes(modeInd), setpoints, freq, opts(modeInd));

end

data.(['fig',num2str(i)]).(modes(modeInd)).times(noiseInd,optsInd) = temp(end,1);

if verbose>=3, disp(" NoiseInd:"+noiseInd); end

end

tempOutflows((1:size(noise,2))+size(noise,2)*(optsInd-1),modeInd) = data.(['fig',num2str(i)]).(modes(modeInd)).outflows(:,optsInd);

tempTimes((1:size(noise,2))+size(noise,2)*(optsInd-1),modeInd) = data.(['fig',num2str(i)]).(modes(modeInd)).times(:,optsInd);

%Update the label variables

tempLabels = [tempLabels; repmat("SS."+options(optsInd,1)+"_FILT."+options(optsInd,2),size(noise,2),1)];

if verbose>=2,

disp(" All Noise | ModeInd: "+modeInd + ", OptsInd: "+optsInd);

end

end

end

[labels, strMatrix] = matrixWrite(labels, strMatrix, "Spacer", "Condition", tempLabels, modes', tempOutflows, "Spacer", "Condition", tempLabels, modes', tempTimes);

%plot the data if specified

if plott

figure;

exampleModeStr = "_SSratio_FILTmean";

x={};

optStrs = "_"+join(replace(options(:,2:-1:1),"ting",""),"_");

temp = nan(size(noise,2),1); %spaceholder for the plot

facilities = []; times = [];

for modeInd=1:length(modes)

%isolate example data

exData = data.(['fig',num2str(i)]).(modes(modeInd)+exampleModeStr);

%plot the raw traces

subplot(3,4,modeInd);

plot(exData.raw(:,1),exData.raw(:,3),'k',exData.raw(:,1),exData.raw(:,5),'r');

xlabel("Time (min)"); ylabel("Pressure (mmHg)");

subplot(3,4,modeInd+4);

plot(exData.raw(:,1),exData.raw(:,2),'k',exData.raw(:,1),exData.raw(:,4),'r');

xlabel("Time (min)"); ylabel("Flow (ul/min)");

%plot the outflow linear estimates

p = [10,20,30,40,45];

subplot(3,6,modeInd+13);

errorbar(exData.outflow_raw(:,1),exData.outflow_raw(:,2),exData.outflow_raw(:,5)

,exData.outflow_raw(:,5),exData.outflow_raw(:,4),exData.outflow_raw(:,4),'o');

fitVal = fitlm(exData.outflow_raw(:,1),exData.outflow_raw(:,2));

m = table2array(fitVal.Coefficients(2,1));

b = table2array(fitVal.Coefficients(1,1));

hold on; plot(p, m*p+b);

xlabel("Pressure (mmHg)"); ylabel("Flow (ul/min)");

%Set up summary plots

x(1+(modeInd-1)*(length(optStrs)+1)) = cellstr(repmat('.',1,modeInd));

x((1:length(optStrs))+1+(modeInd-1)*(length(optStrs)+1)) = cellstr(modes(modeInd)+optStrs);

facilities = [facilities, temp, data.(['fig',num2str(i)]).(modes(modeInd)).outflows];

times = [times, temp, data.(['fig',num2str(i)]).(modes(modeInd)).times];

end

x(1+length(modes)*(length(optStrs)+1)) = cellstr(repmat('.',1,length(modes)+1));

times = [times, temp];

facilities = [facilities, temp];

facilities(facilities>0.1|facilities<0)=NaN;

%plot the summary bar plots for facility and times

subplot(3,6,13);

plotbar(x,times);

hold on; plot([1:size(times,2)], times,'.');

ylabel("Times (min)");

subplot(3,6,18);

plotbar(x,facilities);

hold on; plot([1:size(facilities,2)], facilities,'.');

ylabel("Facilities (ul/min/mmHg)");

drawnow

end

%Save data to an excel output

strMatrix = [labels;strMatrix];

if save

writematrix(strMatrix, pumpTheory_pub.outputPath+"Figure_"+i+".xlsx");

end

case 5

if verbose>=1, disp("Figure "+i); end

%Constants/controls for figure

noiseExampleNum = 2;

nBin = 30;

%Declare variables needed for excel output

labels = [];

strMatrix = [];

%retrieve the noise signals to usee in th simulation

noise = readmatrix(pumpTheory_pub.anesNoisePath);

noiseTime = noise(:,1);

noise = noise(1:length(noise),2:end);

noiseEx = rmmissing(noise(:,noiseExampleNum));

%create a histogram of the a sample noise signal

[bin_Ns, bin_mids] = hist(noiseEx, nBin);

bin_Ns=bin_Ns'; bin_mids = bin_mids';

%Fit gauss curve

guass=fit(bin_mids,bin_Ns/sum(bin_Ns),'gauss1');

gauss_x=(-2:0.01:2)';

gauss_y=feval(guass,gauss_x);

%Add to data Structure to be returned and to the excel printable matrix as strings

data.(['fig',num2str(i)]).noiseTime = noiseTime;

data.(['fig',num2str(i)]).noiseData = noiseEx;

data.(['fig',num2str(i)]).noiseBins = bin_mids;

data.(['fig',num2str(i)]).noiseHist = bin_Ns;

data.(['fig',num2str(i)]).noiseGaussX = gauss_x;

data.(['fig',num2str(i)]).noiseGauss = gauss_y;

[labels, strMatrix] = matrixWrite(labels, strMatrix, "Time", noiseTime, "Noise", noiseEx, "Spacer", "binCenters", bin_mids, "binCounts", bin_Ns, "Spacer", "Gauss_fit_x", gauss_x, "Gauss_fit_y", gauss_y);

if verbose>=2, disp(" Noise Hist Done"); end

%Get a sample step response for the CPg2 model for the given noise sample

mode = "CPg2";

noiseExAdj = pumpTheory_pub.interpolateNoise(1/(freq*60), noiseTime(1:length(noiseEx)), noiseEx);

opts = pumpTheory_pub.createOutflowOptions(mode, "time", 90, anesFilterWidth.(mode), "mean", noiseExAdj);

[~, ~, tempRaw] = pumpTheory_pub.outflow(mode, restingIOP+stepSize, freq, opts);

data.(['fig',num2str(i)]).(mode+"_raw") = tempRaw;

tempRaw(:,9:10) = [tempRaw(:,2)./tempRaw(:,3), tempRaw(:,4)./tempRaw(:,5)];

[labels, strMatrix] = matrixWrite(labels, strMatrix, "Spacer", ["Time", "Flow", "Ps", "meanFlow", "meanPs", "ratio", "meanRatio", "Pe", "Setpoint"], tempRaw(:,[1,2,3,4,5,9,10,7,8]));

%Find the trigger times for each steach state criteria (SSC)

SSC = ["window"; "ratio"];

SSC_times = [];

opts.testWindow = 5;

for optsInd = 1:length(SSC)

opts.steadyStateCriteria = SSC(optsInd);

[~, ~, rawData] = pumpTheory_pub.outflow(mode, restingIOP+stepSize, freq, opts);

SSC_times(optsInd,1) = rawData(end,1);

end

data.(['fig',num2str(i)]).(mode+"_SSC")=SSC;

data.(['fig',num2str(i)]).(mode+"_SSC_times")=SSC_times;

[labels, strMatrix] = matrixWrite(labels, strMatrix, "Spacer", "Steady State Criterias", SSC, "Times", SSC_times);

if verbose>=2, disp(" Noise Example Done"); end

%Effect of filterWidth on settle time for example Noise and averaged for all noise

%Set up the base settings for each mode

CPg1_opts = pumpTheory_pub.createOutflowOptions("CPg1", "true99", 0, anesFilterWidth.CPg1, "mean", noiseExAdj);

CPg2_opts = pumpTheory_pub.createOutflowOptions("CPg2", "true99", 0, anesFilterWidth.CPg2, "mean", noiseExAdj);

CPp_opts = pumpTheory_pub.createOutflowOptions("CPp", "true99", 0, anesFilterWidth.CPp, "mean", noiseExAdj);

%Compile variables to allow for looping later

opts = [CPg1_opts; CPg2_opts; CPp_opts];

modes = ["CPg1"; "CPg2"; "CPp"];

%Steady State Criteria Filter testWindow

options = ["window", "mean", testWin; "ratio", "mean", testWin];

filtWindows = (0:15)';

%Collect data for each mode, options, noise signal, and filter width

for modeInd = 1:length(modes)

for optsInd = 1:size(options,1)

opts(modeInd).steadyStateCriteria = options(optsInd,1);

opts(modeInd).filterType = options(optsInd,2);

opts(modeInd).testWindow = str2num(options(optsInd,3));

optStr = "_SS"+options(optsInd,1);

data.(['fig',num2str(i)]).(modes(modeInd)+optStr).opts=opts(modeInd);

for noiseInd = 1:size(noise,2)

opts(modeInd).noiseSignal = pumpTheory_pub.interpolateNoise(1/(freq*60), noiseTime(1:length(rmmissing(noise(:,noiseInd)))), rmmissing(noise(:,noiseInd)));

for filtInd = 1:length(filtWindows)

opts(modeInd).filterWindow = filtWindows(filtInd);

[~, ~, rawData] = pumpTheory_pub.outflow(modes(modeInd), restingIOP+stepSize, freq, opts(modeInd));

if isnan(rawData(end,1))

data.(['fig',num2str(i)]).(modes(modeInd)+optStr).times(filtInd,noiseInd)= opts(modeInd).timeout;

else

data.(['fig',num2str(i)]).(modes(modeInd)+optStr).times(filtInd,noiseInd) = rawData(end,1);

end

if verbose>=3,

disp(" NoiseInd: "+noiseInd + ", filtInd: "+filtInd);

end

end

end

if verbose>=2,

disp(" All Noise | ModeInd: "+modeInd + ", OptsInd: "+optsInd);

end

end

%Add data to excel variables

[labels, strMatrix] = matrixWrite(labels, strMatrix, "Spacer", "FilterWidth", filtWindows);

for optsInd = 1:size(options,1)

temp = data.(['fig',num2str(i)]).(modes(modeInd)+"_SS"+options(optsInd,1));

[labels, strMatrix] = matrixWrite(labels, strMatrix, options(optsInd,1)+"_"+modes(modeInd), temp.times(:,noiseExampleNum));

end

[labels, strMatrix] = matrixWrite(labels, strMatrix, "Spacer", "FilterWidth", filtWindows);

for optsInd = 1:size(options,1)

temp = data.(['fig',num2str(i)]).(modes(modeInd)+"_SS"+options(optsInd,1));

[labels, strMatrix] = matrixWrite(labels, strMatrix, "Avg_"+options(optsInd,1)+"_"+modes(modeInd), mean(temp.times,2), "Std_"+options(optsInd,1)+"_"+modes(modeInd), std(temp.times,0,2));

end

end

%plot the data if specified

if plott

figure; xWin = 30;

ax=subplot(5,5,1:4); plot(ax,noiseTime(1:length(noiseEx)), noiseEx);

xlabel("Time (min)"); ylabel("Pressure (mmHg)");

ylim([-1.5 1.5]);

subplot(5,5,5); barh(bin_mids, bin_Ns/(sum(bin_Ns))); hold on

plot(gauss_y,gauss_x);

ylim([-1.5 1.5]);

xlabel("Probability"); ylabel("Pressure (mmHg)");

subplot(5,1,2); plot(tempRaw(:,1), tempRaw(:,3), 'k', tempRaw(:,1), tempRaw(:,5), 'r'); hold on

plot([SSC_times(1)-5, SSC_times(1)], tempRaw(find(tempRaw(:,1)>=SSC_times(1),1),3)*[1,1], 'r','linewidth', 2)

xlim([0 xWin]); xlabel("Time (min)"); ylabel("Pressure (mmHg)");

subplot(5,1,3); plot(tempRaw(:,1), tempRaw(:,2), 'k', tempRaw(:,1), tempRaw(:,4), 'r');

xlim([0 xWin]); ylim([0 2.5]); xlabel("Time (min)"); ylabel("Flow (ul/min)");

subplot(5,1,4); semilogy(tempRaw(:,1), tempRaw(:,8), 'k', tempRaw(:,1), tempRaw(:,9), 'r'); hold on

ind = find(tempRaw(:,1)>=SSC_times(2),1);

semilogy([SSC_times(2)-5, SSC_times(2)], tempRaw(ind,8)*[1,1], 'b','linewidth', 2)

xlim([0 xWin]); xlabel("Time (min)"); ylabel("ratio (ul/min/mmHg)");

optColors = ['r'; 'b'];

for modeInd = 1:length(modes)

subplot(5,length(modes),4*length(modes)+modeInd);

for optsInd = 1:size(options,1)

temp = data.(['fig',num2str(i)]).(modes(modeInd)+"_SS"+options(optsInd,1));

plot(filtWindows, mean(temp.times,2), optColors(optsInd));hold on

plot(filtWindows, temp.times(:,noiseExampleNum), [optColors(optsInd),'.']);

end

xlabel("Filter Width"); ylabel("Settling Time (min)");

end

drawnow

end

%Save data to an excel output

strMatrix = [labels;strMatrix];

if save

writematrix(strMatrix, pumpTheory_pub.outputPath+"Figure_"+i+".xlsx");

end

case 6

if verbose>=1, disp("Figure "+i); end

%Constants/controls for figure

noiseExampleNum = 1;

kValues = [0.1, 0.3, 1, 7];

filtValues = [5, 15, 60, 300]./60; %sec with conversion to min

k=0.3;

filtWin = 15/60;

%Declare variables needed for excel output

labels = [];

strMatrix = [];

%Read in noise sample

noise = readmatrix(pumpTheory_pub.anesNoisePath);

noiseTime = noise(:,1);

noise = rmmissing(noise(1:length(noise),noiseExampleNum+1));

noiseTime = noiseTime(1:length(noise));

[labels, strMatrix] = matrixWrite(labels, strMatrix, "Time", noiseTime, "Noise", noise, "Spacer");

noiseEx = pumpTheory_pub.interpolateNoise(1/(freq*60), noiseTime, noise);

%Baseline

opt = pumpTheory_pub.createOutflowOptions("CPpx_Custom", "time", 10, 0, "mean", noiseEx);

opt.customK = pumpTheory_pub.CPp_k;

[~, ~, tempRawBaseline]=pumpTheory_pub.outflow("CPpx_Custom", restingIOP+stepSize, freq, opt);

data.(['fig',num2str(i)]).("CPpx_Baseline") = tempRawBaseline;

[labels, strMatrix] = matrixWrite(labels, strMatrix, ["Time", "Flow", "Ps"], tempRawBaseline(:,1:3));

%Do a sweep of the filter range

opt = pumpTheory_pub.createOutflowOptions("CPpx_Custom", "time", 10, 0, "mean", noiseEx);

opt.customK = k;

opt.customCPpFilt = filtWin;

for filtInd = 1:length(filtValues)

opt.customCPpFilt = filtValues(filtInd);

[~, ~, tempRaw]=pumpTheory_pub.outflow("CPpx_Custom", restingIOP+stepSize, freq, opt);

data.(['fig',num2str(i)]).("CPpx_filt_"+filtValues(filtInd)*60+"sec") = tempRaw;

[labels, strMatrix] = matrixWrite(labels, strMatrix, ["Flow_filt_"+filtValues(filtInd), "Ps_filt_"+filtValues(filtInd)], tempRaw(:,4:5));

if verbose>=2, disp(" filtInd: "+filtInd); end

end

[labels, strMatrix] = matrixWrite(labels, strMatrix, ["Time", "Flow", "Ps"], tempRawBaseline(:,1:3));

%Do a sweep of the k range

opt = pumpTheory_pub.createOutflowOptions("CPpx_Custom", "time", 10, 0, "mean", noiseEx);

opt.customCPpFilt = filtWin;

for kInd = 1:length(kValues)

opt.customK = kValues(kInd);

[~, ~, tempRaw]=pumpTheory_pub.outflow("CPpx_Custom", restingIOP+stepSize, freq, opt);

data.(['fig',num2str(i)]).("CPpx_k_"+replace(num2str(kValues(kInd)),'.','_')) = tempRaw;

[labels, strMatrix] = matrixWrite(labels, strMatrix, ["Flow_k_"+kValues(kInd), "Ps_k_"+kValues(kInd)], tempRaw(:,4:5));

if verbose>=2, disp(" kInd: "+kInd); end

end

%plot the data if specified

if plott

figure;

colors = ['g'; 'r'; 'b'; 'm'];

for kInd = 1:length(kValues)

tempRaw = data.(['fig',num2str(i)]).("CPpx_k_"+replace(num2str(kValues(kInd)),'.','_'));

if kInd==1

subplot(2,2,1); plot(tempRawBaseline(:,1), tempRawBaseline(:,3), 'k'); hold on

subplot(2,2,3); plot(tempRawBaseline(:,1), tempRawBaseline(:,2), 'k'); hold on

end

subplot(2,2,1); plot(tempRaw(:,1), tempRaw(:,5), colors(kInd)); hold on

subplot(2,2,3); plot(tempRaw(:,1), tempRaw(:,4), colors(kInd)); hold on

end

subplot(2,2,1); xlim([0 4]); ylim([15 22]); xlabel("Time (min)"); ylabel("Pressure (mmHg)");

subplot(2,2,3); xlim([0 4]); ylim([0 2.5]); xlabel("Time (min)"); ylabel("Flow (ul/min)");

for filtInd = 1:length(filtValues)

tempRaw = data.(['fig',num2str(i)]).("CPpx_filt_"+filtValues(filtInd)*60+"sec");

if filtInd==1

subplot(2,2,2); plot(tempRawBaseline(:,1), tempRawBaseline(:,3), 'k'); hold on

subplot(2,2,4); plot(tempRawBaseline(:,1), tempRawBaseline(:,2), 'k'); hold on

end

subplot(2,2,2); plot(tempRaw(:,1), tempRaw(:,5), colors(filtInd)); hold on

subplot(2,2,4); plot(tempRaw(:,1), tempRaw(:,4), colors(filtInd)); hold on

end

subplot(2,2,2); xlim([0 4]); ylim([15 22]); xlabel("Time (min)"); ylabel("Pressure (mmHg)");

subplot(2,2,4); xlim([0 4]); ylim([0 2.5]); xlabel("Time (min)"); ylabel("Flow (ul/min)");

drawnow

end

%Save data to an excel output

strMatrix = [labels;strMatrix];

if save

writematrix(strMatrix, pumpTheory_pub.outputPath+"Figure_"+i+".xlsx");

end

case 7

if verbose>=1, disp("Figure "+i); end

%Constants/controls for figure

noiseExampleNum = 2;

nBin = 30;

%Declare variables needed for excel output

labels = [];

strMatrix = [];

%retrieve the noise signals to usee in th simulation

noise = readmatrix(pumpTheory_pub.anesNoisePath);

noiseTime = noise(:,1);

noise = noise(:,2:end);

noiseEx = rmmissing(noise(:,noiseExampleNum));

%create a histogram of the a sample noise signal

[bin_Ns, bin_mids] = hist(noiseEx, nBin);

bin_Ns=bin_Ns'; bin_mids = bin_mids';

%Add to data Structure to be returned and to the excel printable matrix as strings

data.(['fig',num2str(i)]).noiseTime = noiseTime;

data.(['fig',num2str(i)]).noiseData = noiseEx;

data.(['fig',num2str(i)]).noiseBins = bin_mids;

data.(['fig',num2str(i)]).noiseHist = bin_Ns;

[labels, strMatrix] = matrixWrite(labels, strMatrix, "Time", noiseTime, "Noise", noiseEx, "Spacer", "binCenters", bin_mids, "binCounts", bin_Ns);

if verbose>=2, disp(" Noise Hist Done"); end

%Create the example traces for outflow facility measurement under each condition

%Set up the base settings for each mode

noiseEx = pumpTheory_pub.interpolateNoise(1/(freq*60), noiseTime(1:length(noiseEx)), noiseEx);

CPg1_opts = pumpTheory_pub.createOutflowOptions("CPg1", "true99", 0, anesFilterWidth.CPg1, "mean", noiseEx);

CPg2_opts = pumpTheory_pub.createOutflowOptions("CPg2", "true99", 0, anesFilterWidth.CPg2, "mean", noiseEx);

CPp_opts = pumpTheory_pub.createOutflowOptions("CPp", "true99", 0, anesFilterWidth.CPp, "mean", noiseEx);

CPpx_opts = pumpTheory_pub.createOutflowOptions("CPpx", "true99", 0, anesFilterWidth.CPpx, "mean", noiseEx);

%Compile variables to allow for looping later

opts = [CPg1_opts; CPg2_opts; CPp_opts; CPpx_opts];

modes = ["CPg1"; "CPg2"; "CPp"; "CPpx"];

%Steady State Criteria Filter testWindow FilterWindow

options = ["window", "mean", testWin; "ratio", "mean", testWin];

%Cycle through different filter and Steady state criteria and create example outflow facility measurement

for optsInd = 1:size(options,1)

for modeInd=length(modes):-1:1

%update the options for each iteration

opts(modeInd).steadyStateCriteria = options(optsInd,1);

opts(modeInd).filterType = options(optsInd,2);

opts(modeInd).testWindow = str2num(options(optsInd,3));

opts(modeInd).filterWindow = anesFilterWidth.(modes(modeInd));

optStr = "_SS"+options(optsInd,1)+"_FILT"+options(optsInd,2);

data.(['fig',num2str(i)]).(modes(modeInd)+optStr).opts=opts(modeInd);

%run the simulation for the current options/mode

[~, data.(['fig',num2str(i)]).(modes(modeInd)+optStr).outflow_raw, data.(['fig',num2str(i)]).(modes(modeInd)+optStr).raw] = pumpTheory_pub.outflow(modes(modeInd), setpoints, freq, opts(modeInd));

%Update the strMatrix variable

rawDataLabelTemp = ["Time", "Flow", "FiltFlow", "Ps", "FiltPs", "Setpoint"];

rawOutflowDataLabelTemp = ["Outflow_Ps", "Outflow_Flow", "Outflow_Pe", "Outflow_Ps_std", "Outflow_Flow_std", "Outflow_Pe_std"];

[labels, strMatrix] = matrixWrite(labels, strMatrix, "Spacer", modes(modeInd)+"_"+rawDataLabelTemp+optStr, data.(['fig',num2str(i)]).(modes(modeInd)+optStr).raw(:,[1,2,4,3,5,8]), modes(modeInd)+"_"+rawOutflowDataLabelTemp+optStr, data.(['fig',num2str(i)]).(modes(modeInd)+optStr).outflow_raw(:,[1,2,3,4,5,6]));

if verbose>=2,

disp(" Noise Example | optsInd: "+optsInd + ", ModeInd: "+modeInd);

end

end

end

%Cycle through different filter and steady state conditions for each noise sample and compile results

%Reset the appropriate filter and SSC to cycle through

%Steady State Criteria Filter testWindow FilterWindow

options = ["window", "mean", testWin; "ratio", "mean", testWin];

%Collect the data

tempOutflows = [];

tempTimes = [];

for modeInd=1:length(modes)

tempLabels=[];

for optsInd = 1:size(options,1)

%update the options for each iteration

opts(modeInd).steadyStateCriteria = options(optsInd,1);

opts(modeInd).filterType = options(optsInd,2);

opts(modeInd).testWindow = str2num(options(optsInd,3));

opts(modeInd).filterWindow = anesFilterWidth.(modes(modeInd));

opts(modeInd).downSample = (options(optsInd,2)=="fitting")*opts(modeInd).fittingFrequency;

data.(['fig',num2str(i)]).(modes(modeInd)).opts=opts(modeInd);

%run the simulation for the current options/mode

for noiseInd = 1:size(noise,2)

opts(modeInd).noiseSignal = pumpTheory_pub.interpolateNoise(1/(freq*60), noiseTime(1:length(rmmissing(noise(:,noiseInd)))), rmmissing(noise(:,noiseInd)));

[data.(['fig',num2str(i)]).(modes(modeInd)).outflows(noiseInd,optsInd), ~, temp] = pumpTheory_pub.outflow(modes(modeInd), setpoints, freq, opts(modeInd));

data.(['fig',num2str(i)]).(modes(modeInd)).times(noiseInd,optsInd) = temp(end,1);

if verbose>=3, disp(" NoiseInd: "+noiseInd); end

end

tempOutflows((1:size(noise,2))+size(noise,2)*(optsInd-1),modeInd) = data.(['fig',num2str(i)]).(modes(modeInd)).outflows(:,optsInd);

tempTimes((1:size(noise,2))+size(noise,2)*(optsInd-1),modeInd) = data.(['fig',num2str(i)]).(modes(modeInd)).times(:,optsInd);

%Update the label variables

tempLabels = [tempLabels; repmat("SS."+options(optsInd,1)+"_FILT."+options(optsInd,2),size(noise,2),1)];

if verbose>=2,

disp(" All Noise | ModeInd: "+modeInd + ", OptsInd: "+optsInd);

end

end

end

[labels, strMatrix] = matrixWrite(labels, strMatrix, "Spacer", "Condition", tempLabels, modes', tempOutflows, "Spacer", "Condition", tempLabels, modes', tempTimes);

%plot the data if specified

if plott

figure;

exampleModeStr = "_SSratio_FILTmean";

x={};

optStrs = "_"+join(replace(options(:,2:-1:1),"ting",""),"_");

temp = nan(size(noise,2),1); %spaceholder for the plot

facilities = [];

times = [];

for modeInd=1:length(modes)

%isolate example data

exData = data.(['fig',num2str(i)]).(modes(modeInd)+exampleModeStr);

%plot the raw traces

subplot(3,4,modeInd);

plot(exData.raw(:,1),exData.raw(:,3),'k',exData.raw(:,1),exData.raw(:,5),'r');

xlabel("Time (min)"); ylabel("Pressure (mmHg)");

subplot(3,4,modeInd+4);

plot(exData.raw(:,1),exData.raw(:,2),'k',exData.raw(:,1),exData.raw(:,4),'r');

xlabel("Time (min)"); ylabel("Flow (ul/min)");

%plot the outflow linear estimates

p = [10,20,30,40,45];

subplot(3,6,modeInd+13);

errorbar(exData.outflow_raw(:,1),exData.outflow_raw(:,2),

exData.outflow_raw(:,5),exData.outflow_raw(:,5),exData.outflow_raw(:,4),exData.outflow_raw(:,4),'o');

fitVal = fitlm(exData.outflow_raw(:,1),exData.outflow_raw(:,2));

m = table2array(fitVal.Coefficients(2,1));

b = table2array(fitVal.Coefficients(1,1));

hold on; plot(p, m*p+b);

xlabel("Pressure (mmHg)"); ylabel("Flow (ul/min)");

%Set up summary plots

x(1+(modeInd-1)*(length(optStrs)+1)) = cellstr(repmat('.',1,modeInd));

x((1:length(optStrs))+1+(modeInd-1)*(length(optStrs)+1)) = cellstr(modes(modeInd)+optStrs);

facilities = [facilities, temp, data.(['fig',num2str(i)]).(modes(modeInd)).outflows];

times = [times, temp, data.(['fig',num2str(i)]).(modes(modeInd)).times];

end

x(1+length(modes)*(length(optStrs)+1)) = cellstr(repmat('.',1,length(modes)+1));

times = [times, temp];

facilities = [facilities, temp];

facilities(facilities>0.1|facilities<0)=NaN;

%plot the summary bar plots for facility and times

subplot(3,6,13);

plotbar(x,times);

hold on; plot([1:size(times,2)], times,'.');

ylabel("Times (min)");

subplot(3,6,18);

plotbar(x,facilities);

hold on; plot([1:size(facilities,2)], facilities,'.');

ylabel("Facilities (ul/min/mmHg)");

drawnow

end

%Save data to an excel output

strMatrix = [labels;strMatrix];

if save

writematrix(strMatrix, pumpTheory_pub.outputPath+"Figure_"+i+".xlsx");

end

case 8

if verbose>=1, disp("Figure "+i); end

%Constants/controls for figure

noiseExampleNum = 11;

nBin = 30;

%Declare variables needed for excel output

labels = [];

strMatrix = [];

%retrieve the noise signals to usee in th simulation

noise = readmatrix(pumpTheory_pub.awakeNoisePath);

noiseTime = noise(:,1);

noise = noise(1:length(noise),2:end);

noiseEx = rmmissing(noise(:,noiseExampleNum));

%create a histogram of the a sample noise signal

[bin_Ns, bin_mids] = hist(noiseEx, nBin);

bin_Ns=bin_Ns'; bin_mids = bin_mids';

%Fit gauss curve

guass=fit(bin_mids,bin_Ns/sum(bin_Ns),'gauss1');

gauss_x=(-2:0.01:2)';

gauss_y=feval(guass,gauss_x);

%Add to data Structure to be returned and to excel printable matrix as strings

data.(['fig',num2str(i)]).noiseTime = noiseTime;

data.(['fig',num2str(i)]).noiseData = noiseEx;

data.(['fig',num2str(i)]).noiseBins = bin_mids;

data.(['fig',num2str(i)]).noiseHist = bin_Ns;

data.(['fig',num2str(i)]).noiseGaussX = gauss_x;

data.(['fig',num2str(i)]).noiseGauss = gauss_y;

[labels, strMatrix] = matrixWrite(labels, strMatrix, "Time", noiseTime, "Noise", noiseEx, "Spacer", "binCenters", bin_mids, "binCounts", bin_Ns, "Spacer", "Gauss_fit_x", gauss_x, "Gauss_fit_y", gauss_y);

if verbose>=2, disp(" Noise Hist Done"); end

%Get a sample step response for the CPg2 model for the given noise sample

mode = "CPg2";

noiseExAdj = pumpTheory_pub.interpolateNoise(1/(freq*60), noiseTime(1:length(noiseEx)), noiseEx);

opts = pumpTheory_pub.createOutflowOptions(mode, "time", 90, awakeFilterWidth.(mode), "mean", noiseExAdj);

[~, ~, tempRaw] = pumpTheory_pub.outflow(mode, restingIOP+stepSize, freq, opts);

opts.filterWindow = anesFilterWidth.(mode);

[~, ~, tempRaw3] = pumpTheory_pub.outflow(mode, restingIOP+stepSize, freq, opts);

opts2 = pumpTheory_pub.createOutflowOptions(mode, "time", 90, 0, "fitting", noiseExAdj);

[~, ~, tempRaw2] = pumpTheory_pub.outflow(mode, restingIOP+stepSize, freq, opts2);

data.(['fig',num2str(i)]).(mode+"_FILTmean_raw") = tempRaw;

data.(['fig',num2str(i)]).(mode+"_FILTmean_anes_raw") = tempRaw3;

data.(['fig',num2str(i)]).(mode+"_FILTfitting_raw") = tempRaw2;

tempRaw(:,1:14) = [tempRaw(:,[1,2,3,4,5]), tempRaw3(:,[4,5]), tempRaw2(:,[4,5]), tempRaw(:,2)./tempRaw(:,3), tempRaw(:,4)./tempRaw(:,5), tempRaw3(:,4)./tempRaw2(:,5), tempRaw2(:,4)./tempRaw2(:,5), tempRaw(:,7)];

[labels, strMatrix] = matrixWrite(labels, strMatrix, "Spacer", ["Time", "Flow", "Ps", "meanFlow", "meanPs", "meanFlow_anes", "meanPs_anes", "fittingFlow", "fittingPs", "ratio", "meanRatio", "meanRatio_anes", "fittingRatio", "Pe", "Setpoint"], tempRaw);

%Find the trigger times for each steach state criteria (SSC)

%Steady State Criteria Filter testWindow FilterWindow

options = ["window", "mean", testWin;

"ratio", "mean", testWin;

"window", "fitting", testWin;

"ratio", "fitting", testWin;

"fitting_"+fittingTimeConst, "fitting", fittingTestWin];

SSC = [""];

SSC_times = [];

opts.testWindow = 5;

for optsInd = 1:size(options,1)

opts.steadyStateCriteria = options(optsInd,1);

opts.filterType = options(optsInd,2);

opts.testWindow = str2num(options(optsInd,3));

opts.filterWindow = awakeFilterWidth.(mode)*~contains(opts.filterType,"fitting");

opts.downSample = (options(optsInd,2)=="fitting")*opts.fittingFrequency;

optStr = "_SS"+options(optsInd,1)+"_FILT"+options(optsInd,2);

[~, ~, rawData] = pumpTheory_pub.outflow(mode, restingIOP+stepSize, freq, opts);

SSC_times(optsInd,1) = rawData(end,1);

SSC(optsInd,1) = mode+optStr;

end

for optsInd = 1:2

opts.steadyStateCriteria = options(optsInd,1);

opts.filterType = options(optsInd,2);

opts.testWindow = str2num(options(optsInd,3));

opts.filterWindow = anesFilterWidth.(mode)*~contains(opts.filterType,"fitting");

opts.downSample = (options(optsInd,2)=="fitting")*opts.fittingFrequency;

optStr = "_SS"+options(optsInd,1)+"_FILT"+options(optsInd,2)+"_anes";

[~, ~, rawData] = pumpTheory_pub.outflow(mode, restingIOP+stepSize, freq, opts);

SSC_times(optsInd+size(options,1),1) = rawData(end,1);

SSC(optsInd+size(options,1),1) = mode+optStr;

end

data.(['fig',num2str(i)]).(mode+"_SSC")=SSC;

data.(['fig',num2str(i)]).(mode+"_SSC_times")=SSC_times;

[labels, strMatrix] = matrixWrite(labels, strMatrix, "Spacer", "Steady State Criterias", SSC, "Times", SSC_times);

if verbose>=2, disp(" Noise Example Done"); end

%Effect of filterWidth on settle time for example Noise and average of all noise

%Set up the base settings for each mode

CPg1_opts = pumpTheory_pub.createOutflowOptions("CPg1", "true99", 0, awakeFilterWidth.CPg1, "mean", noiseExAdj);

CPg2_opts = pumpTheory_pub.createOutflowOptions("CPg2", "true99", 0, awakeFilterWidth.CPg2, "mean", noiseExAdj);

CPp_opts = pumpTheory_pub.createOutflowOptions("CPp", "true99", 0, awakeFilterWidth.CPp, "mean", noiseExAdj);

CPpx_opts = pumpTheory_pub.createOutflowOptions("CPpx", "true99", 0, awakeFilterWidth.CPpx, "mean", noiseExAdj);

%Compile variables to allow for looping later

opts = [CPg1_opts; CPg2_opts; CPp_opts; CPpx_opts];

modes = ["CPg1"; "CPg2"; "CPp"; "CPpx"];

%Steady State Criteria Filter testWindow FilterWindow

%Steady State Criteria Filter testWindow FilterWindow

options = ["window", "mean", testWin;

"ratio", "mean", testWin;

"window", "fitting", testWin;

"ratio", "fitting", testWin;

"fitting_"+fittingTimeConst, "fitting", fittingTestWin];

filtWindows = (0:20)';

%Collect data for each mode, options, noise signal, and filter width

for modeInd = 1:length(modes)

for optsInd = 1:size(options,1)

opts(modeInd).steadyStateCriteria = options(optsInd,1);

opts(modeInd).filterType = options(optsInd,2);

opts(modeInd).testWindow = str2num(options(optsInd,3));

optStr = "_SS"+options(optsInd,1)+"_FILT"+options(optsInd,2);

data.(['fig',num2str(i)]).(modes(modeInd)+optStr).opts=opts(modeInd);

for noiseInd = 1:size(noise,2)

opts(modeInd).noiseSignal = pumpTheory_pub.interpolateNoise(1/(freq*60), noiseTime(1:length(rmmissing(noise(:,noiseInd)))), rmmissing(noise(:,noiseInd)));

if opts(modeInd).filterType=="fitting"

opts(modeInd).filterWindow = 0; opts(modeInd).downSample = opts(modeInd).fittingFrequency;

[~, ~, rawData] = pumpTheory_pub.outflow(modes(modeInd), restingIOP+stepSize, freq, opts(modeInd));

if isnan(rawData(end,1))

data.(['fig',num2str(i)]).(modes(modeInd)+optStr).times(1,noiseInd) = opts(modeInd).timeout;

else

data.(['fig',num2str(i)]).(modes(modeInd)+optStr).times(1,noiseInd) = rawData(end,1);

end

if verbose>=3, disp(" NoiseInd: "+noiseInd); end

else

for filtInd = 1:length(filtWindows)

opts(modeInd).filterWindow = filtWindows(filtInd);

opts(modeInd).downSample = 0;

[~, ~, rawData] = pumpTheory_pub.outflow(modes(modeInd), restingIOP+stepSize, freq, opts(modeInd));

if isnan(rawData(end,1))

data.(['fig',num2str(i)]).(modes(modeInd)+optStr).times(filtInd,noiseInd) = opts(modeInd).timeout;

else

data.(['fig',num2str(i)]).(modes(modeInd)+optStr).times(filtInd,noiseInd) = rawData(end,1);

end

end

if verbose>=3,

disp(" NoiseInd: "+noiseInd + ", filtInd: "+filtInd);

end

end

end

if verbose>=2,

disp(" All Noise | ModeInd: "+modeInd + ", OptsInd: "+optsInd);

end

end

%Add data to excel variables

[labels, strMatrix] = matrixWrite(labels, strMatrix, "Spacer", "FilterWidth", filtWindows);

for optsInd = 1:size(options,1)

optStr = "_SS"+options(optsInd,1)+"_FILT"+options(optsInd,2);

temp = data.(['fig',num2str(i)]).(modes(modeInd)+optStr);

[labels, strMatrix] = matrixWrite(labels, strMatrix, modes(modeInd)+optStr, temp.times(:,noiseExampleNum));

end

[labels, strMatrix] = matrixWrite(labels, strMatrix, "Spacer", "FilterWidth", filtWindows);

for optsInd = 1:size(options,1)

optStr = "_SS"+options(optsInd,1)+"_FILT"+options(optsInd,2);

temp = data.(['fig',num2str(i)]).(modes(modeInd)+optStr);

[labels, strMatrix] = matrixWrite(labels, strMatrix, "Avg_"+modes(modeInd)+optStr, mean(temp.times,2), "Std_"+modes(modeInd)+optStr, std(temp.times,0,2));

end

end

%plot the data if specified

if plott

figure; xWin = 60;

ax=subplot(5,5,1:4); plot(ax,noiseTime(1:length(noiseEx)), noiseEx);

xlabel("Time (min)"); ylabel("Pressure (mmHg)");

ylim([-2 10]);

subplot(5,5,5); barh(bin_mids, bin_Ns/(sum(bin_Ns))); hold on

plot(gauss_y,gauss_x);

ylim([-4 4]);

xlabel("Probability"); ylabel("Pressure (mmHg)");

subplot(5,1,2); plot(tempRaw(:,1), tempRaw(:,3), 'k', tempRaw(:,1), tempRaw(:,5), 'r', tempRaw(:,1), tempRaw(:,7), 'g'); hold on

plot([SSC_times(1)-5, SSC_times(1)], tempRaw(find(tempRaw(:,1)>=SSC_times(1),1),3)*[1,1], 'r','linewidth', 2)

plot([SSC_times(5)-5, SSC_times(5)], tempRaw(find(tempRaw(:,1)>=SSC_times(5),1),3)*[1,1], 'g','linewidth', 2)

xlim([0 xWin]); xlabel("Time (min)"); ylabel("Pressure (mmHg)");

subplot(5,1,3); plot(tempRaw(:,1), tempRaw(:,2), 'k', tempRaw(:,1), tempRaw(:,4), 'r', tempRaw(:,1), tempRaw(:,6), 'g'); hold on

plot([SSC_times(5)-5, SSC_times(5)], tempRaw(find(tempRaw(:,1)>=SSC_times(5),1),2)*[1,1], 'g','linewidth', 2)

xlim([0 xWin]); ylim([0 2.5]); xlabel("Time (min)"); ylabel("Flow (ul/min)");

subplot(5,1,4); semilogy(tempRaw(:,1), tempRaw(:,8), 'k', tempRaw(:,1), tempRaw(:,9), 'r', tempRaw(:,1), tempRaw(:,10), 'g'); hold on

ind = find(tempRaw(:,1)>=SSC_times(2),1);

semilogy([SSC_times(2)-5, SSC_times(2)], tempRaw(ind,8)*[1,1], 'b','linewidth', 2)

xlim([0 xWin]); xlabel("Time (min)"); ylabel("ratio (ul/min/mmHg)");

optColors = ['r'; 'b'; 'r'; 'b'; 'g'];

for modeInd = 1:length(modes)

subplot(5,length(modes),4*length(modes)+modeInd);

for optsInd = 1:size(options,1)

optStr = "_SS"+options(optsInd,1)+"_FILT"+options(optsInd,2);

temp = data.(['fig',num2str(i)]).(modes(modeInd)+optStr);

if options(optsInd,2)=="fitting"

plot(filtWindows([1,end]), [mean(temp.times,2),mean(temp.times,2)], optColors(optsInd));hold on

plot(filtWindows([1,end]), [temp.times(:,noiseExampleNum),temp.times(:,noiseExampleNum)], [optColors(optsInd),'.']);

else

plot(filtWindows, mean(temp.times,2), optColors(optsInd));hold on

plot(filtWindows, temp.times(:,noiseExampleNum), [optColors(optsInd),'.']);

end

end

xlabel("Filter Width"); ylabel("Settling Time (min)");

end

drawnow

end

%Save data to an excel output

strMatrix = [labels;strMatrix];

if save

writematrix(strMatrix, pumpTheory_pub.outputPath+"Figure_"+i+".xlsx");

end

case 9

if verbose>=1, disp("Figure "+i); end

%Constants/controls for figure

noiseExampleNum = 10;

nBin = 30;

%Declare variables needed for excel output

labels = [];

strMatrix = [];

%retrieve the noise signals to usee in th simulation

noise = readmatrix(pumpTheory_pub.awakeNoisePath);

noiseTime = noise(:,1);

noise = noise(:,2:end);

noiseEx = rmmissing(noise(:,noiseExampleNum));

%create a histogram of the a sample noise signal

[bin_Ns, bin_mids] = hist(noiseEx, nBin);

bin_Ns=bin_Ns'; bin_mids = bin_mids';

%Add to data Structure to be returned and to excel printable matrix as strings

data.(['fig',num2str(i)]).noiseTime = noiseTime;

data.(['fig',num2str(i)]).noiseData = noiseEx;

data.(['fig',num2str(i)]).noiseBins = bin_mids;

data.(['fig',num2str(i)]).noiseHist = bin_Ns;

[labels, strMatrix] = matrixWrite(labels, strMatrix, "Time", noiseTime, "Noise", noiseEx, "Spacer", "binCenters", bin_mids, "binCounts", bin_Ns);

if verbose>=2, disp(" Noise Hist Done"); end

%Create example traces for outflow facility measurement under each condition

%Set up the base settings for each mode

noiseEx = pumpTheory_pub.interpolateNoise(1/(freq*60), noiseTime, noise(:,noiseExampleNum));

CPg1_opts = pumpTheory_pub.createOutflowOptions("CPg1", "true99", 0, awakeFilterWidth.CPg1, "mean", noiseEx);

CPg2_opts = pumpTheory_pub.createOutflowOptions("CPg2", "true99", 0, awakeFilterWidth.CPg2, "mean", noiseEx);

CPp_opts = pumpTheory_pub.createOutflowOptions("CPp", "true99", 0, awakeFilterWidth.CPp, "mean", noiseEx);

CPpx_opts = pumpTheory_pub.createOutflowOptions("CPpx", "true99", 0, awakeFilterWidth.CPpx, "mean", noiseEx);

%Compile variables to allow for looping later

opts = [CPg1_opts; CPg2_opts; CPp_opts; CPpx_opts];

modes = ["CPg1"; "CPg2"; "CPp"; "CPpx"];

%Steady State Criteria Filter testWindow

options = ["ratio", "mean", testWin;

"ratio", "fitting", fittingTestWin;

"fitting_"+fittingTimeConst, "fitting", fittingTestWin];

%Cycle through different filter and Steady state criterias and create the example outflow facility measurement

for optsInd = 1:size(options,1)

for modeInd=1:length(modes)

%update the options for each iteration

opts(modeInd).steadyStateCriteria = options(optsInd,1);

opts(modeInd).filterType = options(optsInd,2);

opts(modeInd).testWindow = str2num(options(optsInd,3));

opts(modeInd).filterWindow = awakeFilterWidth.(modes(modeInd))*~contains(opts(modeInd).filterType,"fitting");

opts(modeInd).downSample = (options(optsInd,2)=="fitting")*opts(modeInd).fittingFrequency;

optStr = "_SS"+options(optsInd,1)+"_FILT"+options(optsInd,2);

data.(['fig',num2str(i)]).(modes(modeInd)+optStr).opts=opts(modeInd);

%run the simulation for the current options/mode

data.(['fig',num2str(i)]).(modes(modeInd)+optStr).outflow_raw, data.(['fig',num2str(i)]).(modes(modeInd)+optStr).raw] = pumpTheory_pub.outflow(modes(modeInd), setpoints, freq, opts(modeInd));

%Update the strMatrix variable

rawDataLabelTemp = ["Time", "Flow", "FiltFlow", "Ps", "FiltPs", "Setpoint"];

rawOutflowDataLabelTemp = ["Outflow_Ps", "Outflow_Flow", "Outflow_Pe", "Outflow_Ps_std", "Outflow_Flow_std", "Outflow_Pe_std"];

[labels, strMatrix] = matrixWrite(labels, strMatrix, "Spacer", modes(modeInd)+"_"+rawDataLabelTemp+optStr, data.(['fig',num2str(i)]).(modes(modeInd)+optStr).raw(:,[1,2,4,3,5,8]), modes(modeInd)+"_"+rawOutflowDataLabelTemp+optStr, data.(['fig',num2str(i)]).(modes(modeInd)+optStr).outflow_raw(:,[1,2,3,4,5,6]));

if verbose>=2,

disp(" Noise Example | OptsInd: "+optsInd + ", ModeInd: "+modeInd);

end

end

end

%Cycle through different filter and steady state conditions for each noise sample and compile results

%Reset the appropriate filter and SSC to cycle through

%Steady State Criteria Filter testWindow

options = ["window", "mean", testWin;

"ratio", "mean", testWin;

"window", "fitting", testWin;

"ratio", "fitting", testWin;

"fitting_"+fittingTimeConst, "fitting", fittingTestWin];

%Collecet the data

tempOutflows = [];

tempTimes = [];

for modeInd=1:length(modes)

tempLabels=[];

for optsInd = 1:size(options,1)

%update the options for each iteration

opts(modeInd).steadyStateCriteria = options(optsInd,1);

opts(modeInd).filterType = options(optsInd,2);

opts(modeInd).testWindow = str2num(options(optsInd,3));

opts(modeInd).filterWindow = awakeFilterWidth.(modes(modeInd))*~contains(opts(modeInd).filterType,"fitting");

opts(modeInd).downSample = (options(optsInd,2)=="fitting")*opts(modeInd).fittingFrequency;

data.(['fig',num2str(i)]).(modes(modeInd)).opts=opts(modeInd);

%run the simulation for the current options/mode

for noiseInd = 1:size(noise,2)

opts(modeInd).noiseSignal = pumpTheory_pub.interpolateNoise(1/(freq*60), noiseTime, noise(:,noiseInd));

[data.(['fig',num2str(i)]).(modes(modeInd)).outflows(noiseInd,optsInd), ~, temp] = pumpTheory_pub.outflow(modes(modeInd), setpoints, freq, opts(modeInd));

data.(['fig',num2str(i)]).(modes(modeInd)).times(noiseInd,optsInd) = temp(end,1);

if verbose>=3, disp(" NoiseInd: "+noiseInd); end

end

tempOutflows((1:size(noise,2))+size(noise,2)*(optsInd-1),modeInd) = data.(['fig',num2str(i)]).(modes(modeInd)).outflows(:,optsInd);

tempTimes((1:size(noise,2))+size(noise,2)*(optsInd-1),modeInd) = data.(['fig',num2str(i)]).(modes(modeInd)).times(:,optsInd);

%Update the label variables

tempLabels = [tempLabels; repmat("SS."+options(optsInd,1)+"_FILT."+options(optsInd,2),size(noise,2),1)];

if verbose>=2,

disp(" All Noise | ModeInd: "+modeInd + ", OptsInd: "+optsInd);

end

end

end

[labels, strMatrix] = matrixWrite(labels, strMatrix, "Spacer", "Condition", tempLabels, modes', tempOutflows, "Spacer", "Condition", tempLabels, modes', tempTimes);

%plot the data if specified

if plott

figure;

exampleModeStr = "_SSratio_FILTfitting";

x={};

optStrs = "_"+join(replace(options(:,2:-1:1),"ting",""),"_");

temp = nan(size(noise,2),1); %spaceholder for the plot

facilities = [];

times = [];

for modeInd=1:length(modes)

%isolate example data

exData = data.(['fig',num2str(i)]).(modes(modeInd)+exampleModeStr);

%plot the raw traces

subplot(3,4,modeInd);

plot(exData.raw(:,1),exData.raw(:,3),'k',exData.raw(:,1),exData.raw(:,5),'r');

xlabel("Time (min)"); ylabel("Pressure (mmHg)");

subplot(3,4,modeInd+4);

plot(exData.raw(:,1),exData.raw(:,2),'k',exData.raw(:,1),exData.raw(:,4),'r');

xlabel("Time (min)"); ylabel("Flow (ul/min)");

%plot the outflow linear estimates

p = [10,20,30,40,45];

subplot(3,6,modeInd+13);

errorbar(exData.outflow_raw(:,1),exData.outflow_raw(:,2),

exData.outflow_raw(:,5),exData.outflow_raw(:,5),exData.outflow_raw(:,4),exData.outflow_raw(:,4),'o');

fitVal = fitlm(exData.outflow_raw(:,1),exData.outflow_raw(:,2));

m = table2array(fitVal.Coefficients(2,1));

b = table2array(fitVal.Coefficients(1,1));

hold on; plot(p, m*p+b);

xlabel("Pressure (mmHg)"); ylabel("Flow (ul/min)");

%Set up summary plots

x(1+(modeInd-1)*(length(optStrs)+1)) = cellstr(repmat('.',1,modeInd));

x((1:length(optStrs))+1+(modeInd-1)*(length(optStrs)+1)) = cellstr(modes(modeInd)+optStrs);

facilities = [facilities, temp, data.(['fig',num2str(i)]).(modes(modeInd)).outflows];

times = [times, temp, data.(['fig',num2str(i)]).(modes(modeInd)).times];

end

x(1+length(modes)*(length(optStrs)+1)) = cellstr(repmat('.',1,length(modes)+1));

times = [times, temp];

facilities = [facilities, temp];

facilities(facilities>0.1|facilities<0)=NaN;

%plot the summary bar plots for facility and times

subplot(3,6,13);

plotbar(x,times);

hold on; plot([1:size(times,2)], times,'.');

ylabel("Times (min)");

subplot(3,6,18);

plotbar(x,facilities);

hold on; plot([1:size(facilities,2)], facilities,'.');

ylabel("Facilities (ul/min/mmHg)");

drawnow

end

%Save data to an excel output

strMatrix = [labels;strMatrix];

if save

writematrix(strMatrix, pumpTheory_pub.outputPath+"Figure_"+i+".xlsx");

end

case 10

%Effect of step size on settle time and measurement uncertainty for awake noise

if verbose>=1, disp("Figure "+i); end

%Constants/controls for figure

noiseExampleNum = 11;

nBin = 30;

stepSizes = [0.5;1;2;3;4;5];

nSteps = 7;

%Declare variables needed for excel output

labels = [];

strMatrix = [];

%retrieve the noise signals to usee in th simulation

noise = readmatrix(pumpTheory_pub.awakeNoisePath);

noiseTime = noise(:,1);

noise = noise(:,2:end);

noiseEx = rmmissing(noise(:,noiseExampleNum));

%Create example traces for outflow facility measurement under each condition

%Set up the base settings for each mode

noiseEx = pumpTheory_pub.interpolateNoise(1/(freq*60), noiseTime, noise(:,noiseExampleNum));

CPg1_opts = pumpTheory_pub.createOutflowOptions("CPg2", "true99", 0, awakeFilterWidth.CPg1, "mean", noiseEx);

CPg2_opts = pumpTheory_pub.createOutflowOptions("CPg2", "true99", 0, awakeFilterWidth.CPg2, "mean", noiseEx);

CPp_opts = pumpTheory_pub.createOutflowOptions("CPp", "true99", 0, awakeFilterWidth.CPp, "mean", noiseEx);

CPpx_opts = pumpTheory_pub.createOutflowOptions("CPpx", "true99", 0, awakeFilterWidth.CPpx, "mean", noiseEx);

%Compile variables to allow for looping later

opts = [CPg1_opts; CPg2_opts; CPp_opts; CPpx_opts];

modes = ["CPg1"; "CPg2"; "CPp"; "CPpx"];

runInds = 1;

opts=opts(runInds);

modes=modes(runInds);

%Steady State Criteria Filter testWindow

options = ["fitting_"+fittingTimeConst, "fitting", fittingTestWin];

%Cycle through different filter and Steady state criterias and create example outflow facility measurement

for optsInd = 1:size(options,1)

for modeInd=1:length(modes)

%update the options for each iteration

opts(modeInd).steadyStateCriteria = options(optsInd,1);

opts(modeInd).filterType = options(optsInd,2);

opts(modeInd).testWindow = str2num(options(optsInd,3));

opts(modeInd).filterWindow = awakeFilterWidth.(modes(modeInd))*~contains(opts(modeInd).filterType,"fitting");

opts(modeInd).downSample = (options(optsInd,2)=="fitting")*opts(modeInd).fittingFrequency;

for stepInd = 1:length(stepSizes)

customSetpoints = restingIOP+stepSizes(stepInd).*(1:nSteps);

optStr = "_stepSize"+replace(string(stepSizes(stepInd)),'.','_');

data.(['fig',num2str(i)]).(modes(modeInd)+optStr).opts=opts(modeInd);

%run the simulation for the current options/mode

[~, data.(['fig',num2str(i)]).(modes(modeInd)+optStr).outflow_raw, data.(['fig',num2str(i)]).(modes(modeInd)+optStr).raw] = pumpTheory_pub.outflow(modes(modeInd), customSetpoints, freq, opts(modeInd));

%Update the strMatrix variable

rawDataLabelTemp = ["Time", "Flow", "FiltFlow", "Ps", "FiltPs", "Setpoint"];

rawOutflowDataLabelTemp = ["Outflow_Ps", "Outflow_Flow", "Outflow_Pe", "Outflow_Ps_std", "Outflow_Flow_std", "Outflow_Pe_std"];

[labels, strMatrix] = matrixWrite(labels, strMatrix, "Spacer", modes(modeInd)+"_"+rawDataLabelTemp+optStr, data.(['fig',num2str(i)]).(modes(modeInd)+optStr).raw(:,[1,2,4,3,5,8]), modes(modeInd)+"_"+rawOutflowDataLabelTemp+optStr, data.(['fig',num2str(i)]).(modes(modeInd)+optStr).outflow_raw(:,[1,2,3,4,5,6]));

if verbose>=2,

disp(" Noise Example | OptsInd: "+optsInd + ", ModeInd: "+modeInd + ", StepInd: "+stepInd);

end

end

end

end

%Cycle through different filter and steady state conditions for each noise sample and compile results

%Reset the appropriate filter and SSC to cycle through

%Steady State Criteria Filter testWindow

options = ["window", "mean", testWin;

"ratio", "mean", testWin;

"window", "fitting", testWin;

"ratio", "fitting", testWin;

"fitting_"+fittingTimeConst, "fitting", fittingTestWin];

%Collect the data

tempOutflows = [];

tempTimes = [];

for modeInd=1:length(modes)

tempLabels=[];

for optsInd = 1:size(options,1)

%update the options for each iteration

opts(modeInd).steadyStateCriteria = options(optsInd,1);

opts(modeInd).filterType = options(optsInd,2);

opts(modeInd).testWindow = str2num(options(optsInd,3));

opts(modeInd).filterWindow = awakeFilterWidth.(modes(modeInd))*~contains(opts(modeInd).filterType,"fitting");

opts(modeInd).downSample = (options(optsInd,2)=="fitting")*opts(modeInd).fittingFrequency;

structLabel = modes(modeInd)+"_SS"+options(optsInd,1)+"_FILT"+options(optsInd,2);

data.(['fig',num2str(i)]).(structLabel).opts=opts(modeInd);

%run the simulation for the current options/mode

for noiseInd = 1:size(noise,2)

opts(modeInd).noiseSignal = pumpTheory_pub.interpolateNoise(1/(freq*60), noiseTime, noise(:,noiseInd));

%Run simulation for various nSteps and stepSizes

for stepInd = 1:length(stepSizes)

customSetpoints = restingIOP+stepSizes(stepInd).*(1:nSteps);

[~, outflowTemp, temp] = pumpTheory_pub.outflow(modes(modeInd), customSetpoints, freq, opts(modeInd));

%Calculate Facility and times for various nSteps

for nInd = 3:nSteps

outflowFit = fitlm(outflowTemp(1:nInd,1), outflowTemp(1:nInd,2));

outflow = table2array(outflowFit.Coefficients(2,1));

time = temp(find(temp(:,8)==customSetpoints(nInd),1,'last'),1);

data.(['fig',num2str(i)]).(structLabel).outflows(noiseInd,stepInd,nInd-2) = outflow;

data.(['fig',num2str(i)]).(structLabel).times(noiseInd,stepInd,nInd-2) = time;

data.(['fig',num2str(i)]).(structLabel).conditions(noiseInd,stepInd,nInd-2) = "_stepSize"+stepSizes(stepInd)+"_numSteps"+nInd;

if verbose>=3,

disp(" NoiseInd: " + noiseInd + ", StepInd: " + stepInd + ", nInd: " + nInd);

end

end

end

if verbose>=2,

disp(" All Noise | ModeInd: "+modeInd + ", OptsInd: "+optsInd); end

end

end

end

%Print out the summary data for each mode and option

outflowTemp = [];

for modeInd=1:length(modes)

tempLabels=[];

for optsInd = 1:size(options,1)

%update the options for each iteration

opts(modeInd).steadyStateCriteria = options(optsInd,1);

opts(modeInd).filterType = options(optsInd,2);

opts(modeInd).testWindow = str2num(options(optsInd,3));

opts(modeInd).filterWindow = awakeFilterWidth.(modes(modeInd))*~contains(opts(modeInd).filterType,"fitting");

opts(modeInd).downSample = (options(optsInd,2)=="fitting")*opts(modeInd).fittingFrequency;

structLabel = modes(modeInd)+"_SS"+options(optsInd,1)+"_FILT"+options(optsInd,2);

xLabels = string(3:nSteps);

yLabels = ["[StepSize \ NumSteps]_"+structLabel;string(stepSizes)];

outflowTemp = xLabels;

outflowTemp((1:length(stepSizes))+1,1:nSteps-2) = mean(data.(['fig',num2str(i)]).(structLabel).outflows,1);

outflowTemp(size(outflowTemp,1)+(1:2),1:nSteps-2) = nan;

outflowTemp(size(outflowTemp,1)+1,1:nSteps-2) = "Std";

outflowTemp(size(outflowTemp,1)+1,1:nSteps-2) = xLabels;

outflowTemp((1:length(stepSizes))+size(outflowTemp,1),1:nSteps-2) = std(data.(['fig',num2str(i)]).(structLabel).outflows,0,1);

timeTemp = xLabels;

timeTemp((1:length(stepSizes))+1,1:nSteps-2) = mean(data.(['fig',num2str(i)]).(structLabel).times,1);

timeTemp(size(timeTemp,1)+(1:2),1:nSteps-2) = nan;

timeTemp(size(timeTemp,1)+1,1:nSteps-2) = "Std";

timeTemp(size(timeTemp,1)+1,1:nSteps-2) = xLabels;

timeTemp((1:length(stepSizes))+size(timeTemp,1),1:nSteps-2) = std(data.(['fig',num2str(i)]).(structLabel).times,0,1);

yLabels = [yLabels;nan;nan;nan;yLabels];

[labels, strMatrix] = matrixWrite(labels, strMatrix, "Spacer", "Mean_"+structLabel, [yLabels,outflowTemp], "Spacer", "Mean_"+structLabel, [yLabels,timeTemp]);

end

[labels, strMatrix] = matrixWrite(labels, strMatrix, "Spacer", "Spacer", "Spacer");

end

%plot the data if specified

if plott

figure;

exampleModeStr = modes(1);

for stepInd=1:length(stepSizes)

%isolate example data

exData = data.(['fig',num2str(i)]).(exampleModeStr+"_stepSize"+replace(string(stepSizes(stepInd)),'.','_'));

%plot the raw traces

subplot(6,length(stepSizes),stepInd);

plot(exData.raw(:,1),exData.raw(:,3),'k',exData.raw(:,1),exData.raw(:,5),'r');

xlabel("Time (min)"); ylabel("Pressure (mmHg)");

subplot(6,length(stepSizes),stepInd+length(stepSizes));

plot(exData.raw(:,1),exData.raw(:,2),'k',exData.raw(:,1),exData.raw(:,4),'r');

xlabel("Time (min)"); ylabel("Flow (ul/min)");

%plot the outflow linear estimates

p = [10,20,30,40,45];

subplot(6,length(stepSizes),stepInd+length(stepSizes)*2);hold on

colors = ['k';'r';'g'; 'b'; 'm'];

for nInd = nSteps:-1:3

errorbar(exData.outflow_raw(1:nInd,1),exData.outflow_raw(1:nInd,2)

,exData.outflow_raw(1:nInd,5),exData.outflow_raw(1:nInd,5),exData.outflow_raw(1:nInd,4),exData.outflow_raw(1:nInd,4),['o', colors(nInd-2)]);

fitVal = fitlm(exData.outflow_raw(1:nInd,1),exData.outflow_raw(1:nInd,2));

m = table2array(fitVal.Coefficients(2,1));

b = table2array(fitVal.Coefficients(1,1));

plot(p, m*p+b, colors(nInd-2));

end

xlim([15 15+stepInd*5]);

ylim([0 1]);

xlabel("Pressure (mmHg)"); ylabel("Flow (ul/min)");

end

%plot the summary bar plots for facility and times

for optsInd = 1:size(options,1)

structLabel = modes(1)+"_SS"+options(optsInd,1)+"_FILT"+options(optsInd,2);

barData = data.(['fig',num2str(i)]).(structLabel);

subplot(4,5,optsInd+10);

plotbar3(stepSizes, 3:nSteps, barData.outflows);

zlim([0 0.05]);

ylabel("Step Size (mmHg)"); xlabel("Num Steps"); zlabel("Outflow (ul/min/mmHg)");

subplot(4,5,optsInd+15);

plotbar3(stepSizes, 3:nSteps, barData.times);

zlim([0 500]);

ylabel("Step Size (mmHg)"); xlabel("Num Steps"); zlabel("Settling Times (min)");

end

drawnow

end

%Save data to an excel output

strMatrix = [labels;strMatrix];

if save

writematrix(strMatrix, pumpTheory_pub.outputPath+"Figure_"+i+".xlsx");

end

otherwise

warning(['figure ',num2str(i),' does not exist']);

end

end

%Helper functions for creating an exportable excel matrix

function [labels, masterStr] = matrixWrite(labels, masterStr, varargin)

%Wrapper function to make concatStrs easier to use as a function and more compact

ind_varargin = 1;

if isempty(labels)&&nargin>3

while lower(varargin{ind_varargin})=="spacer"

labels = [labels, ""];

masterStr = concatStrs(masterStr, "");

ind_varargin=ind_varargin+1;

end

labels = string(varargin{ind_varargin});

masterStr = string(varargin{ind_varargin+1});

%If number of columns dont match, fill with blank titles and columns as neccessary to restore synchronization

for n=1:size(string(varargin{ind_varargin+1}),2)-size(string(varargin{ind_varargin}),2)

labels=[labels,""];

end

for n=1:size(string(varargin{ind_varargin}),2)-size(string(varargin{ind_varargin+1}),2)

masterStr = concatStrs(masterStr, "");

end

if nargin-2 > ind_varargin+1, ind_varargin=ind_varargin+2; end

end

while ind_varargin<=nargin-2

%Add in an empty column when specified

if lower(varargin{ind_varargin})=="spacer"

labels = [labels, ""];

masterStr = concatStrs(masterStr, "");

ind_varargin=ind_varargin+1;

else

%Update the label and masteerStr for each pair of label and variables

labels = [labels, string(varargin{ind_varargin})];

masterStr = concatStrs(masterStr, string(varargin{ind_varargin+1}));

%If number of columns dont match,fill with blank titles and columns as necessary to restore synchronization

for n=1:size(string(varargin{ind_varargin+1}),2)-size(string(varargin{ind_varargin}),2)

labels=[labels,""];

end

for n=1:size(string(varargin{ind_varargin}),2)-size(string(varargin{ind_varargin+1}),2)

masterStr = concatStrs(masterStr, "");

end

ind_varargin=ind_varargin+2;

end

end

end

function ans = concatStrs(txt1, txt2)

%will pad txt1 or txt2 to ensuree same vertical length before horizontally combining

if isempty(txt2)

txt2 = strings(length(txt1(:,1)),1);

end

n1=size(txt1,1); n2 = size(txt2,1);

if n1<n2

txt1 = padstr(txt1,n2-n1,"");

else

txt2 = padstr(txt2,n1-n2,"");

end

ans = [txt1, txt2];

end

function paddedTxt = padstr(text,padlen,txt)

%pads a variable along the last dimension with the txt argument provided

paddedTxt = strings(size(text)+padarray(padlen,ndims(text)-1,0,'post')')+txt;

logInd = true(size(text));

logInd = padarray(logInd,padarray(padlen,ndims(text)-1,0,'post')',false,'post');

paddedTxt(logInd)=text;

end

function plotbar(x,y)

%Plots a bar plot with error bars

y=y';

x = reordercats(categorical(x),x);

bar(x,nanmean(y,ndims(y)));

hold on

er = errorbar(x,nanmean(y,ndims(y)),nanstd(y,0,ndims(y)),nanstd(y,0,ndims(y)));

for ii=1:length(er)

er(ii).Color = [0 0 0];

er(ii).LineStyle = 'none';

end

end

function plotbar3(x,y,z)

%Create 3d barplot with error bars

meanZ(1:length(x), 1:length(y)) = mean(z,1);

stdZ(1:length(x), 1:length(y)) = std(z,0,1);

bar3(meanZ); hold on;

hold on

bar3(meanZ+stdZ, 0.03, 'k');

set(gca,'XTickLabel',y)

set(gca,'YTickLabel',x)

end

function [PsOut, PsCleanOut] = eyeSim(time, Fs, Ps, PsClean, noiseSignal, noiseStd, dt, restingIOP)

% This function utillizes a runge kutta method to estimate

% pressre response to provided flow pattern.

%

% time: vector of time (default is seconds)

% Fs: vector of the same length of time with the flow pattern

% Ps: vector of known pressure values (should be smaller in length than time and Fs)

% PsClean: Ps vector without the noise incorpereated.

% noiseSignal: optional vector of desired additive noise, time step assumed to match 'time' units

% noiseStd: optional paramater to incorpereate gaussian noise of the specified std.

% dt is inferred from time, unless specified here

% restingIOP defaults to 15, unless specified otherwise here.

if nargin < 4

error("not enough input arguments");

end

if

(length(time)<=length(PsClean))&&(length(Fs)<=length(PsClean))&&(length(PsClean)>=2)&&lngth(time)~=length(Fs)

error("Ps vector must have at least 2 data points. time and Fs vectors must be greater in length than the Ps vector and the sam length.");

end

if nargin<5, noiseSignal = []; end;

if nargin<6, noiseStd = 0; end;

if nargin<7

if length(time)>2

dt = (time(end)-time(end-1)); %units of mins

else

error('dt was not included and could not be infered from one or less time data points');

end

end

if nargin<8, restingIOP = 15; end;

mergeTime = 0.5; %min. Time to smooth signal when the simulation runs longer than the provided noise signal so there is no artificial large step in the noise signal when looping back

%extract initial condition to prevent copying entire array each time function called

PsOut = Ps(end-2:end);

PsCleanOut = PsClean(end-2:end);

%Recursively fill in each point on at a time if needed

N = length(time) - length(PsClean);

if N>1

[PsOut(end+1:end+N), PsCleanOut(end+1:end+N)] = pumpTheory_pub.eyeSim(time(1:end-1), Fs(1:end-1), Ps, PsClean, nois_signal, noiseStd, dt, restingIOP);

end

%%Runge Kutta 4th order:

%constants and their values

h=dt;

rc = pumpTheory_pub.rc;

cs = pumpTheory_pub.cs;

rt = pumpTheory_pub.rt;

cw1 = pumpTheory_pub.cw1;

cw2 = pumpTheory_pub.cw2;

rw = pumpTheory_pub.rw;

A = rt*rw*cw1*cw2;

B = rc*rw*(cw1+cw2)+rc*rt*cw2+rt*rw*(cw1+cw2);

G = rc*cs+rt*cs+rw*(cw1+cw2)+rt*cw2;

%ODE for pressure as a function of flow given A=rt*rw*cw1*cw2; B=rc*rw*(cw1+cw2)+rc*rt*cw2+rt*rw*(cw1+cw2); G=rc*cs+rt*cs+rw*(cw1+cw2)+rt*cw2;

% Ps'''+((A+Bcs)/(A*rc*cs))Ps''+(G/(A*rc*cs))Ps'+(1/(A*rc*cs))Ps = (A*rc/(A*rc*cs))Fs''+(B/(A*rc*cs))Fs'+((rc+rt)/(A*rc*cs))Fs+(1/(A*rc*cs))restingIOP

%

% Given x=Fs, y=Ps, z1=y', and z2=y'', we can write

%

% 1) y' = z1

% 2) z1' = z2

% 2) z2' = -((A+Bcs)/(A*rc*cs))z2-(G/(A*rc*cs))z1-(1/(A*rc*cs))y = (A*rc/(A*rc*cs))Fs''+(B/(A*rc*cs))Fs'+((rc+rt)/(A*rc*cs))Fs+(1/(A*rc*cs))restingIOP

function z2_prime = F2(x, x_prime, x_dprime, y, z1, z2)

z2_prime = -((A+B*cs)/(A*rc*cs))*z2-(G/(A*rc*cs))*z1-(1/(A*rc*cs))*y + (A*rc/(A*rc*cs))*x_dprime+(B/(A*rc*cs))*x_prime+((rc+rt)/(A*rc*cs))*x+(1/(A*rc*cs))*restingIOP;

end

dy1 = diff(PsCleanOut(end-1:end))/diff(time(end-1:end));

ddy1 = (diff(PsCleanOut(end-1:end))/diff(time(end-1:end))-diff(PsCleanOut(end-2:end-1))/diff(time(end-2:end-1)))/(diff(time([end-2,end]))/2);

dx2 = diff(Fs(end-1:end))/diff(time(end-1:end));

dx1 = diff(Fs(end-2:end-1))/diff(time(end-2:end-1));

ddx2 = (diff(Fs(end-1:end))/diff(time(end-1:end))-diff(Fs(end-2:end-1))/diff(time(end-2:end-1)))/(diff(time([end-2,end]))/2);

ddx1 = (diff(Fs(end-2:end-1))/diff(time(end-2:end-1))-diff(Fs(end-3:end-2))/diff(time(end-3:end-2)))/(diff(time([end-3,end-1]))/2);

k1y = dy1;

k1z1 = ddy1;

k1z2 = F2(Fs(end-1), dx1, ddx1, PsCleanOut(end), dy1, ddy1);

k2y = k1y + (h/2)*k1z1;

k2z1 = k1z1 + (h/2)*k1z2;

k2z2 = F2((Fs(end-1)+Fs(end))/2, (dx1+dx2)/2, (ddx1+ddx2)/2, PsCleanOut(end)+(h/2)*k1y, k2y, k2z1);

k3y = k2y + (h/2)*k2z1;

k3z1 = k2z1 + (h/2)*k2z2;

k3z2 = F2((Fs(end-1)+Fs(end))/2, (dx1+dx2)/2, (ddx1+ddx2)/2, PsCleanOut(end)+(h/2)*k2y, k3y, k3z1);

k4y = k3y + h*k3z1;

k4z1 = k3z1 + h*k3z2;

k4z2 = F2(Fs(end), dx2, ddx2, PsCleanOut(end)+h*k3y, k4y, k4z1);

kz2_bar = (k1z2+2*k2z2+2*k3z2+k4z2)/6;

kz1_bar = k1z1+kz2_bar*h;

ky_bar = dy1+kz1_bar*h;

PsCleanOut(end+1) = PsCleanOut(end)+ky_bar*h;

%ODE for pressure as a function of flow given A = rc*rw*cw+rc*rt*cw+rt*rw*cw and B = rc*cs+rt*cs+rw*cw+rt*cw;:

% Ps'' + (B/(A*cs))Ps' + (1/(A*cs))Ps = (1/cs)*Fs' + ((rc+rt)/(A*cs))Fs + (1/(A*cs))RestingIOP

% Given x=Fs, y=Ps, and z=y', we can write

% 1) y' = z

% 2) z' = -(y_prime*B)/(A*cs) - y/(A*cs) + x_prime/cs + x*((rc+rt)/(A*cs)) + restingIOP/(A*cs)

%

% function z_prime = F2(x, x_prime, y, y_prime)

% z_prime = -(y_prime*B)/(A*cs) - y/(A*cs) + x_prime/cs + x*((rc+rt)/(A*cs)) + restingIOP/(A*cs);

% end

%

% dy1 = diff(PsCleanOut(end-1:end))/diff(time(end-1:end));

% dx2 = diff(Fs(end-1:end))/diff(time(end-1:end));

% dx1 = diff(Fs(end-2:end-1))/diff(time(end-2:end-1));

% k1y = dy1;

% k1z = F2(Fs(end-1), dx1, PsCleanOut(end), dy1);

% k2y = k1y + (h/2)*k1z;

% k2z = F2((Fs(end-1)+Fs(end))/2, (dx1+dx2)/2, PsCleanOut(end)+(h/2)*k1y, k2y);

% k3y = k2y + (h/2)*k2z;

% k3z = F2((Fs(end-1)+Fs(end))/2, (dx1+dx2)/2, PsCleanOut(end)+(h/2)*k2y, k3y);

% k4y = k3y + h*k3z;

% k4z = F2(Fs(end), dx2, PsCleanOut(end)+h*k3y, k4y);

% kz_bar = (k1z+2*k2z+2*k3z+k4z)/6;

% ky_bar = dy1+kz_bar*h;

% PsCleanOut(end+1) = PsCleanOut(end)+ky_bar*h;

PsOut(end+1) = PsCleanOut(end)+normrnd(0,noiseStd);

if ~isempty(noiseSignal)

noiseTimeEnd=(length(noiseSignal)-1)*dt;

if (time(end)>mergeTime)&&(abs(time(end)-noiseTimeEnd*round(time(end)/noiseTimeEnd))<=mergeTime) %Apply sigmoid function around the transition when looping the noise

noiseVal=(1/(1+exp(-(abs(time(end)-noiseTimeEnd*round(time(end)/noiseTimeEnd))-mergeTime/2)*14/mergeTime)))*(noiseSignal(mod(length(time)-3,length(noiseSignal))+1) - noiseSignal(1));

else

noiseVal=noiseSignal(mod(length(time)-3,length(noiseSignal))+1) - noiseSignal(1);

end

PsOut(end) = PsOut(end)+noiseVal;

end

%Remove initial conditions

PsOut = PsOut(4:end);

PsCleanOut = PsCleanOut(4:end);

end

function [outflow, outflowData, rawData] = outflow(mode, setpoints, freq, opts)

% Simulate a full outflow Facility Measurement

%

% Mode: string specifying facility measurement technique (CF, CPg1, CPg2, CPp, CPpx)

% stepoints: specifies the pressure (mmHg) targets for each model, or the flow rates (ul/min) in the 'CF' case

% freq: sampling/simulation frequency in Hz

% Opts: struct with more settings to control such as filter and test windows

if nargin < 3, error("Not enough input argumments."); end

if nargin < 4, opts = pumpTheory_pub.createOutflowOptions(mode); end

if (opts.steadyStateCriteria=="true99")&&((~isempty(opts.noiseSignal))||(opts.noiseStd>0))

error("Cannot use 'true99' as the stop criteria when there is noise being added to the signal.");

end

%Apply default values for testWindow if steadyStateCriteria is applied and testWindow is not

if opts.testWindow==0

opts.testWindow = 5*(opts.steadyStateCriteria=="window")+1*(opts.steadyStateCriteria=="ratio")+3*(opts.steadyStateCriteria=="fitting")+0;

end

%Check for discrpeency between filterWindow and fitting frequency

if mod(opts.filterWindow, 1/(opts.fittingFrequency*60))~=0

error('The fitting window is not a factor of the filterwindow, thus there will be a short period after the filter window before the first point is fitted')

end

%If downsample freq if not valid, give warning and fix if possible

if (opts.downSample >= freq)||(opts.downSample <= 0)

if opts.downSample > freq

warning('downSample set to nan since the downSample frequeency was higher than the simulation freq');

end

opts.downSample = nan;

else

downSampleIndex = freq/opts.downSample;

if ~isinteger(downSampleIndex)

downSampleIndex=ceil(downSampleIndex);

opts.downSample = freq/downSampleIndex;

warning("Down Sample freq was not a factor of the simulation frequency, a downSample freq of "+freq/downSampleIndex+" was used instead");

end

end

%If no postFiltering and mode is CPpx, adjust filterwidth to ensur correct width

if (~pumpTheory_pub.CPpx_postFiltering)&&(mode=='CPpx')

opts.filterWindow=pumpTheory_pub.CPpxFiltWin;

end

dt = 1/(freq*60); %Convert to period in minutes

%Take car of custom k for CPp/CPpx models

CPpx_filtWidth = pumpTheory_pub.filtWinCPpx;

if contains(lower(mode),"custom")

if isfield(opts, 'customK')

flowEq = pumpTheory_pub.getFlowEq(mode, opts.customK);

end

if isfield(opts, 'customCPpFilt')

CPpx_filtWidth = customCPpFilt;

end

else

flowEq = pumpTheory_pub.getFlowEq(mode);

end

% Raw Variables, initiallized with steady state at resting

time = [-2;-1;0]*dt;

flow = [0;0;0];

Ps = [1;1;1]*opts.restingIOP;

PsClean = Ps;

Pe = Ps;

target = Ps;

meanPs = Ps;

meanFlow = flow;

f_p = meanFlow./meanPs;

% Declare variables for measurment

outflowData = nan(length(setpoints),6);

rawOutflowData = [];

setpoint = opts.restingIOP;

if mode=="CF", setpoint = 0; end

opts.dt = dt;

for i = 1:length(setpoints)

%Reset simulation paramaters

lastSetpoint = setpoint;

setpoint = setpoints(i);

SSCounter = 0;

[opts.SSPs, opts.SSflow] = pumpTheory_pub.getSteadyState(mode, setpoint, opts);

[opts.SSPs_old, opts.SSflow_old] = pumpTheory_pub.getSteadyState(mode, lastSetpoint, opts);

fitParams = struct('Ps', [setpoint-lastSetpoint,5,0], 'flow', [nan,nan,nan], 'lastPs', nan, 'lastFlow', nan);

stopFlag = false;

minTimeBeforeCheck = (time(end)+opts.testWindow+opts.filterWindow)*((opts.steadyStateCriteria~="time")&&(opts.steadyStateCriteria~="true99")); %0 if criteria is time or true99, else computed

%Declare variables for fitting downsampled data

if ~isnan(opts.downSample)

timeDS = [time(end)];

PsDS = [Ps(end)];

flowDS = [flow(end)];

SSCounterDS = 0;

end

%Simulate each setpoint until steady state is detected or timeout limit is reached

while ~stopFlag&&(SSCounter*dt<opts.timeout)

%Get next Flow Rate

SSCounter = SSCounter+1;

time(end+1) = time(end)+dt;

flow(end+1) = flowEq(setpoint, Ps(end), mean(Ps(max(end-SSCounter,end-round(CPpx_filtWidth/opts.dt)):end)));

%Update Simulation

[Ps(end+1), PsClean(end+1)] = pumpTheory_pub.eyeSim(time, flow, Ps, PsClean, opts.noiseSignal, opts.noiseStd, dt, opts.restingIOP);

Pe(end+1) = PsClean(end)-pumpTheory_pub.rc*(flow(end)-pumpTheory_pub.cs*(Ps(end)-Ps(end-1))/dt);

target(end+1) = setpoint;

%Apply filtering based on movAvg vs fitting, trueMovAvg vs not, and also apply to flow if dictated

if isnan(opts.downSample)

[meanPs(end+1), meanFlow(end+1), fitParams] = pumpTheory_pub.filterSignals(time, Ps, flow, SSCounter, opts, fitParams);

else %Apply downsampling then filtering if dictated

if mod(SSCounter,downSampleIndex)==0

SSCounterDS = SSCounterDS+1;

timeDS(SSCounterDS,1) = mean(time(end-downSampleIndex+1:end));

PsDS(SSCounterDS,1) = mean(Ps(end-downSampleIndex+1:end));

flowDS(SSCounterDS,1) = mean(flow(end-downSampleIndex+1:end));

end

if ((opts.filterType == "fitting")&&(SSCounter < round(pumpTheory_pub.minTimeBeforeFit/opts.dt)))

[meanPs(end+1), meanFlow(end+1), fitParams] = pumpTheory_pub.filterSignals(time, Ps, flow, SSCounter, opts, fitParams);

else

[meanPs(end+1), meanFlow(end+1), fitParams] = pumpTheory_pub.filterSignals(timeDS, PsDS, flowDS, SSCounter, opts, fitParams);

end

end

if isnan(Ps(end))||isnan(flow(end))||isnan(meanPs(end))||isnan(meanFlow(end))

break;

end

%Check for steady state

stepSize = (setpoint-lastSetpoint)*((mode=="CF")*pumpTheory_pub.rt+(mode~="CF"));

if time(end)>=minTimeBeforeCheck

[stopFlag, minTimeBeforeCheck] = pumpTheory_pub.checkSteadyState(time, meanPs, meanFlow, stepSize, SSCounter, opts, [fitParams.Ps(2),fitParams.flow(2)]);

end

end

if isnan(Ps(end))||isnan(flow(end))||isnan(meanPs(end))||isnan(meanFlow(end))

break;

end

%Update summary variables

if SSCounter*dt < opts.timeout

SSWin = fix(opts.testWindow/dt);

if contains(opts.steadyStateCriteria,"fitting")

outflowData(i,:) = [meanPs(end),meanFlow(end),Pe(end),std(meanPs(end-SSWin:end)),std(meanFlow(end-SSWin:end)),std(Pe(end-SSWin:end))];

else

outflowData(i,:) = [mean(meanPs(end-SSWin:end)),mean(meanFlow(end-SSWin:end)),mean(Pe(end-SSWin:end)),std(meanPs(end-SSWin:end)),std(meanFlow(end-SSWin:end)),std(Pe(end-SSWin:end))];

end

end

%trim filtered data to avoid artifacts

if pumpTheory_pub.trimFilteredData

if opts.filterType=="fitting"

meanPs((end-SSCounter+1):(end-SSCounter+pumpTheory_pub.minTimeBeforeFit/dt))=nan;

meanFlow((end-SSCounter+1):(end-SSCounter+pumpTheory_pub.minTimeBeforeFit/dt))=nan;

else

meanPs((end-SSCounter+1):(end-SSCounter+opts.filterWindow/dt))=nan;

meanFlow((end-SSCounter+1):(end-SSCounter+opts.filterWindow/dt))=nan;

end

end

end

%Remove inital conditions needed for rungee kutta

flow = flow(time>=0);

Ps = Ps(time>=0);

PsClean = PsClean(time>=0);

Pe = Pe(time>=0);

meanPs = meanPs(time>=0);

meanFlow = meanFlow(time>=0);

target = target(time>=0);

time = time(time>=0);

%Format output data

outflowFit = fitlm(outflowData(:,1), outflowData(:,2));

if (size(outflowData(~isnan(outflowData)),1)<length(setpoints)*0.8)||isempty(outflowData(~isnan(outflowData)))

outflow = nan;

rawData = [time, flow, Ps, meanFlow, meanPs, PsClean, Pe, target];

rawData = [rawData;nan,nan,nan,nan,nan,nan,nan,nan];

else

outflow = table2array(outflowFit.Coefficients(2,1));

rawData = [time, flow, Ps, meanFlow, meanPs, PsClean, Pe, target];

end

end

function [SS, minTime] = checkSteadyState(time, Ps, flow, stepSize, SSCounter, opts, fitTimeConsts)

%Check for steady state and return result and minimum time needed before checking again (based on needing to meet a criteria continously for a certain period of time)

%time is the time vector in minutes

%Ps is the filtered pressure vector used to assess SS

%flow is the filtered flow vector used to assess SS

%stepSize is the mmHg step change in setpoint

%SSCounter is the number of simulation ticks since starting the current setpoint

%opts are the specific options used, indluing test window

%f_p is the meanFlow./meanPs but caalculated in realtime and stored to save time

%fitTimeConst are the time constants used in the fitting criteria

if nargin < 7, fitTimeConsts = [0,0]; end

SS = false;

testWin = fix(opts.testWindow/opts.dt); %convert from minutes to sim index

filtWin = fix(opts.filterWindow/opts.dt); %convert from minutes to sim index

switch strtok(opts.steadyStateCriteria,'_')

case "window" %pressure signal fulcuations must be within 10% of the stepsize

if SSCounter>=testWin+filtWin

SS = ((max(Ps(end-testWin+1:end))-min(Ps(end-testWin+1:end)))<0.1*stepSize);

minTime = 0;

else

SS = false;

minTime = time(end)+(testWin+filtWin-SSCounter)*opts.dt;

end

case "ratio" %(slopeWin) 5-min slope of flow/ps must be continuously under 0.0001 ul/min/mmHg/min for testWin minutes

steadyWin = fix(1/opts.dt); %convert from minutes to simulation index

if SSCounter>=(filtWin+steadyWin+testWin-1)

fit = fitlm(time(end-testWin+1:end),flow(end-testWin+1:end)./Ps(end-testWin+1:end));

if abs(table2array(fit.Coefficients(2,1)))<opts.ratioCriteria

[SS, minTime] =

pumpTheory_pub.optomizedRatioCheck(time(end-testWin-steadyWin+2:end),Ps(end-testWin-steadyWin+2:end),flow(end-testWin-steadyWin+2:end),testWin, opts.ratioCriteria);

minTime = time(end)+minTime*opts.dt;

else

SS = false;

minTime = time(end)+steadyWin*opts.dt;

end

else

SS = false;

minTime = time(end)+(filtWin+steadyWin+testWin-SSCounter-1)*opts.dt;

end

case "fitting" %at least 6 time constants basede on fitted time constants must have passed, and the pressure and flow fitted signal flucations must be within 1% of stepsize for testWin minutes continously

if SSCounter>=(testWin+filtWin)

percentStepSize = 0.05;

[maxPs, maxPsInd] = max(Ps(end-testWin+1:end));

[minPs, minPsInd] = min(Ps(end-testWin+1:end));

[maxFlow, maxFlowInd] = max(flow(end-testWin+1:end));

[minFlow, minFlowInd] = min(flow(end-testWin+1:end));

timeConstNum = split(opts.steadyStateCriteria,'_');

timeConstNum=str2num(timeConstNum(2));

pSS = ((maxPs-minPs)<percentStepSize*stepSize)&&(fitTimeConsts(1)*timeConstNum<(time(end)-time(end-SSCounter+1)));

fSS = ((maxFlow-minFlow)<percentStepSize*stepSize/5)&&(fitTimeConsts(2)*timeConstNum<(time(end)-time(end-SSCounter+1)));

SS = pSS&&fSS;

minTime = 0;

else

SS = false;

minTime = time(end)+(+testWin+filtWin-SSCounter)*opts.dt;

end

case "time"

SS = time(end)>=opts.testWindow;

minTime = 0;

case "true99"

SS = ((abs(Ps(end)-opts.SSPs)/(opts.SSPs-opts.SSPs_old))<=0.01)&&((abs(flow(end)-opts.SSflow)/(opts.SSflow-opts.SSflow_old))<=0.01);

minTime = 0;

otherwise

error("Not a valid SSCriteria. ('window', 'ratio', 'fitting', 'time', 'true99')");

end

if isempty(minTime), minTime=0; end

end

function [SS, minTime] = optomizedRatioCheck(time, Ps, flow, slopeWin, ratioCriteria)

%Recursivee function to check for a "testWin" minute long stretch where the 5 min slope of f/p is below 0.0001 in a more efficieent manner

%time is the time vector in minutes

%Ps is the filtered pressure vector used to assess SS

%flow is the filtered flow vector used to assess SS

%slopeWin is the window overwhich the slope should be checked (should be 5 min)

%ratioCritria is the thrshold value for the slope over the 5 min period

if nargin < 5, ratioCriteria = pumpTheory_pub.ratioCriteriaSlow; end

%If middle point still passes the SSCritereia, recursivly check the first half, then proceed to sequentially check the latter half if the entire first half is valid

ind = fix((length(time)-slopeWin+1)/2)+1;

fit = fitlm(time(ind:ind+slopeWin-1),flow(ind:ind+slopeWin-1)./Ps(ind:ind+slopeWin-1));

SS = false; minTime = ind;

if abs(table2array(fit.Coefficients(2,1)))<ratioCriteria

if ind==1, SS = true; minTime = 0; return; end %If nothing left to check, just return true

[SS, minTime] = pumpTheory_pub.optomizedRatioCheck(time(1:ind+slopeWin-2), Ps(1:ind+slopeWin-2), flow(1:ind+slopeWin-2), slopeWin, ratioCriteria);

if ~SS, return; end %If false, stop here and pass information back up through function stack

end

%Check the latter half sequntially (but backwards to find latest break in criteria) to ensure they all fit the criteria as well, if not set to false and return

for i = (length(time)-slopeWin+1):-1:(ind+1)

fit = fitlm(time(i:i+slopeWin-1),flow(i:i+slopeWin-1)./Ps(i:i+slopeWin-1));

if abs(table2array(fit.Coefficients(2,1)))>=ratioCriteria

SS = false;

minTime = i;

return;

end

end

end

function [PsFilt, flowFilt, fitParams] = filterSignals(time, Ps, flow, SSCounter, opts, fitParams)

%Perform the appropriate filtering based on the paramaters in the opts variable

%

%time is the time signal

%Ps is the pressure signal

%flow is the flow signal

%SSCounter is thee number of simulations steps taken under the current setpoint.

%opts contains thee filter type, window, turMovingAverage, and filterFlow

%Previous fitParams if available

if nargin<6, fitParams = struct('Ps', [nan,nan,nan,nan,nan], 'flow', [nan,nan,nan,nan,nan]); end

dt = opts.dt;

filtWin = round(opts.filterWindow/dt);

if filtWin==0, filtWin = 1; end

%Moving average filter applied if "mean" filter type or filterWindow timee has not passed with fitting type (not enough data to fit otherwise)

if (opts.filterType=="mean")||((opts.filterType== "fitting")&&(SSCounter < round(pumpTheory_pub.minTimeBeforeFit/dt)))

if opts.trueMovingAverage

if time(end) < filtWin*dt

PsFilt = (sum(Ps)+opts.restingIOP*(filtWin-length(Ps)))/filtWin;

if opts.filterFlow

flowFilt = sum(flow)/filtWin;

else

flowFilt=flow(end);

end

else

PsFilt = mean(Ps(max(end-filtWin+1,1):end));

if opts.filterFlow

flowFilt = mean(flow(max(end-filtWin+1,1):end));

else

flowFilt=flow(end);

end

end

else

PsFilt = mean(Ps(end-min(filtWin,SSCounter)+1:end));

if opts.filterFlow

flowFilt = mean(flow(end-min(filtWin,SSCounter)+1:end));

else

flowFilt=flow(end);

end

end

%Apply the fitting filter

elseif opts.filterType== "fitting"

if mod(SSCounter, max(1,1/(opts.fittingFrequency*60*dt)))==0

startInd = max(1,length(time)-SSCounter+1);

[PsFilt, fitParams.Ps, ~, ~] = pumpTheory_pub.fitPressure(time(startInd:end), Ps(startInd:end), fitParams.Ps(1:2));

fitParams.lastPs = PsFilt;

if opts.filterFlow

[flowFilt, fitParams.flow, ~, ~] = pumpTheory_pub.fitFlow(time(startInd:end), flow(startInd:end), fitParams.flow);

fitParams.lastFlow = flowFilt;

else

flowFilt=flow(end);

fitParams.lastFlow = flowFilt;

end

else

PsFilt = fitParams.lastPs;

flowFilt = fitParams.lastFlow;

end

end

end

function [PsFitVal, fitParams, rSquared, fitRawData] = fitPressure(t, y, beta0)

%Apply exponential fitting to pressure and extrapolate estimate of current meanIOP

%

%t is the time vector in units of minutes

%y is the pressure vector in units of mmHg

%setpoint is an estimated steady state

%beta0 is initial paramaters to use when fitting (much faster if the previous fit is used as the starting point)

% if (nargin < 3)||any(isnan(beta0)), beta0 = [5,5,1,1]; end

%assume time constant of 5 minutes and stepSize of 5 mmHg

% if beta0(2)<beta0(4)

% [beta0(1), beta0(3)] = swap(beta0(1), beta0(3));

% [beta0(2), beta0(4)] = swap(beta0(2), beta0(4));

% end

if (nargin < 3)||any(isnan(beta0)), beta0 = [5,5,0]; end

%assume time constant of 5 minutes and stepSize of 5 mmHg

if ~(beta0(1)<=20 && beta0(1) >= 0 && beta0(2) <= 50 && beta0(2) >= 0.01) %Set hard limits on starting estimates of fit paramaters

beta0 = [y(end)-y(1),5];

end

%set up model

lastResting = y(1);

model = @(b,x)lastResting + b(1).*(1-exp(-x./b(2)));% + b(3).*(1-exp(-x./b(4)));

t=t-t(1);

%set up specific fitting settings for robost fitting

opts = statset('nlinfit');

opts.RobustWgtFun = 'fair';

opts.FunValCheck = 'off';

%fit models

fitVal = fitnlm(t,y,model,beta0,'Options',opts);

rSquared = fitVal.Rsquared.Adjusted;

fitParams = table2array(fitVal.Coefficients(:,1))';

% fitParams_SE = table2array(fitVal.Coefficients(:,2))';

fitRawData=model(fitParams, [t;t(end)+t(2)-t(1)]);

fitParams = [fitParams,lastResting];

% fitParams_SE = [fitParams_SE,0];

PsFitVal=fitRawData(end);

%set up specific fitting settings for robost fitting

%opts = optimoptions('lsqcurvefit','Algorithm','levenberg-marquardt','Display','off');

% lb=[0,0.01,0,0.001];

% up=[50,50,50,50];

%

% fit models

% fitParams = lsqcurvefit(model,beta0,t,y,lb,up,opts);

% fitRawData=model(fitParams, [t;t(end)+t(2)-t(1)]);

% rSquared = corrcoef(y,fitRawData(1:end-1));

% rSquared=rSquared(1,2);

% fitParams = [fitParams,lastResting];

% PsFitVal=fitRawData(end);

function [b, a] = swap(a, b), end

end

function [PsFitVal, fitParams, rSquared, fitRawData] = fitFlow(t, y, beta0)

%Apply exponential fitting to flow and extrapolate estimate of current meanflow

%

%t is the time vector in units of minutes

%y is the pressure vector in units of mmHg

%setpoint is an estimated steady state

%beta0 is the initial parameters to use when fitting (much faster if the pervious fit is used as the starting point)

% if (nargin < 3)|| any(isnan(beta0)), beta0 = [y(1),5,0.1,1,0.1]; end

%assume time constant of 5 minutes and a steady statee flow of 0

% if beta0(2)<beta0(4)

% [beta0(1), beta0(3)] = swap(beta0(1), beta0(3));

% [beta0(2), beta0(4)] = swap(beta0(2), beta0(4));

% end

if (nargin < 3)|| any(isnan(beta0)), beta0 = [y(1),5,0]; end

%assume time constant of 5 minutes and a steady statee flow of 0

if ~(beta0(1)<=20 && beta0(1) >= 0 && beta0(2) <= 50 && beta0(2) >= 0.01) %Set hard limits on starting estimatees of fit paramaters

beta0 = [y(1),5,0];

end

%set up model

lastResting = y(1);

model = @(b,x)b(1).*exp(-x./b(2))+b(3);%+b(3).*exp(-x./b(4))+b(5);

t=t-t(1);

%set up specific fitting settings for robost fitting

opts = statset('nlinfit');

opts.RobustWgtFun = 'fair';

opts.FunValCheck = 'off';

%fit models

fitVal = fitnlm(t,y,model,beta0,'Options',opts);

rSquared = fitVal.Rsquared.Adjusted;

fitParams = table2array(fitVal.Coefficients(:,1))';

%fitParams_SE = table2array(fitVal.Coefficients(:,2))';

fitRawData=model(fitParams, [t;t(end)+t(2)-t(1)]);

PsFitVal=fitRawData(end);

% set up specific fitting settings for robost fitting

% opts = optimoptions('lsqcurvefit','Algorithm','levenberg-marquardt','Display','off');

% lb=[0,0.01,0,0.001,min(y)];

% up=[100,50,100,50,max(y)];

%

% fit models

% fitParams = lsqcurvefit(model,beta0,t,y,lb,up,opts);

% fitRawData=model(fitParams, [t;t(end)+t(2)-t(1)]);

% rSquared = corrcoef(y,fitRawData(1:end-1));

% rSquared=rSquared(1,2);

% PsFitVal=fitRawData(end);

function [b, a] = swap(a, b), end

end

function outflowOpt = createOutflowOptions(mode, SSCriteria, testWin, filtWin, filtTyp, noiseSig, noiseStd, timeout, flowLim, restingIOP, filtFlow, fitFreq, trueMovAvg, downSample, ratioCriteriaThresh)

%Create a standardized struct for the opts argument in the outflow function

%

% testWin is the window over which to apply and test for steady state criteria

% filtWin is the moving average filter length in minutes

% filtTyp specifies a moving average filter ("mean") or a fitting filter ("fitting")

% noiseSig is a mean corrected noise record that can be used to simulate real collected noise data, samplig freq should match with simulation freq

% noiseStd is the standard deviation in mmHg of gaussian noise to be added to PsClean to give Ps records

% timeout is the maximum time for a given setpoint to reach stady state before terminating and ignoring the measurment point

% flowlim is a vector of 2 number specifiying the loweer and upper limit of the flow that can be produced, to simulate real pump limitations

% restingIOP is the resting IOP or Pe for the eye based on literature values.

% filtFlow is a boolean flag indicating if the flow signal should also be filtered

% fitFreq is th frequency at which to fit th data when using a fitting filter, help prevent fitting too often and slowing down the program

% trueMovAvg is a boolean flag indicating if a tru moving average should be used, see definition under class properties

% downSample speficies the frequency at which to downsample to when using a fitting filter to making fitting faster.

% ratioCriteriaThresh specifies the cuttoff slope value useed in the ratio criteria, and should be tuned to fedback dynamics for optimaal performance. Larger threshold value, or easier triggering should be used with faster systems

if nargin<1, error("Needs at least one arg; mode"); end

%Catch an error

if nargin<2, SSCriteria = "true99"; end

%SSCriteria is the criteria used to determine when SS is reached

if nargin<3, testWin =

5*(SSCriteria=="window"||SSCriteria=="ratio")+2*(SSCriteria=="fitting")+0;

end %units of minutes; window which to check stop criteria. Default values for each SSCriteria are applied here if not specified

if nargin<4, filtWin = 0; end

%units of minutes, when fitting filter is used, this will dictate the minimum time befor fitting may applied

if nargin<5, filtTyp = 'mean'; end

%'mean' for moving average and 'fitting' for fitting filter

if nargin<6, noiseSig = []; end

%vector of mean corrected noise, ensure timestep matches simulation

if nargin<7, noiseStd = 0; end

%std for gaussian noise to be added to signal

if nargin<8, timeout = 90; end

%timeout in minutes for each setpoint

if nargin<9, flowLim = []; end

%[min max] in ul/min; min and max flow rates to simulate pump limitations

if nargin<10, restingIOP = 15; end %resting IOP in mmHg

if nargin<11, filtFlow = ~((mode=="CPpx")&&~pumpTheory_pub.CPpx_postFiltering); end

%boolean flag whether to filter flow signal as well or not

if nargin<12, fitFreq = 1; end

%How often to fit data (Hz) using fitting filter, anything >1 is bit unnecessary

if nargin<13, trueMovAvg = pumpTheory_pub.trueMovingAvg; end

%see definitionin class properties

if nargin<14, downSample = fitFreq*(filtTyp=="fitting"); end

%Downsample to (Hz) before filtering for faster fitting times since nonlinear fit

if nargin<15, ratioCriteriaThresh = pumpTheory_pub.ratioCriteria; end

%RatioCriteria based on time constant of feedback system used for best performance.

%set default filtWin and testWin for fitting filter

if (filtTyp=="fitting")&&(nargin<3), filtWin=2; testWin=2; end

%fix timeout if it is too low

if timeout <= testWin, timeout = testWin+1;end

%Add in a gain for safekeeping and ease of access

switch mode

case "CF", k=nan;

case "CPg1", k=1/pumpTheory_pub.CPg1_Rs;

case "CPg2", k=1/pumpTheory_pub.CPg2_Rs;

case "CPp", k=pumpTheory_pub.CPp_k;

case "CPpx", k=pumpTheory_pub.CPp_k;

otherwise

k=nan;

if ~contains(lower(mode),"custom"), error("Not a valid mode."); end

end

%Create the struct from defaults and passed arguments

outflowOpt = struct('gain',k,'steadyStateCriteria',SSCriteria,'testWindow',testWin,'filterWindow',filtWin,'filterType',filtTyp,'noiseSignal',noiseSig,'noiseStd',noiseStd,'timeout',timeout,'flowLimit',flowLim,'restingIOP',restingIOP, 'filterFlow', filtFlow, 'fittingFrequency', fitFreq, 'trueMovingAverage', trueMovAvg, 'downSample', downSample, 'ratioCriteria', ratioCriteriaThresh);

end

function adjNoise = interpolateNoise(dt, time, noise)

%Create noise signal with the same timestep as sim, simply interpolating signal

%

% dt i thee timestep in minutes for the desired signal

% time is the time vector for the noise signal

% noise is the noise signal vector

if length(time)~=length(noise),

error("time and noise must have the same length");

end

timeAdj = (0:dt:time(end))';

for ind = 1:size(noise,2);

adjNoise(:,ind) = interp1(time, noise(:,ind), timeAdj);

end

end

function noise = genNoise(arg)

%Generates a white noise sample with a cuttoff frequency of 0.4Hz

s1=normrnd(arg.mu,arg.st,round(arg.len/arg.dt),1);

t1=(0:arg.dt:arg.len-arg.dt)';

noise=movmean(s1,round(1.2/(60*arg.dt)));

noise=normrnd(1,0.05)*noise*arg.st/std(noise);

noise = [t1,noise];

end

function [SS_Ps, SS_Flow] = getSteadyState(mode, setpoint, opts)

%Solve for steady state pressure given mode/setpoint accounting for setpoint error

%

%mode is the model used for the simulation

%setpoint spcifies the setpoint in mmHg

%opts contains the options paramter

restingIOP = opts.restingIOP;

%Set up equations for pressure flow relationships at steady state baseed on model

syms p_s

switch mode

case "CF"

eqn = p_s/(pumpTheory_pub.rt+pumpTheory_pub.rc) == setpoint;

case "CPg1"

eqn = p_s/(pumpTheory_pub.rt+pumpTheory_pub.rc) == (setpoint-restingIOP-p_s)/pumpTheory_pub.CPg1_Rs;

case "CPg2"

eqn = p_s/(pumpTheory_pub.rt+pumpTheory_pub.rc) == (setpoint-restingIOP-p_s)/pumpTheory_pub.CPg2_Rs;

case "CPp"

eqn = p_s/(pumpTheory_pub.rt+pumpTheory_pub.rc) == (setpoint-restingIOP-p_s)*pumpTheory_pub.CPp_k;

case "CPpx"

eqn = p_s/(pumpTheory_pub.rt+pumpTheory_pub.rc) == (setpoint-restingIOP-p_s)*pumpTheory_pub.CPp_k;

otherwise

if contains(lower(mode),"custom")

eqn = p_s/(pumpTheory_pub.rt+pumpTheory_pub.rc) == (setpoint-restingIOP-p_s)*opts.customK;

else

error("Not a valid mode.");

end

end

%Solve and format for output

SS_Ps=double(solve(eqn,p_s,'Real',true));

SS_Flow = SS_Ps/(pumpTheory_pub.rc+pumpTheory_pub.rt);

SS_Ps = SS_Ps + restingIOP;

end

function f = getFlowEq(mode, customK)

%Determine appropriate flow in response to the pressure signal based on model type

%

%mode: model used for the simulation

%customK: optional parameter to specify a custom gain for proportional feedback system (both CPg and CPp models fall into this)

if nargin >= 2

if contains(mode, "CPpx")

f=@(s,p,m)(s-m)*customK;

else

f=@(s,p,m)(s-p)*customK;

end

return;

end

switch mode

case "CF"

f=@(s,p,m)s;

case "CPg1"

f=@(s,p,m)(s-p)/pumpTheory_pub.CPg1_Rs;

case "CPg2"

f=@(s,p,m)(s-p)/pumpTheory_pub.CPg2_Rs;

case "CPp"

f=@(s,p,m)(s-p)*pumpTheory_pub.CPp_k;

case "CPpx"

f=@(s,p,m)(s-m)*pumpTheory_pub.CPpx_k;

otherwise

f=Nan;

end

end

function [finalFilterWidth, noiseStdInd] = getFilterWidth(targetNoisePath, noisePath, modes, tolerance, freq)

if nargin<4, tolerance=0.00001; end

if nargin<5, freq=10; end

restingIOP=15;

%Load in noise samples to set the target noise std.

load(targetNoisePath, "noise");

for noiseInd=1:length(noise)

noiseTargetStd(noiseInd,1)=std(noise(noiseInd).data(:,2)./1000./noise(noiseInd)

.data(:,3));

noiseTargetStd_F(noiseInd,1)=std(noise(noiseInd).data(:,2)./1000);

noiseTargetStd_P(noiseInd,1)=std(noise(noiseInd).data(:,3));

end

targetStd = mean(noiseTargetStd);

%Load in noise samples to be compared

noise = readmatrix(noisePath);

noiseTime = noise(:,1);

noise = noise(:,2:end);

%loop through different modls and noise samples to return required filterWidth to filter noise down to exVivo range

for modeInd=1:length(modes)

opt = pumpTheory_pub.createOutflowOptions(modes(modeInd), "time", 60, 0, "mean", [0]);

%Constants needed for filtrWidth search algo

noiseStd = 0;

filterWidth = 0;

minFilterWidth = filterWidth;

maxFilterWidth = filterWidth;

%Loop and iteratively change filterWidth to bring std of flow./pressure to target

while true

opt.filterWindow=filterWidth;

noiseStdInd(modeInd,:)=nan;

noiseStdInd_P(modeInd,:)=nan;

noiseStdInd_F(modeInd,:)=nan;

%Run model and calculate the noiseStd across all noise samples

for noiseInd=1:size(noise,2)

noiseEx=rmmissing(noise(:,noiseInd));

opt.testWindow=noiseTime(length(noiseEx));

noiseEx = pumpTheory_pub.interpolateNoise(1/(freq*60), noiseTime(1:length(noiseEx)), noiseEx);

opt.noiseSignal=noiseEx;

[~, ~, rawData] = pumpTheory_pub.outflow(modes(modeInd), restingIOP, freq, opt);

%SS_indicies = rawData(:,1)>10;

noiseStdInd(modeInd,noiseInd)=std(rmmissing(rawData(:,4)./rawData(:,5)));

noiseStdInd_F(modeInd,noiseInd)=std(rmmissing(rawData(:,4)));

noiseStdInd_P(modeInd,noiseInd)=std(rmmissing(rawData(:,5)));

end

noiseStd=mean(noiseStdInd(modeInd,:));

if abs(noiseStd-targetStd)<tolerance

break

else

if isnan(noiseStd)

filterWidth=nan;

break;

end

if filterWidth==0

if noiseStd < targetStd

break;

end

filterWidth = 1;

maxFilterWidth = filterWidth;

else

if maxFilterWidth-minFilterWidth<=2/60

filterWidth=mean([maxFilterWidth,minFilterWidth]);

break;

end

if noiseStd > targetStd

if filterWidth>=maxFilterWidth

filterWidth=2*filterWidth;

maxFilterWidth=filterWidth;

else

minFilterWidth=filterWidth;

filterWidth=mean([filterWidth,maxFilterWidth]);

end

else

if filterWidth<=minFilterWidth

error("Code is broken, should not be here");

else

maxFilterWidth=filterWidth;

filterWidth=mean([filterWidth,minFilterWidth]);

end

end

end

end

filterWidth=round(filterWidth*60)/60;

end

finalFilterWidth(modeInd,1)=filterWidth;

end

end

end

end
